# Visual mismatch negativity in Parkinson's psychosis and potential for testing treatment mechanisms

**DOI:** 10.1093/braincomms/fcae291

**Published:** 2024-09-03

**Authors:** Miriam Vignando, Dominic ffytche, Ndabezinhle Mazibuko, Giulio Palma, Marcella Montagnese, Sonali Dave, David J Nutt, Anthony S Gabay, Yen F Tai, Lucia Batzu, Valentina Leta, Caroline H Williams Gray, K Ray Chaudhuri, Mitul A Mehta

**Affiliations:** Centre for Neuroimaging Sciences, Institute of Psychiatry, Psychology and Neuroscience, King’s College London, London, SE5 8AF, UK; Institute of Psychiatry, Psychology and Neuroscience, King’s College London, London, SE5 8AF, UK; Centre for Neuroimaging Sciences, Institute of Psychiatry, Psychology and Neuroscience, King’s College London, London, SE5 8AF, UK; Department of Psychology, University of Southampton, Southampton, SO17 1PS, UK; Department of Clinical Neurosciences, University of Cambridge, Cambridge, CB2 2PY, UK; Department of Optometry and Visual Sciences, City, University of London, London, EC1V 0HB, UK; Imperial College London, Faculty of Medicine, Department of Brain Sciences, Burlington Danes, The Hammersmith Hospital, London W12 0NN, UK; IXICO, London, EC1A 9PN, UK; Imperial College London, Faculty of Medicine, Department of Brain Sciences, Charing Cross Hospital, London W6 8RF, UK; Parkinson Foundation Centre of Excellence, King's College Hospital NHS Foundation Trust, SW9 8R, London, UK; Parkinson Foundation Centre of Excellence, King's College Hospital NHS Foundation Trust, SW9 8R, London, UK; Department of Clinical Neurosciences, Parkinson and Movement Disorders Unit, Fondazione IRCCS Istituto Neurologico Carlo Besta, Milan, 20133, Italy; John Van Geest Centre for Brain Repair, Department of Clinical Neurosciences, University of Cambridge/Cambridge University Hospitals NHS Foundation Trust, Cambridge, CB2 0PY, UK; Parkinson Foundation Centre of Excellence, King's College Hospital NHS Foundation Trust, SW9 8R, London, UK; Centre for Neuroimaging Sciences, Institute of Psychiatry, Psychology and Neuroscience, King’s College London, London, SE5 8AF, UK

**Keywords:** mismatch negativity, Parkinson’s disease, Parkinson’s psychosis, 5-HT_2A_, visual hallucinations

## Abstract

Psychosis and visual hallucinations are a prevalent non-motor symptom of Parkinson's disease, negatively affecting patients’ quality of life and constituting a greater risk for dementia. Understanding neural mechanisms associated to these symptoms is instrumental for treatment development. The mismatch negativity is an event-related potential evoked by a violation in a sequence of sensory events. It is widely considered an index of sensory change-detection. Reduced mismatch negativity response is one of the most replicated results in schizophrenia and has been suggested to be a superior psychosis marker. To understand whether this event-related potential component could be a similarly robust marker for Parkinson's psychosis, we used electroencephalography with a change-detection task to study the mismatch negativity in the visual modality in 20 participants with Parkinson's and visual hallucinations and 18 matched Parkinson's participants without hallucinations. We find that visual mismatch negativity is clearly present in participants with Parkinson’s disease without hallucinations at both parieto-occipital and frontal sites, whereas participants with Parkinson's and visual hallucinations show reduced or no differences in the two waveforms, confirming the sensitivity of mismatch negativity to psychosis, even within the same diagnostic group. We also explored the relationship between hallucination severity and visual mismatch negativity amplitude, finding a negative correlation between visual hallucinations severity scores and visual mismatch negativity amplitude at a central frontal and a parieto-occipital electrodes, whereby the more severe or complex (illusions, formed visual hallucinations) the symptoms the smaller the amplitude. We have also tested the potential role of the serotonergic 5-HT_2A_ cascade in visual hallucinations in Parkinson's with these symptoms, following the receptor trafficking hypothesis. We did so with a pilot study in healthy controls (*N* = 18) providing support for the role of the Gi/o-dependent pathway in the psychedelic effect and a case series in participants with Parkinson's and visual hallucinations (*N* = 5) using a double-blind crossover design. Positive results on psychosis scores and mismatch amplitude add further to the potential role of serotonergic modulation of visual hallucinations in Parkinson's disease.

## Teaser

Vignando *et al*. report that visual mismatch negativity (vMMN) is a robust marker for sensory change detection in Parkinson’s disease with visual hallucinations and a relationship between hallucination severity and vMMN. The authors provide support for a potential role of serotonergic modulation of hallucinations in Parkinson’s with a case series.

## Graphical Abstract

**Graphical Abstract:**
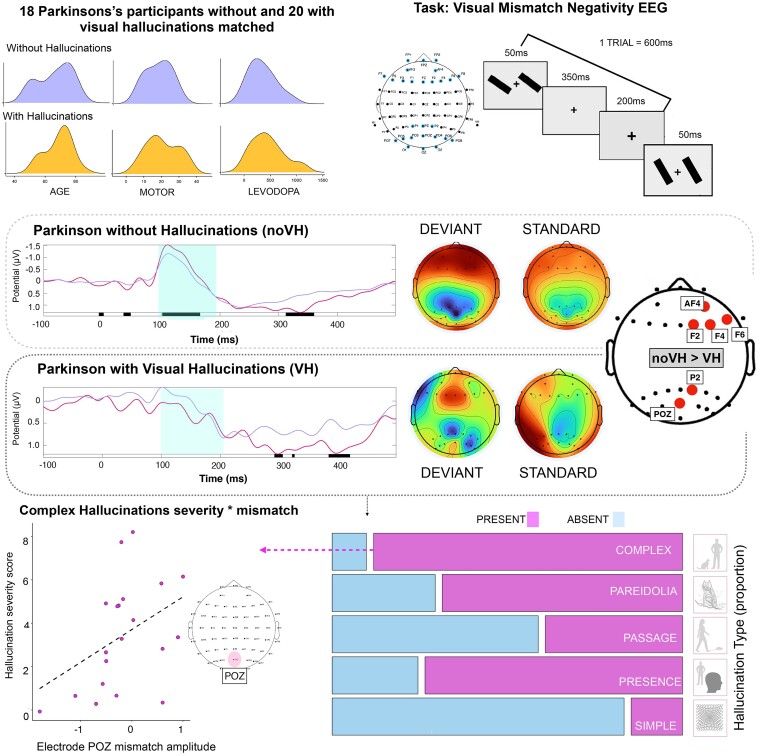

## Introduction

The mismatch negativity (MMN), an event-related potential evoked when there is a violation in a sequence of sensory events, is widely considered an index of sensory change-detection,^1^ and as a perceptual prediction error.^2^ Despite the wealth of research on MMN, the biological underpinnings of the MMN are not completely understood, but several hypotheses have been presented. The most influential one is predictive coding^3-5^—a computational mechanism whereby event-related responses are proposed to be an expression of the brain attempting to minimize prediction errors. Within this framework, the MMN is considered an index of the neural activity reporting such a prediction. Using hierarchical Bayesian inference, the brain is proposed to work out the causality of sensory inputs by inverting the process that generated such inputs from the environment and to resolve discrepancies such as that produced by the MMN. This view has been used to explain the disruption of MMN in psychosis.^6-8^ Indeed, a reduced auditory MMN response is one of the most replicated results in schizophrenia.^9,10^ Initially, the MMN was thought to be present only for the auditory modality, but it has been demonstrated for the somatosensory^11^ and visual modalities, with the visual MMN (vMMN) also reduced in schizophrenia.^12-14^

Psychosis also occurs in Parkinson's disease as one of the most common non-motor aspects.^15,16^ Psychosis in Parkinson's follows a behavioural continuum, with minor hallucinations (including illusions) presenting first, followed by fully formed visual hallucinations (VH).^17^ Dopamine agonists play a role in these symptoms, but are not the only factor, as different studies show that up to 42% of drug naïve patients show forms of VH, with percentages ranging from 12% to 26% to 42% (^16^for a review). These results suggest that despite the involvement of such medication, this cannot be the only mechanism underlying this cluster of symptoms. Studies of MMN in Parkinson's without psychosis have been limited to the auditory domain and have shown a normal MMN amplitude in patients at the early stages of the disease.^18^ As Parkinson's disease progresses, a smaller amplitude can be observed.^19^ To the best of our knowledge, there are no reports of the MMN in patients meeting the criteria Parkinson's disease psychosis (PDP) in the auditory or visual domain. As hallucinations in PDP are visual, we suggest vMMN is a more appropriate test within the framework of predictive processing.^20^

Functional neuroimaging studies investigating VH show widespread alterations in brain function, including dysfunction of the ventral visual pathway.^16^ Structural imaging studies also implicate brain regions outside of visual processing areas, including parietal, frontal, hippocampal and cerebellar regions^17,21^ together with the involvement of regions of the attentional control networks (i.e. the default mode and the dorsal and ventral attentional networks).^21,22^ Together, these studies fit with models of PDP proposing both disrupted bottom-up processing of sensory information and dysfunctional top–down influences on perceptions.^22^ A recent fMRI dynamic causal modelling study of patients with PDP showed that differences between patients with and without VH were best explained when taking into account diminished visual (bottom–up) and dysfunctional top–down (prefrontal cortex to thalamus) processes, adding further to the predictive coding hypothesis—proposing a strong role of incorrect priors from top–down, prefrontal, areas.^23^ This supports our hypothesis that that vMMN might help identify specific neural mechanisms associated with VH in PDP.

Analyses using receptor density maps suggest that grey matter loss in PDP is linked to the 5-HT2_A_ receptor distribution.^21^ Two studies have directly tested striatal and extra-striatal serotonin markers in PDP. Direct measurement of 5-HT2_A_ receptor availability has produced mixed findings with two studies showing an increase in binding in the ventral visual pathway, and one showing a decrease in binding. The increased binding was shown in one *in vivo* PET study using [18F]-setoperone^24^ and a post-mortem study using [3H]-ketanserin.^25^ The decrease in binding was in a group of PDP with cognitive deficits showing a decrease in [18F]-setoperone binding, which correlated with cognitive function.^26^

Several lines of evidence have linked 5-HT_2A_ receptors to visual processing and hallucinations. First, 5-HT_2A_ receptors are highly expressed in the visual cortex layers I, IV and V^27^ and altered 5-HT2_A_ activity in layer V modulates alpha wave activity,^28^ involved in both visual processing and VH. Second, 5-HT_2A_ receptor agonism with psychedelics produces altered visual experiences, which can include hallucinations^28,29^ and these can be blocked with ketanserin, a 5-HT_2A_ receptor antagonist. Ketanserin also reduces N170 VEPs that are associated with the visual perceptual alterations produced by psilocybin.^28^ The receptor trafficking hypothesis provides a reason why only some 5-HT_2A_ agonists lead to altered visual experiences. The specific proposal is that psychedelic 5-HT_2A_ agonists act through the 5-HT_2A_R/mGlu_2_R heterocomplex and activate Gi/o-dependent signalling as well as Gq/11.^30,31^ We reasoned that by targeting the Gi/o-dependent pathway (through SRC-kinase inhibition) we could reduce the psychedelic effects of psilocybin in healthy volunteers, conferring the potential to reduce VH in Parkinson's disease (PD). In rodents, SRC-kinase inhibition blocked 5-HT_2A_ receptor activation induced head twitches associated with a ‘hallucinogenic’ response.^31^ In this paper, we present the results of a study conducted in healthy volunteers to test if the SRC-kinase inhibitor ‘saracatinib’ attenuates the psychedelic experience in humans, to translate the findings from experimental animals^32^ before conducting a pilot study in patients with PD with VH (PD-VH).

Overall, we hypothesize vMMN will be affected in PDP/PD-VH, similar to what is observed in other psychoses.^20^ Thus, we expect to find a difference of the vMMN in patients with hallucinations when compared to patients without hallucinations. We expect this to be present at different EEG channels. Studies using auditory stimuli have led to propose separate temporal dynamics of aMMN production, with a frontal and a temporal component.^33,34^ The two components have also been proposed to reflect the MMN arising from the comparison of sensory inputs and top–down predictions that rely on a memory trace.^35,36^

Thus, we also hypothesize a similar pattern for vMMN with a frontal and parieto-occipital components. We also hypothesize that ‘saracatinib’ would reduce behavioural measures of the psychedelic experience. Finally, we test the hypothesis that ‘saracatinib’ can modulate vMMN in PD with hallucinations via SRC-kinase inhibition. We also test the hypothesis that this modulation of vMMN is accompanied by a reduction of VH.

## Materials and methods

For all three studies, approval was received by the Research and Development office of KCL and the NHS Trusts. Ethics approval was granted by the KCL Research Ethics Committee for the healthy volunteer study (PNM/14/15-11) and the Health Research Authority ethics committee for the studies in patients (18/LO/2144). Written informed consent was obtained prior commencing study procedures following the Declaration of Helsinki.

### Study 1: PD-VH versus PD-noVH vMMN EEG study

Patients with Parkinson's disease without (PD-noVH) and with VH (PD-VH) took part in this study. Participants were recruited through the Parkinson's UK research hub, the Movement Disorder clinics at the Parkinson's center of excellence at King's College Hospital (KRC), Charing Cross Hospital (YT), Cambridge University Hospitals NHS Trust (CWG) and from a previous KCL study.^37^ We enrolled 19 PD-noVH and 21 PD-VH, but one participant from each group did not have usable data from one of the main electrodes (POZ). The final sample comprised 18 PD-noVH and 20 PD-VH. All PD patients were tested while ‘on’ their dopaminergic medication.

After a phone pre-screening, participants attended one study day at the clinical research facility (CRF) of King's College Hospital where they completed screening, for which we collected medical history, medication, physical and non-motor symptoms examinations (see Supplementary Information 1 for a complete description and Table 1), cognitive testing and conducted the EEG session.

**Table 1:** Participants’ demographics and clinical information

	Group	Mean	Standard deviation	*P*
Age	PD-NOVH	65.74	10.42	0.320
	PD-VH	68.85	8.60	
Disease duration	PD-NOVH	4.57	2.84	0.125
	PD-VH	6.28	3.73	
LEDD^a^	PD-NOVH	352.17	248.50	0.276
	PD-VH	454.13	303.78	
MoCA	PD-NOVH	27.17	2.79	0.934
	PD-VH	27.10	2.79	
MoCA-attention	PD-NOVH	0.97	0.06	0.20
	PD-VH	0.93	0.13	
MoCA-memory	PD-NOVH	3.53	1.67	0.33
	PD-VH	4	1.05	
NMSS	PD-NOVH	7.47	3.48	<0.001
	PD-VH	14.44	3.33	
SCOPA-motor^b^	PD-NOVH	17.88	7.29	0.310
	PD-VH	20.72	9.14	
CISI-PD	PD-NOVH	6.35	3.28	0.071
	PD-VH	8.5	3.78	
CISI-PD motor	PD-NOVH	2.47	0.94	0.79
	PD-VH	2.55	0.83	
CISI-PD motor complications	PD-NOVH	1.29	1.36	0.29
	PD-VH	1.79	1.40	
CISI-PD cognitive	PD-NOVH	0.65	0.86	0.02
	PD-VH	1.60	1.35	
CISI-PD disability	PD-NOVH	1.94	0.90	0.05
	PD-VH	2.60	1.05	

We report disease duration (in years), LEDD (levodopa equivalent dose^a^), Montreal Cognitive assessment (MoCA) scores (participants were not included if they presented a score lower to 22), SCOPA-motor^b^ total score, NMSQ (non-motor symptom questionnaire) total score, clinical impression of severity index for PD, completed by a Parkinson’s neurologist upon physical exam and the breakdown of the CISI-PD in the four categories (motor severity, motor complications, cognitive status and disability; cognitive status and disability are computed based on the self-reported deficits of the patients). The statistics presented are the result of a one-way ANOVA with group (VH-noVH) as between-participants factor.

^a^LEDD: one PD-noVH participant did not have this information but was on Sinemet and Madopar—we used the group average of PD-noVH for this analysis.

^b^One PD-noVH participant did not have a SCOPA-motor or UPDRS-III score and was excluded from this comparison.

To investigate VH, participants’ partners completed the hallucinations and delusions scale of the Neuropsychiatric Inventory,^38^ and PD-VH participants were administered the scale for the assessment of positive symptoms-PD (SAPS-PD) adapted for PD from schizophrenia^39^ and an adaptation of the North–East Visual Hallucinations Interview (NEVHI), a semi-structured interview to assess the phenomenology of VH.^40^ As there are no specific scoring rules for the adapted version of the NEVHI, we computed temporal severity by multiplying duration and the frequency of the VH, using an ordinal scale. We also computed a continuous temporal severity score multiplying the raw number of minutes spent hallucinating by the raw number of VH in a month (see Supplementary Information 1). Study day assessments and descriptions of the questionnaires are reported in Supplementary Information 1 and Table 1.

**Figure 1:**
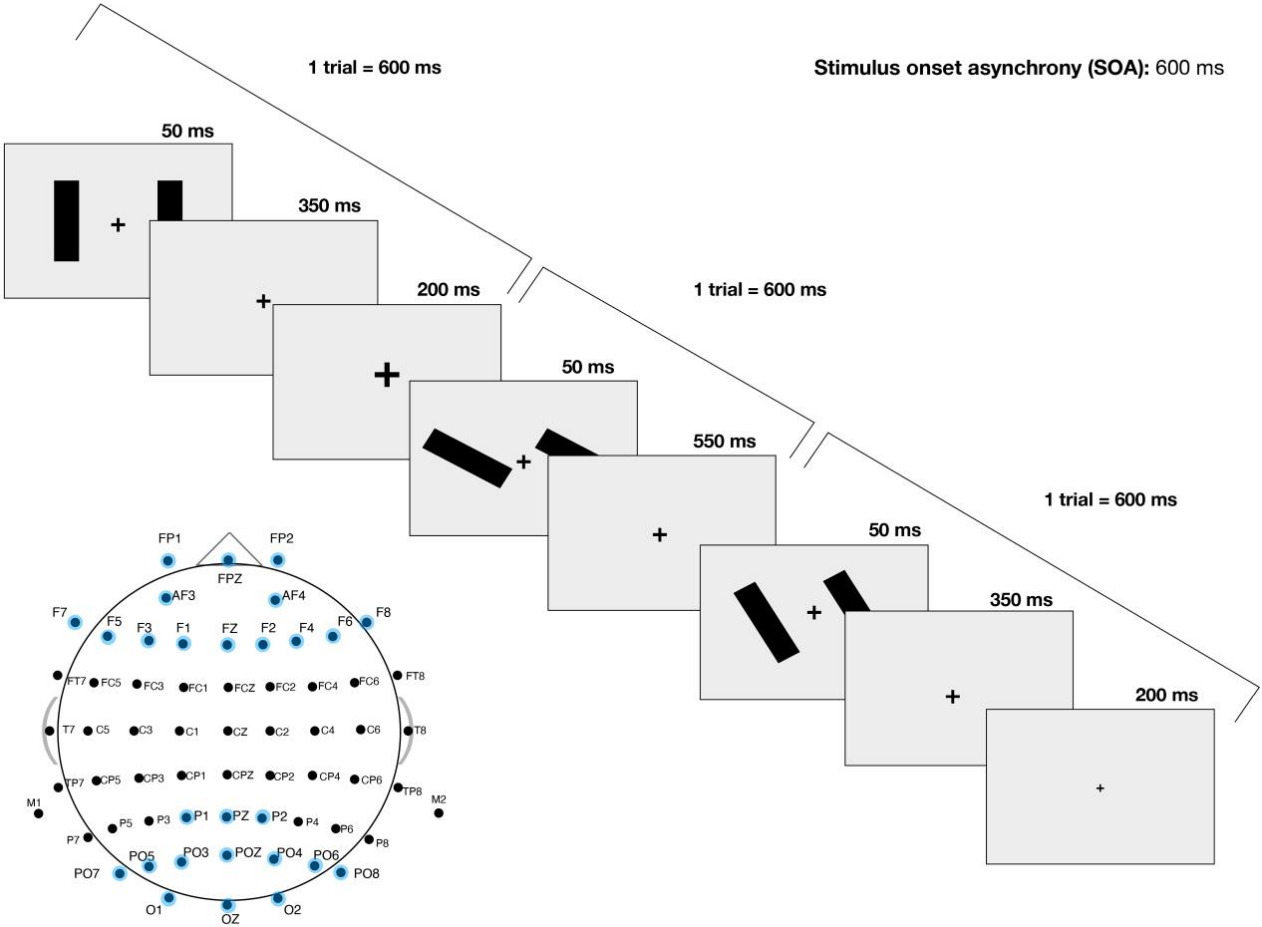
**Visual summary of the vMMN task**. vMMN (visual mismatch negativity) task sequence of events and channels of interest in our analyses.

The vMMN task (Presentation v.17.2) required participants to decide via button press when the fixation cross at the centre of the screen changed in size, while ignoring peripheral stimuli. The task was developed from one of the paradigms presented in Qian *et al*.^41^ and is described in detail together with the EEG acquisition protocol in and Supplementary Information 2–5 (see Fig. 1 for a visual summary and Supplementary Information 2, 3 for pilot data and task data for Study 1). To acquire the EEG data, we used two Compumedics Neuroscan 64-electrode EasyCaps, which were alternated across participants within each group; caps had sintered Ag–AgCl sensors and the SynAmps RT amplifier, with patients being seated at 74–76 cm from the CRT screen. Impedances of all electrodes were kept at 15 kΩ or below. Signals were recorded using Scan 4.5 software. A sampling rate of 1000 Hz was used for the recording.

#### Statistical analysis

Descriptive statistics for sociodemographic and clinical information are reported in Table 1. None of our patients was on quetiapine. One PD-VH patient was on sertraline and one PD-noVH on citalopram (SSRIs). One PDP patient was on clonazepam and one on clozapine. One PD patient was on lamotrigine and another one on clonazepam (details in Supplementary Information 4).

Thirteen out of 38 participants had a phone screening during the pandemic (instead of in-person). For this, we administered the blind MoCA (Montreal Cognitive Assessment) and converted the score to the full MoCA.^42^ On the study day, the full MoCA was also administered to confirm eligibility (MoCA > 22), but the initial score was retained for analysis to minimize the influence of practice effects.

For one of the participants, it was not possible to administer the SCOPA-motor (part of Scales for Outcomes in Parkinson's disease). For this participant, we had a recent full UPDRS (Unified Parkinson's Disease Rating Scale) (<2 weeks), and the score was converted using a method described previously.^43^

Data were pre-processed with EEGLAB v2021.1^44^ and ERPLAB v8.3.0^45^ (Matlab version R2022b, Mathworks, Inc.) using the standard EEGlab pre-processing pipeline. A detailed description is reported in Supplementary Information 4.

Statistical data analysis was carried out in R version 4.0.3,^46^ using the following packages: dplyr,^47^ ggcorrplot,^48^ PairedData,^49^ ICcmodavg,^50^ tidyverse,^51^ ggpubr^52^ and broom,^53^ and SPSS Version 28.0. Armonk, NY: IBM Corp.

First, we carried out a within group analysis of standard and rare deviant ERP components performed using a script within EEGlab's STUDY structure.^44,45^ A set of power spectra and event-related measures for each dataset were computed, with rare deviant/standard being entered as condition for PD-VH and PD-noVH separately in a one-way ANOVA design. Multiple comparisons across channels were false discovery rate corrected (*P* < 0.05) (additional details in Supplementary Information 8). With the same method, we explored the presence of the P300 (P3a and P3b), to confirm participants’ engagement with the task. We note that the typical MMN elicited frontally by aMMN is here parieto-occipital and frontally we have a positive mismatch, thus the P3b found posteriorly here might as well reflect processes usually associated with the P3a. The same procedure was used for the between-group analysis conducted with both PD-VH and PD-noVH whereby standard/deviant was entered as condition and PD-VH/PD-noVH as group in a two-way mixed ANOVA design.

##### ERP component analysis and vMMN measurements

The auditory MMN (aMMN) is traditionally expressed at fronto-central electrodes, typically at 100–250 ms after stimulus onset. However, results vary across studies when latency is considered, with some reporting a MMN as early as 90 ms and others as late as 400 ms.^20^ Here, we observed an earlier (100–125 ms) MMN at parieto-occipital sites and a more extended (100–180 ms) MMN at frontal sites. Difference waves between the standard and individual deviant conditions were generated for each participant for the time interval considered. The difference waves were then summed to obtain the difference wave in the time interval of the MMN. The interval was defined by plotting the grand average standard and rare deviant waveforms and identifying the vMMN. In addition, individual waveforms were visually inspected to make sure that the interval selected took into account inter-individual variability. We also used the EEGlab study analyses to confirm the time interval selected for the vMMN amplitude analysis.

Once the average vMMN amplitude was estimated for each participant we compared PD and PD-VH with ANOVAs in R. First, we checked the distribution of the data at each channel of interest. For this analysis, we focused on the electrodes where the waveform presented the typical features of an MMN, thus the negative peak followed by a P300 component. We analysed data from specific parieto-occipital and frontal electrodes, based on the results from the EEGlab study.

For the correlational analyses between hallucinations measures and MMN, we ran parametric or non-parametric tests as appropriate. We first checked whether SAPS-PD or NEVHI scores correlated with any of the other clinical variables (LEDD—levodopa equivalent dose, disease duration, SCOPA-MOTOR, age, MoCA and non-motor symptoms). To test the relationship between the severity of VH (SAPS-PD and NEVHI) and MMN, we only analysed the PD-VH group. For the NEVHI, we focussed on complex VH and ran the analysis both in all 20 participants and in the 17 who presented complex VH. We used one-tailed correlations as our hypotheses were that people with more severe hallucinations would have a more altered (reduced) mismatch.

We focussed on electrodes where we found a difference between the groups. For frontal electrodes, as some but not all participants had a positive mismatch wave, we used a signed MMN index (zero when the deviant and standard were the same). For POZ, we kept the raw scores as no participants had a positive mismatch wave at parieto-occipital electrodes. To make sure that what we were measuring was indeed (rare deviant—standard), we used individual peaks rather than the sum of amplitudes over a specific interval. This allowed us to accommodate inter-individual differences in this VH-only sample.

##### Task data analysis

Task data analysis were carried out to validate the task (pilot analysis in Supplementary Information 2) and to analyse participants’ performance at the cross-change detection task. PD-noVH patients performed better than PD-VH at the distractor task (Supplementary Information 3 for details).

### Study 2: Saracatinib and psilocybin behavioural study in healthy volunteers

There were two main predictions of this study, which were that a single oral dose of 125 mg saracatinib would attenuate the subjective effects of 2 mg psilocybin infusion and reduce neuroimaging markers. Here, we present the subjective outcomes to validate the use of this drug for the patient pilot study. Detailed methods for this study are reported in Supplementary Information 6.

### Study 3: PD-VH only saracatinib pilot study

Of 42 potential participants identified, 25 met criteria and were phone pre-screened. Of them, 11 were eligible for the drug study and seven passed the on site screening and were enrolled into the study, with 5 completers. The drug study was not completed due to interruption of drug manufacturing due to reasons unrelated to the study or safety concerns (see Supplementary information 7 for additional details and detailed experimental design and procedure). Here, we present the results as a pilot case series. In depth methods for this study are reported in Supplementary Information 12.

Those enrolled were randomized to take a daily oral dose of 100 mg of saracatinib every morning for 14 days (+/−2 days) for one study period and matched placebo for the other study period within a double-blind study design. Order was unblinded after the EEG data were pre-processed and ERP data extracted.

The same clinical data were collected as Study 1 for all visits. A secondary aim of the study was to assess whether any of the psychosis symptoms change with the administration of saracatinib, which were compared using a Wilcoxon rank-sum test. Screening data for baseline assessments and tests are presented in Supplementary Information 12 as a descriptive snapshot of the patients’ cognitive, motor and psychiatric profile at enrolment. All PD patients were tested ‘on’ their dopaminergic medication.

The EEG procedure is identical to that described for Study 1 for data collection, pre-processing and ERP extraction. We used the two different caps in a randomized order on different sessions, in order to make sure there was no effect related to one specific EEG cap. Playlists, pseudo-randomized with a Poisson distribution, were built and counterbalanced across study visits for the two arms of the study. Once the ERPs were extracted, we selected for each participant and each relevant channel the peak amplitude for the standard and the deviants by inspecting the waveform plot to identify the peak. The use of the amplitude difference at the peak, rather than the sum of the wave differences across an interval used in Study 1, was done to maximize precision in taking into account inter-individual variability. In addition, each participant acted as their own control, thus we used a Wilcoxon rank-sum test to compare the MMN amplitude between drug and placebo. We focused on each participant's best frontal electrode, where the signal was the clearest (F8 for 3 participants, F7 and F1 for the remaining two). We also ran a fixed effect analysis. For this analysis, we used the three frontal electrodes that had the cleanest signal for each participant to increase statistical power, limited to electrodes that were also relevant in the previously reported analyses.

## Results

### Study 1: PD-VH versus PD-noVH vMMN

Participants did not differ in age [*F*(1,36) = 0.71, *P* = 0.41], sex (*χ*^2^ = 0.06, *P* = 0.80), disease duration [*F*(1,36) = 2.47, *P* = 0.13], SCOPA-MOTOR scores [*F*(1,36) = 0.80, *P* = 0.38], MoCA [*F*(1,36) = 0.13, *P* = 0.72] and LEDD [*F*(1,36) = 2.44, *P* = 0.13] and dopamine agonists use (*χ^2^* = 4.92, *P* = 0.09; note: 2 PD-VH did not have this information and were excluded from this analysis). Patients did not differ on clinical severity CISI-PD^54^ [*F*(1,35) = 3.47, *P* = 0.07]; however, PD-VH patients were on average characterized by greater PD severity (1–7 points  =  mild; 8–14: moderate; ≥15 severe). We report in Table 1 the CISI-PD total score and its scores of each of the sub-scales, with PD-VH saying they feel more cognitively impaired than PD-noVH, but similar to PD-noVH for motor symptoms and motor complications. See Table 1.

**Figure 2:**
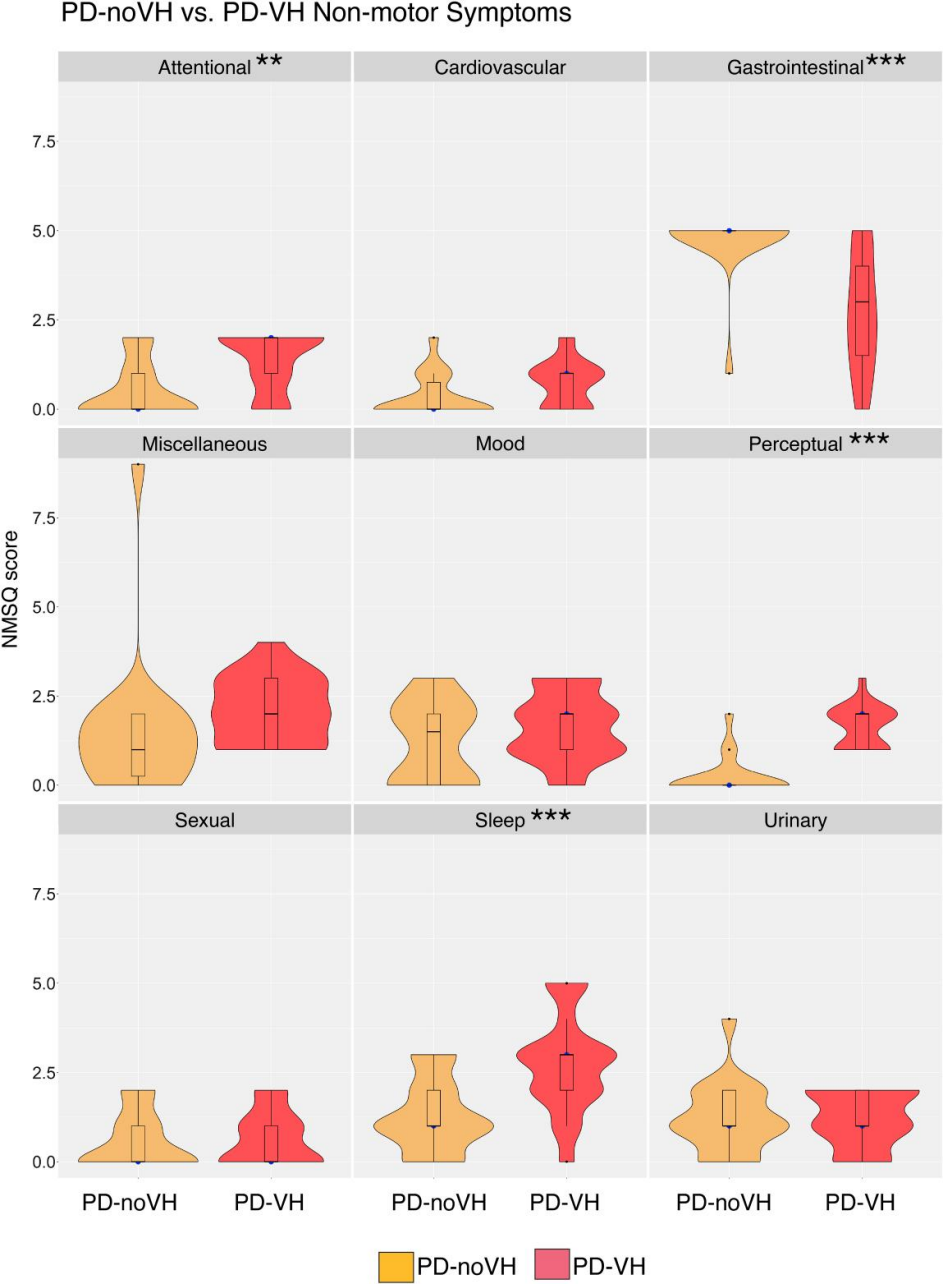
**NMSQ (non-motor symptom questionnaire) by symptom cluster**. ** *P* < 0.005, *** *P* = < 0.001 Median (blue dot) and interquartile range (black bar in the centre of each plot) are represented in the figure. We performed a one-way ANOVA with Hallucinations (Y/N) as between-subjects factor; PD-VH = 20, PD-noVH = 17; one PD-noVH had missing data for this questionnaire.

In addition to the lower CISI-PD cognitive score, PD-VH differed on non-motor symptoms (NMSQ, see Supplementary Information 1).^55^ When breaking down this score into domains, we find that PD-noVH disclose more gastrointestinal symptoms [*F*(1,35) = 21.74, *P* < 0.001] and PD-VH disclose more attentional [*F*(1,35) = 10.64, *P* = 0.002], perceptual (including VH and double vision) [*F*(1,35) = 55.72, *P* < 0.001] and sleep [*F*(1,35) = 13.43, *P* = 0.001] symptoms (see Fig. 2).

PD-VH patients were also administered PD-psychosis specific questionnaires (see Supplementary Information 1 for details) with general hallucinations score at the SAPS-PD of 10.45 ± 5.5. Out of 20 patients, only 1 presented delusions, and they retained insight. All patients presented hallucinations in the visual modality; some patients presented also other types of hallucinations (Supplementary Information), with further details from the NEVHI described in Supplementary Table 1B.

#### Within group analysis of ERP components

We conducted an analysis to compare the standard and the deviant components in each group.

For PD-noVH at the parieto-occipital channels O1, OZ, POZ, PO3, PO4, PO5, PO6 and PO8 a significant difference was found between rare deviant and standard at ∼100–125 ms, with the rare being more negative than the standard. At PO7 instead, whereas the 100–120 ms waveform has a negative peak, we found a significant difference at ∼300 ms, which was also observed at PO8 and PO6, with the rare being more positive consistently with the P300. When correcting for multiple comparisons the difference in PD-noVH remains significant for POZ in the 100–150 ms interval and at PO4 at 100 ms.

**Figure 3:**
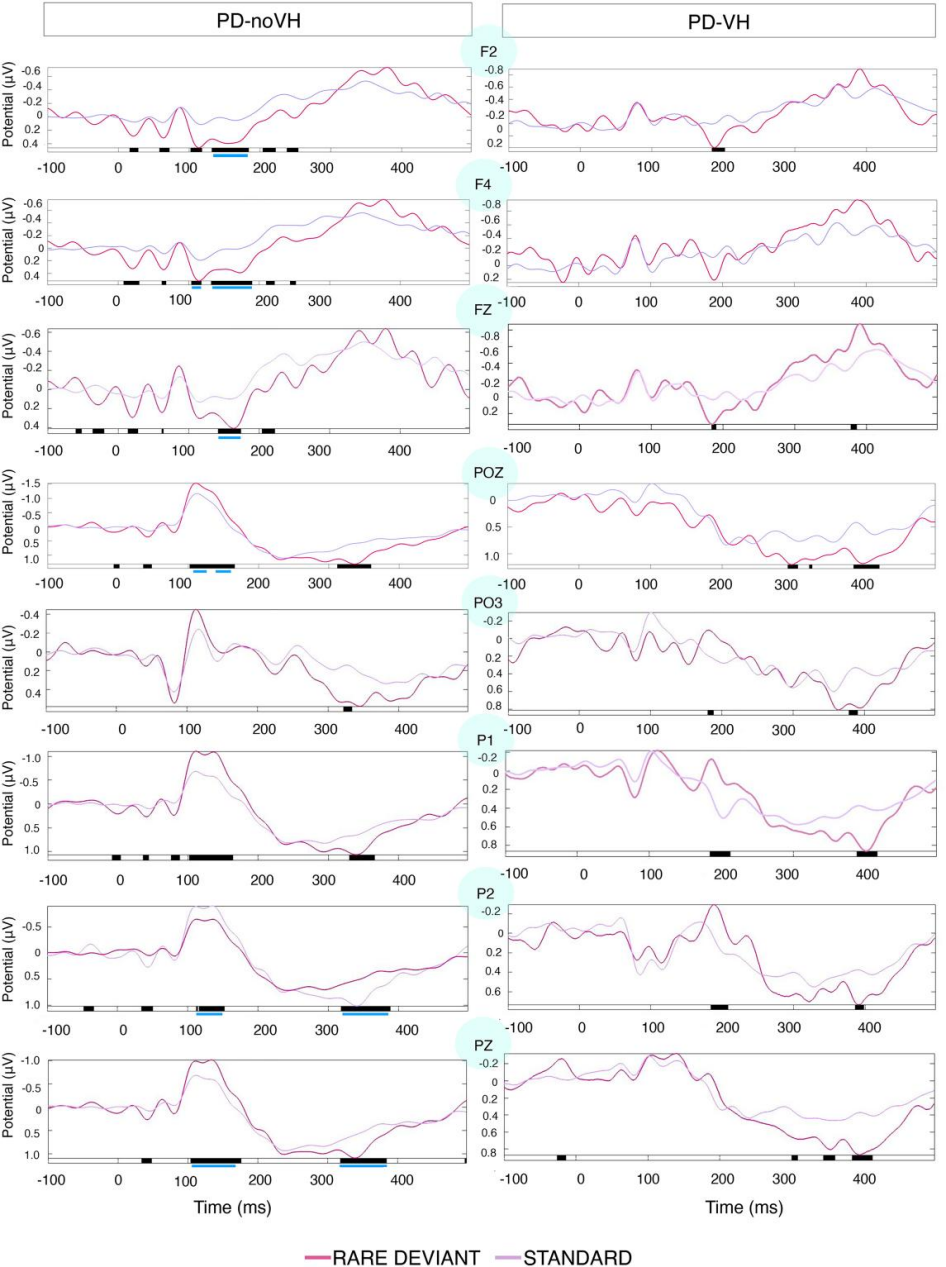
**Results of within group ANOVA comparing standard versus deviant**. Standard (violet) and deviant (pink) waveforms in PD-noVH and PD-VH in the within group ANOVA performed in EEGlab. The black bars indicate the latencies at which the two waveforms significantly differ (*P* < 0.05). The blue lines indicate the latencies at which such differences survive multiple comparisons correction (*P-*FDR < 0.05); multiple comparisons correction computed for each datapoint individually.

PD-noVH patients also had greater negativity for the deviant at PZ at 100–170 ms and greater positivity for the deviant at ∼300–400 ms, consistently with the P300 component. The negativity at 100–170 ms was significant also after FDR correction. A similar result was observed for P2 where the P300 was significant after FDR correction. At P3, P4 and P5 we also observed significantly greater negativity at 100–150 ms (FDR corrected) and greater positivity in the 300–400 ms interval, surviving FDR correction in P4 and P6 (see Fig. 3).

##### Frontal channels

For PD-noVH a significant difference was identified at central frontal (FZ), and F1, F3, F5 and F7 electrodes peaking at ∼160 ms, whereby the deviant waveform was more ‘positive’ in the PD-noVH; however, this did not survive FDR correction. At F2, we observed the same pattern over a longer latency (100–250 ms) and when FDR corrected the comparison remains significant in the 150 ms interval and a similar pattern is observed for F4. At F6 and F8, the positive difference survives FDR correction in the 170–180 ms interval. At FZ (150–220 ms) and FPZ (∼180 ms), a greater positivity and positive difference is observed, surviving FDR correction for the 140–170 ms interval.

##### PD-VH: Parietoccipital channels

At POZ, we observed a waveform consistent with the P300 at 300 ms and a similar pattern was found at PO3. Neither survived FDR correction. We observed a greater negativity at P2, however with a noisy waveform and a late (400 ms) greater positivity at P1 (not surviving FDR correction).

##### Frontal channels

A significant difference in the amplitudes with the deviant being more positive was found for FZ, F1, F2 and F3 at ∼180–190 ms (not surviving FDR correction). No significant difference between the deviant and standard waveform were present in the PD-VH group in the other frontal electrodes.

**Figure 4:**
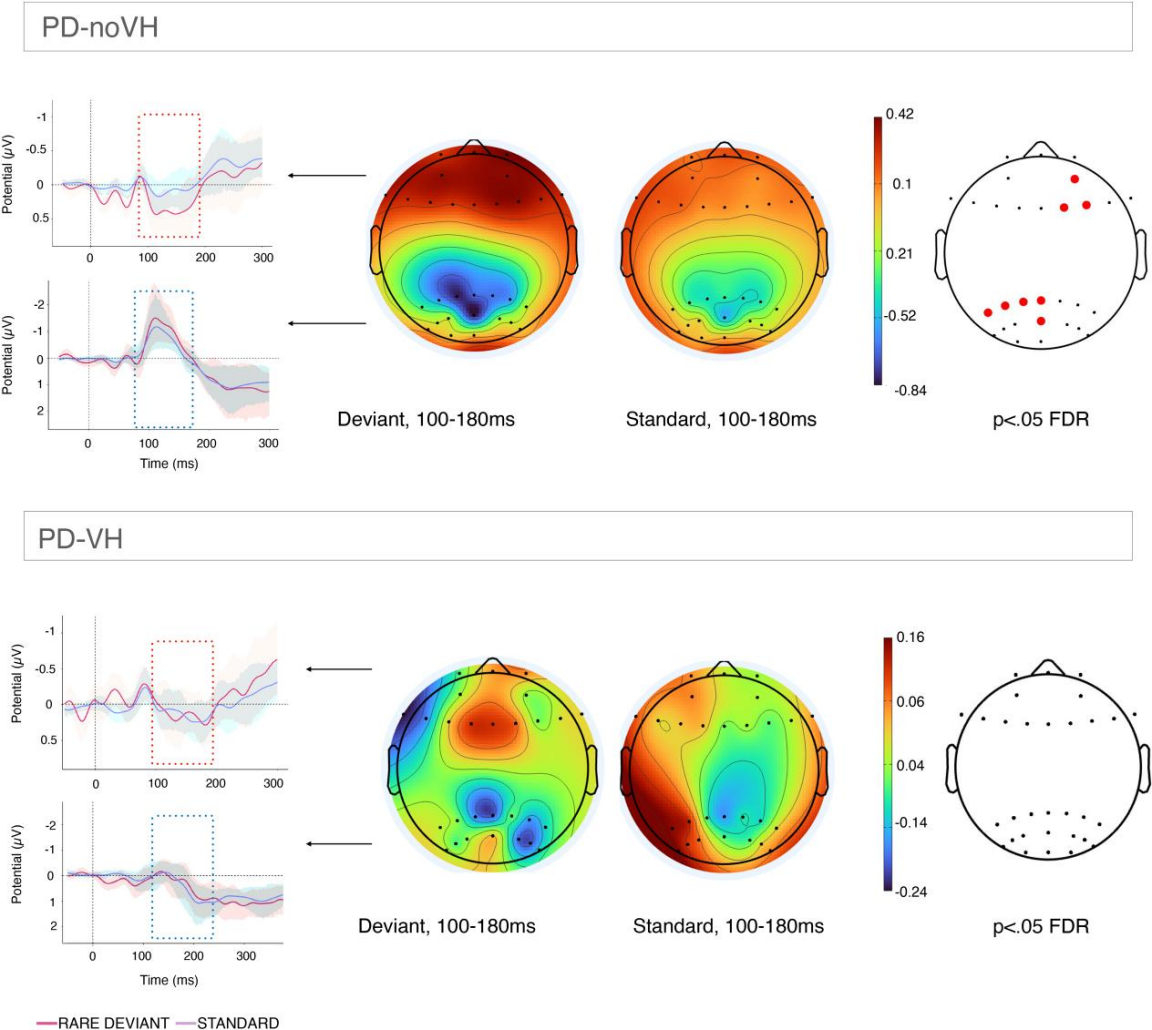
**Within group scalp topography averaged across the 100–180 ms interval**. Power spectra and event-related measures were computed for each group separately, with rare deviant/standard being entered as condition in a one-way ANOVA design. Multiple comparisons across channels were false discovery rate corrected (*P-*FDR < 0.05). When computing the analyses, a threshold was set so that only the electrodes where the rare deviant-standard difference was significant (correcting for multiple comparisons) were going to be displayed. Top row: scalp topography for PD-noVH. The channels represented with a red dot are those where a significant difference between standard and deviant (deviant was more negative posteriorly or more positive frontally) was found. Bottom row: the same analysis was used for PD-VH patients. The lateral insets show (clearly in PD-noVH) the parieto-occipital negative and frontal positive pattern we observed in the vMMN analysis (plots created with ggplot2 in R).

To better explore the difference between the standard and the deviant in each group, we analysed significant differences at the whole-brain level using scalp topography (correcting for multiple comparisons, *P-*FDR < 0.05, Fig. 4). For PD-noVH, in the 100–180 ms interval, selected to include both the earlier MMN observed at the parieto-occipital channels and the slightly delayed mismatch positivity observed frontally, we observed the pattern very clearly with the scalpogram. The whole-brain analysis of channels where the two components significantly differed at this interval revealing a right frontal (F2, F4 and AF4) and a more left parieto-occipital (POZ, PZ and P1-P5) pattern, consistent with the more focused analysis reported above. For PD-VH, the scalpogram shows a slightly more negative pattern frontally for the deviant if compared to the standard, with no significant difference across channels.

We computed the vMMN by subtracting the standard amplitude to the rare deviant amplitude in the 100–125 ms interval for parieto-occipital and 100–180 ms for frontal electrodes identified with the waveform inspection and compared PD-VH and PD-noVH with a one-way ANOVA on the channels where we observed a waveform resembling the MMN. We compared the two groups on the channels where we found a significant difference between standard and deviant that survived FDR correction in the within group analysis, in at least one of the two groups: F2, F4, F6 and F8; POZ and PO4. We explored parietal differences at both earlier and later latencies.

##### Parieto-occipital channels

**Figure 5:**
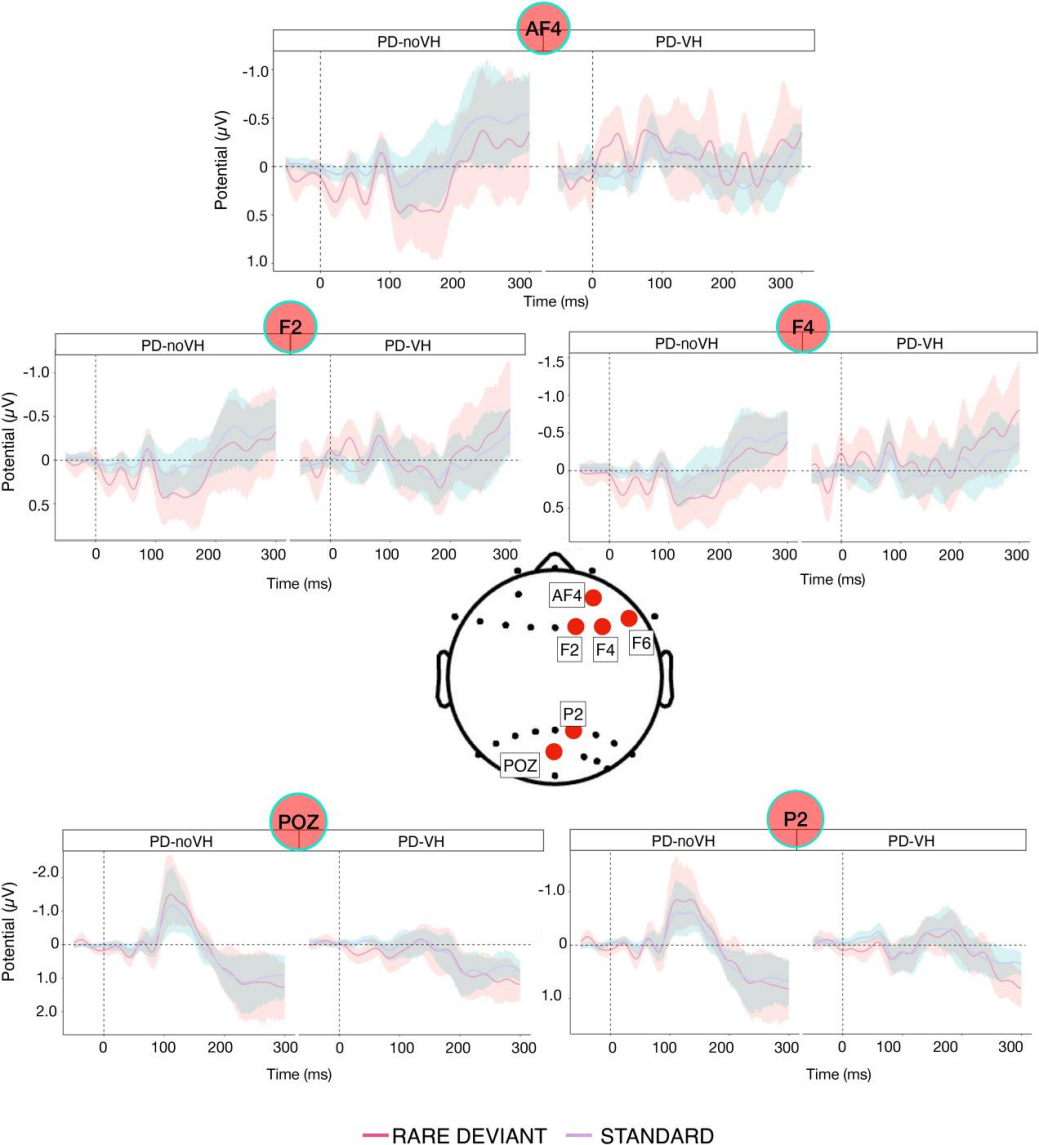
**Results of the vMMN between groups analysis**. Electrodes (red dots) that presented significant deviant-standard vMMN amplitude differences in the between-group ANOVAs (*P* < 0.05, corrected for multiple comparisons with the Benjamini–Hochberg correction). Standard (violet) and deviant (pink) waveforms for PD-VH (*N* = 20) and PD-noVH (*N* = 18). The shaded area represents the variance. vMMN amplitude was compared for the 100–180 ms interval for frontal electrodes and in the 100–125 ms for parieto-occipital electrodes.

Amplitude between groups significantly differed at POZ [*F*(1,36) = 4.32, MS = 289.821, *P* = 0.045], with PD-noVH having a mean amplitude of −4.09 (SD = 6.73) and PD-VH of. 1.51 (SD = 9.48). P2 we find a greater negativity for PD-noVH (100–180 ms interval) (mean = −6.37, SD = 1.11) > PD-VH (mean = −1.05, SD= 1.21), [*F*(1,36) = 4.19, *P* = 0.048] (see Supplementary Information 8–10 for additional details and exploratory analysis). No other electrodes at this level showed a significant difference (FDR corrected) however, a difference in the visual evoked potential waveform can be appreciated; indeed PD-noVH have a prominent posterior negative wave at 100 ms which is missing or attenuated in PD-VH (see Figs. 3–5).

##### Frontal channels

Upon visual inspection and consistently with the EEGlab analysis, at a slightly later latency (100–180 ms) than observed for MMN for the occipital and parieto-occipital channels, at the frontal channels we have observed a mismatch ‘positivity’: F2 [*F*(1,36) = 4.12, *P* = 0.05] with PD-noVH having vMMN amplitude = 9.80 (SD = 11.24) and PD-VH mean = −0.04 (SD = 17.58), F4 [*F*(1,36) = 5.85, *P* = 0.021] (PD-noVH vMMN = 9.52 (SD = 10.94) and PD-VH mean = −3.1 (SD = 19.54)), F6 [*F*(1,36) = 4.03, *P* = 0.05] (PD-noVH = 7.06 (SD = 12.32) and PD-VH = −1.14 (SD = 17.91) and AF4, [*F*(1,36) = 4.68, *P* = 0.037] (PD-noVH = 10.92, SD = 14.08, PD-VH = −2.38, SD = 22.38).

##### Parietal channels

P2 was the only parietal channel surviving FDR correction, and when comparing the MMN at the 100–180 ms latency, we find a greater negativity for PD-noVH (mean = −6.37, SD = 1.11) > PD-VH (mean = −1.05, SD = 1.21), [*F*(1,36) = 4.19, *P* = 0.048] (see Supplementary Information 8 for additional exploratory analysis). We ran a further FDR correction on these comparisons (*n* = 10), with all *P_s_*  *=* 0.05.

To better show individual differences and provide evidence for engagement with the task, we also provide individual VEPs for both groups in Supplementary Information 9 for the standard component.

We also ran an EEGlab between groups 2 × 2 design analysis showing consistent results with those presented in the main text (Supplementary Information 10).

##### Correlation with measures of hallucinations

**Figure 6:**
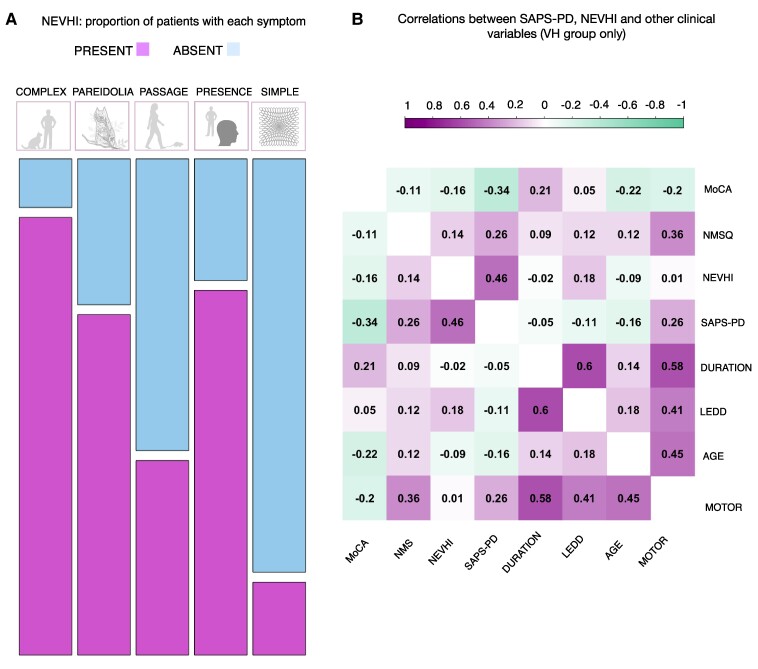
**NEVHI hallucination subtypes in the PD-VH group and clinical variables correlations**. (**A**) Proportion of patients with a specific type of VH as detected by the NEVHI visual hallucinations semi-structured interview. (**B**) Pearson's product moment correlation coefficients for the clinical variables and neuropsychiatric assessments (PD-VH only). (Motor: SCOPA-Motor score).

Neither SAPS-PD nor NEVHI correlated with disease duration, LEDD, non-motor symptoms, MoCA and SCOPA-motor scores (for details Fig. 6 and Supplementary Information 11).

**Figure 7:**
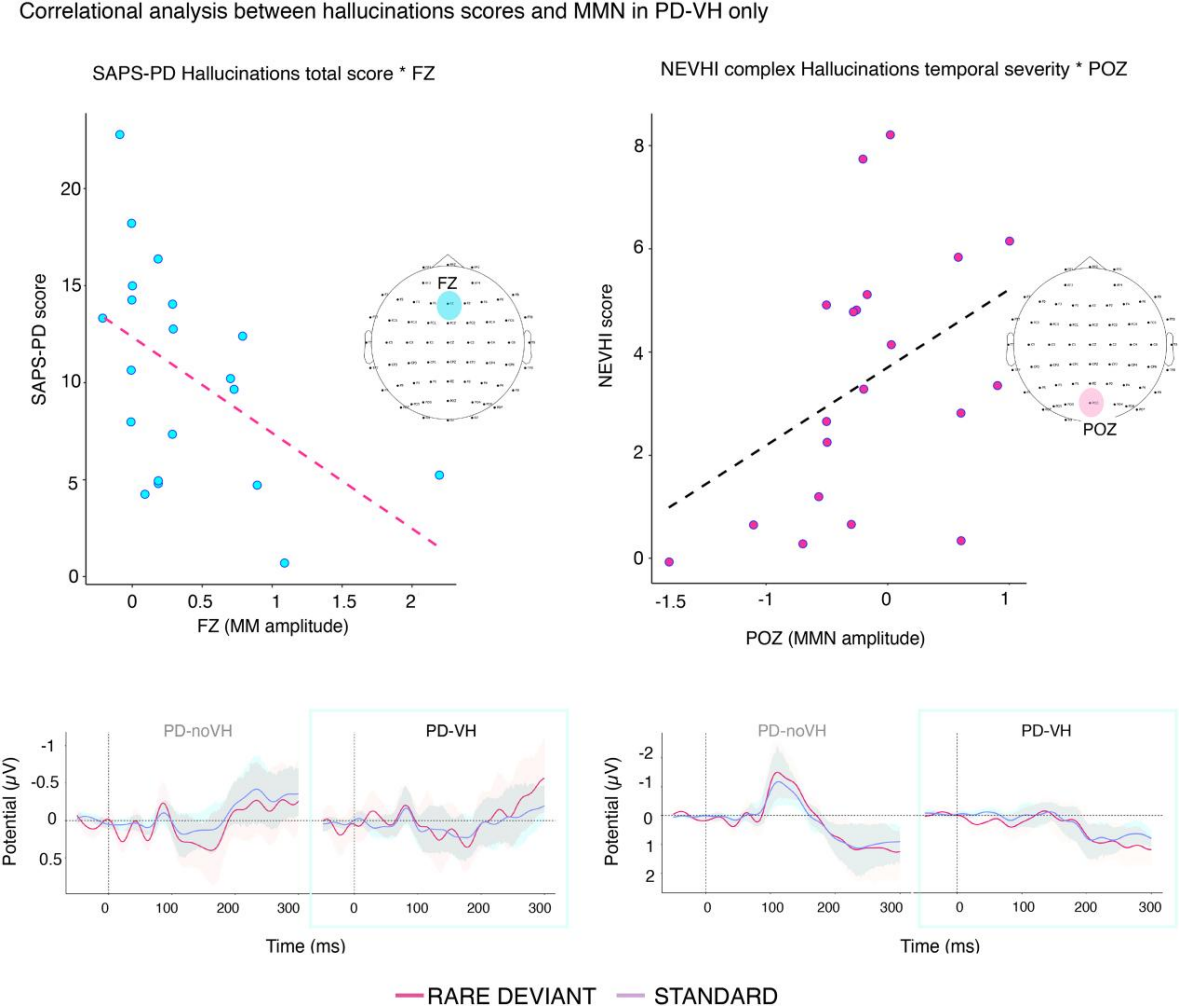
**Correlational analyses between mismatch amplitude in FZ and POZ and hallucinations severity**. Spearman correlations between hallucination severity scores and mismatch amplitude. Left: SAPS-PD scores as a function of mismatch positivity and negativity at FZ after considering the sign of the mismatch between standard and deviant (*N* = 20). Right: NEVHI temporal severity for complex VH as a function of MMN for electrode POZ. We note that in this case values that correspond to a normal [rare deviant—standard] difference are negative in the case of POZ and in this study specifically positive for FZ (for the NEVHI analysis *N* = 17 PD-VH; we focussed only on those who had complex VH).

For correlations between rare-standard mismatch and hallucinations scores, we found a significant negative correlation between SAPS-PD score and mismatch at FZ (*r* = −0.54, *P* = 0.007) with greater hallucinations scores corresponding to a smaller amplitude difference between standard and rare. We saw a positive correlation between NEVHI temporal severity for complex VH and POZ (*r* = 0.49, *P* = 0.014), with participants with a more negative MMN having lower complex VH scores. The result is the same when conducting the analysis removing the participant who said they had not had complex VH/had not had any in the past month (*r* = 0.53, *P* = 0.017) (Fig. 7).

### Study 2: Saracatinib and psilocybin behavioural study in healthy volunteers: subjective effects

The main outcome for this study was the first question on the questionnaire asking ‘How intense were the drug effects when at their peak’. Two participants did not answer this question in one session and were excluded. For the remaining participants (*n* = 18), there was a statistically significant reduction in reported intensity during the psilocybin + saracatinib session compared to psilocybin session (*t* (17) = 1.99, one-sided *P* = 0.031, *d* = 0.63; mean psilocybin = 85.4, SE = 3.5, mean psilocybin + sarcatinib = 76.4, SE = 3.6). We explored the other questions, none of which changed.

### Study 3: PD-VH only saracatinib pilot study

Five participants only completed both the drug and the placebo arm of this study and this is an important limitation. We provide the statistics for this small group as they may provide interesting input for future research targeting SRC-kinase inhibition to treat VH in PD, but we are aware that due to the very small numbers these results have to be interpreted cautiously. Each participant was used as their own control in the analysis. We report the baseline demographics and clinical data in Supplementary Information 12. All participants were males.

#### Hallucinations scores

*A* Wilcoxon signed rank test of SAPS-PD scores in the placebo and drug arms of the study showed a significant reduction of VH scores SAPS-PD [SAPS-PD_PLA_ = 9.8 ± 3.11, SAPS-PD_DRUG_ = 5.8 ± 2.17, *Z* = −2.032, *P* = 0.04]. NEVHI temporal severity scores for complex VH [NEVHI_PLA_ = 3.8 ± 1.79, NEVHI_DRUG_ = 3.4 ± 3.4, *Z* = −0.18, *P* = 0.84] were not significantly different. A similar result was found for minor VH (Supplementary Information 12).

#### Mismatch Negativity

**Figure 8:**
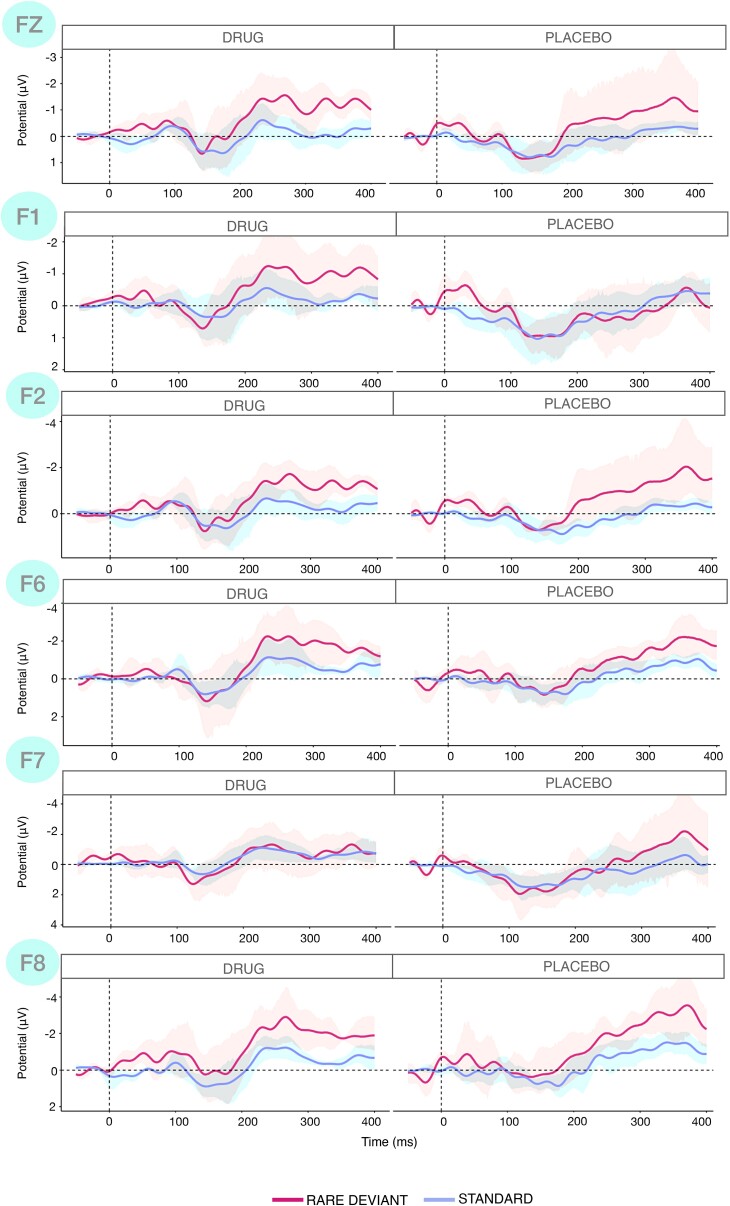
**Standard and deviant waveforms for each participant enrolled in the drug study**. Left column: frontal electrodes in the drug arm, Right column: frontal electrodes in the placebo arm. Deviant is shown in pink, standard in violet. The difference between the drug and placebo was captured in a comparison of individual peaks conducted using Wilcoxon signed ranked tests, whereby each participant acted as their own control, as described in the text; individual waveforms are displayed in Supplementary Information 10.

vMMN amplitude was shifted when participants had been on the study drug for 2 weeks. This is significant at frontal electrodes, whereby vMMN (rare-standard) amplitude is significantly more negative at ∼200–250 ms latencies (Fig. 8; Supplementary Information 10 for individual waveforms and further details). The Wilcoxon test on the best frontal electrode for each participant showed that there was a significant difference [*Z* = −2.023, *P* = 0.043] with a greater negative vMMN for the drug versus the placebo period [vMMN_PLA_ = −0.12 ± 1.09, vMMN_DRUG_ = −1.66 ± 0.69]. At the fixed effects analysis, we found that the vMMN was significantly greater at the drug versus the placebo arm of the study [*Z* = −3.11, *P* = 0.002, vMMN_PLA_ = −0.14 ± 0.65, vMMN_DRUG_ = −1.12 ± 0.83], and the rare being more negative during the drug arm [rare_PLA_ = −0.83 ± 1.35, rare_DRUG_ = −2.23 ± 1.23].

No significant differences were found at parieto-occipital channels (Supplementary Information 12).

## Discussion

We have presented clear differences in vMMN between patients with Parkinson's and Parkinson's psychosis, focusing on electrodes typically associated with the aMMN and vMMN, and experimentally validated and piloted a potential mechanism to reduce impairments. When analysing differences between the deviant and the standard ERP components we found that PD-noVH have a clear vMMN (Figs. 3–5), consistent with the aMMN in PD,^18,56,57^ whereas for PD-VH the MMN seems to be absent or very small at some electrodes. The presence of the P300, usually but not always found in auditory MMN studies, indicates that patients’ attention was to some extent responding to the deviant stimuli. In particular, we find the P3b in both groups, even if it survives multiple comparisons correction only in PD-noVH. The P3b, here evident at parietal sites, has been interpreted as an index of subsequent memory processing.^58,59^ However, we note that the typical MMN elicited frontally by aMMN is here parieto-occipital and frontally we have a positive mismatch, thus the P3b found posteriorly here might as well reflect processes usually associated with the P3a. When comparing the groups, PD-VH had reduced vMMN amplitude at the parieto-occipital and frontal channels (Figs. 3 and 5). Any laterality effects are unlikely to be due to laterality of symptoms as patients did not differ in this. Reduced MMN amplitude is one of the most consistent and replicated results in psychosis^9,10,12,13^ and thus, it is consistent with our initial hypothesis that these patients would respond differently to the sensory environment presented with our vMMN task. A recent genome-wide association study found MMN to be a superior psychosis marker if compared to others previously associated with psychosis risk, such as lateral ventricular volume and verbal learning.^60^ Our results confirm the sensitivity of this measure when comparing patients with and without hallucinations and related perceptual phenomena within the same diagnostic group.

As there is no literature about MMN in PD-VH, we can speculate that the reduced MMN reflects the greater discrepancy between the sensory input and top–down predictions in PD-VH; whereby, the brain is impaired in its ability to resolve such a discrepancy, as proposed by the predictive coding account of psychosis symptoms.^61^ A more in-depth analysis of models of VH in PD has been recently carried out to create a consensus framework for VH in Parkinson's.^62^ Within this framework, the authors reconcile different models of VH, considering ‘ascending’ sensory disturbances (i.e. perceptual deficits and environmental factors), ‘descending’ factors (i.e. overly influent and biasing expectancies, semantic knowledge and memory traces, attentional deficits at spatial, object and orientation level). In this view, defective visual input and biasing (prefrontal) signals concur to the generation of VH, with poor frontal attentional (redirection) and control mechanisms and a potentially impaired frontotemporal semantic processing also playing a role (for an extensive discussion^62^). Our results are also congruent with previous neuroimaging studies of PD-VH in different modalities.^17,21,62-64^

Concerning the finding of differences in frontal and visual regions in our analyses, we hypothesize that the frontal and occipital electrodes are picking up local sources, rather than a dipole halfway between them. The later frontal mismatch we find (100–180 ms) if compared to parieto-occipital channels (100–125 ms) could be explained in terms of hierarchy in the cortex. We know that ‘higher’ brain regions respond to incoming information changing over longer intervals.^65^ Ascending connections from lower areas, such as the primary visual cortex, project to frontal areas arising from cortical layer III and target layer IV. It has thus been proposed that connections between brain regions at different hierarchical levels may represent the neural correlates of the probabilities mapping hidden causes (higher regions) to the sensory input (lower regions).^61^ In this case, ascending connections between primary visual and frontal regions would carry prediction error signals. In a neurodegenerative condition such as PD, we also need to consider that it can be characterized by widespread grey matter loss, especially in progressive subtypes, and such a reduction would correspond to a reduction in the models that the activity patterns across the neurons of a region can represent, thus limiting their accuracy.^66^ In addition, dopamine has been proposed to be linked to precision of prior beliefs, and it has been suggested to play a role in movement initiation deficits in PD^66^ and in the optimisation of sequences of actions.^67^ It is thus possible that dopamine depletion plays a role as well, but we know from previous research^17^ that dopaminergic deficits alone cannot account for VH and indeed a role of cholinergic^62,68^ and serotonergic^24^ dysfunction has been proposed.

In the current study, we have tested the potential role of the serotonergic 5-HT_2A_ cascade in VH in PD with hallucinations. The receptor trafficking hypothesis was first experimentally tested in healthy volunteers by pre-dosing a psilocybin administration with saracatinib, providing support for the role of the Gi/o-dependent pathway in the psychedelic effect. The study was not designed to test for a selective effect on different visual experiences (e.g. pareidolia versus complex VH) but we found a general reduction in the intensity of experience. A longer duration of dosing in a small sample of patients also supported the hypothesis of the involvement of the Gi/o-dependent pathway in PD-VH. Changes in the 5-HT_2A_ signalling pathway following SRC-kinase inhibition appear to modulate the vMMN at frontal channels (Supplementary Information 12), but not at occipital sites. 5-HT_2A_ receptors are widespread in the cerebral cortex, particularly in frontal and occipital areas^69^ and 5-HT_2A_ receptors’ density is affected in PD^25,70^ and in schizophrenia.^71^ In addition, serotonin and glutamate ligands have been found to tune the pattern of G-protein coupling in the 5-HT_2A_/mGlu_2_R complex linked to the pathophysiology of schizophrenia,^30^ suggesting 5-HT_2A_ and mGlu_2_R as potential targets for antipsychotic drug development, which our previous mechanistic work has supported,^72^ although trials are yet to be successful in patients.^73^ Our results in PD-VH, together with previous findings linking 5-HT_2A_ receptors density to differences in cortical thickness between PD-noVH and PD-VH patients^21^ adds further to the potential role of serotonergic modulation of VH and related symptoms in Parkinson's disease. In our small sample, we observed a reduction in global rating of hallucinations as measured by the SAPS-PD and altered vMMN. We can only speculate why the effect of SRC-kinase inhibition via saracatinib appears to modulate the vMMN only at frontal rather than occipital sites. A possibility is that damage between frontal and occipital areas in the serotonergic pathway prevents the drug from acting evenly throughout the 5-HT_2A_ pathway. Another possibility is that it might take longer than 14 days for the drug to be fully effective.

Another interesting result is the different sign of the MMN we observed at different cortical sites. We found visual MMN at parieto-occipital sites and a ‘positivity’ at frontal channels (Fig. 3). Other studies have reported visual mismatch positivity at central^12,14^ and frontal^74^ sites, where we also found longer latencies at frontal electrodes, consistent with previous research.^75^ Among the possible explanations for this is that, as a dual generator is proposed for auditory MMN (for an in-depth review^36^), with a temporal and a frontal generator, this could also be the case for visual MMN, with a parieto-occipital and a frontal generator or components. However, it is unclear why the polarity of the MMN activity is different in each region and, to confirm this suggestion, detailed source localisation analysis would be important. We can speculate that these parieto-occipital and frontal components may be part of a network of hierarchical cortical sources^2^ with these regions being in turn responsible for the comparison of the standard and deviant and the subsequent direction of attention, as previously found at the temporal and frontal level for aMMN.^76^ Indeed, the P300 component is thought to signal the full attention switch^77^ but is not always there; in our study, we found it at some, but not all, parietal and parieto-occipital in PD-noVH only (Fig. 2) (a negative N300 component was also observed at frontal electrodes but was rarely significant).

We also explored the relationship between different aspects of VH intensity and vMMN amplitude, finding a correlation between SAPS-PD and NEVHI (temporal severity of complex VH) scores and (deviant—standard) amplitude at FZ and POZ, whereby the more severe the symptoms, the smaller the difference between the two waveforms.

### Limitations

The study has some limitations. First, we could not enrol the original target number for each group for Study 3, thus limiting the power of our analyses. The study started shortly before the COVID-19 pandemic and was severely impacted by both lockdowns. As the safety of patients who were particularly at risk was the primary concern, recruitment slowed significantly. This impacted more heavily on the drug study, for which only five participants completed both arms before the drug company stopped manufacturing the study drug, which is a second limitation. Second, unfortunately, due to the challenges in recruitment over the pandemic, we did not include sufficient participants to have different blocks of patients administered different versions of the task featuring a different orientation for standard and deviant stimuli.

## Conclusions

MMN is sensitive to PD with hallucinations, and we also proposed and tested a mechanism through which PD MMN impairments may be reduced in PD psychosis, validated through a psychedelic model of altered visual experience.

We demonstrate that MMN is a sensitive and potentially useful tool in experimental testing of mechanisms to reduce symptoms in PD psychosis with an exemplar pilot case series with positive results.

## Supplementary Material

fcae291_Supplementary_Data

## Data Availability

Summary data as EEG pre-processed amplitude for standard and deviant components (time range for each epoch from −100 to 500 ms) is made available in a dedicated Github repository; clinical anonymized data (SCOPA-MOTOR, LEDD, disease duration, age, sex, SAPS-PD, NEVHI-complex, NPI and NMSQ) is also made available; peak dataset for correlations between POZ and FZ mismatch and NEVHI and SAPS-PD in the VH group are made available). Raw EEG.CNT files are not at the moment available as these are currently being processed for further analyses. The data can be made available upon reasonable request (e.g. for collaborations). The code generated for the waveform plots and the vMMN ANOVAs in R is made available (GitHub repository link: https://github.com/VMiri/PDP_MMN).
