# Supplementary material for: Visual mismatch negativity in Parkinson's psychosis and potential for testing treatment mechanisms
Source: Brain Commun. 2024 Sep 3;6(5):fcae291. doi: 10.1093/braincomms/fcae291 (PMC11443450; doi:10.1093/braincomms/fcae291)
Supplement: fcae291_Supplementary_Data [file fcae291_supplementary_data.zip › Original_submission_manuscript.pdf]

OXFORD UNIVERSITY PRESS | Brain Communications

Visual mismatch negativity in Parkinson’s psychosis and potential for testing treatment mechanisms

Journal:	*Brain Communications*
Manuscript ID	BRAINCOM-2023-567
Manuscript Type:	Original Article
Date Submitted by the Author:	05-Dec-2023
Complete List of Authors:	Vignando, Miriam; King's College London Institute of Psychiatry Psychology & Neuroscience, Neuroimaging<br>ffytche, Dominic; King's College London, Institute of Psychiatry, Psychology & Neuroscience<br>mazibuko, Ndabezinhle; King's College London Institute of Psychiatry Psychology & Neuroscience, Neuroimaging<br>Palma, Giulio; University of Southampton, Department of Psychology<br>Montagnese, Marcella; University of Cambridge, 4. Department of Clinical Neurosciences<br>Dave, Sonali; City University of London<br>Nutt, David J.; Imperial College, London, UK, Dep. of Neuropsychopharmacology<br>Gabay, Anthony; IXICO plc<br>Tai, Yen; Imperial College London, Faculty of Medicine, Department of Brain Sciences<br>Batzu, Lucia; King's College London; King's College Hospital<br>Leta, Valentina; Istituto Nazionale Neurologico Carlo Besta, Department of Clinical Neurosciences, Parkinson and Movement Disorders Unit<br>Williams-Gray, Caroline; University of Cambridge, Brain Repair Centre, Department of Clinical Neurosciences<br>Chaudhuri, K R; King's College Hospital NHS Foundation Trust, Parkinson's Centre of Excellence, Department of Neurology<br>Mehta, Mitul; 1. Centre for Neuroimaging Sciences, Institute of Psychiatry, Psychology and Neuroscience, King’s College London, De Crespigny Park, SE5 8AF , Neuroimaging
Keywords:	

SCHOLARONE™ Manuscripts

Visual mismatch negativity in Parkinson's psychosis and potential for testing treatment mechanisms

Miriam Vignando[1], Dominic ffytche[2], Ndabezinhle Mazibuko[1], Giulio Palma[3], Marcella Montagnese[4], Sonali Dave[5], David Nutt[6], Anthony S. Gabay[7], Yen F. Tai[8], Lucia Batzu[9], Valentina Leta[9,10], Caroline H. Williams Gray[11], K. Ray Chaudhuri[9] & Mitul A. Mehta[1]

1. Centre for Neuroimaging Sciences, Institute of Psychiatry, Psychology and Neuroscience, King's College London, De Crespigny Park, SE5 8AF
2. Institute of Psychiatry, Psychology and Neuroscience, King's College London, De Crespigny Park, SE5 8AF
3. Department of Psychology, University of Southampton, UK
4. Department of Clinical Neurosciences, Herchel Smith Building, University of Cambridge, UK
5. City, University of London, Northampton Square, EC1V 0HB, London
6. Imperial College London, Faculty of Medicine, Department of Brain Sciences, Burlington Danes, The Hammersmith Hospital, Du Cane Road, London W12 0NN
7. IXICO, 4th floor, Griffin court, 15 Long Ln, Barbican, London EC1A 9PN
8. Imperial College London, Faculty of Medicine, Department of Brain Sciences, Charing Cross Hospital, Fulham Palace Road, London W6 8RF
9. Parkinson Foundation Centre of Excellence, King's College Hospital NHS Foundation Trust, 245A Coldharbour Lane, SW9 8R, London
10. Fondazione IRCCS Istituto Neurologico Carlo Besta, Department of Clinical Neurosciences, Parkinson and Movement Disorders Unit, Milan, Italy.
11. John Van Geest Centre for Brain Repair, Department of Clinical Neurosciences, University of Cambridge/Cambridge University Hospitals NHS Foundation Trust

For Review Only

Abstract

Psychosis and visual hallucinations are a prevalent non-motor symptom of Parkinson's disease (PD), negatively affecting patients' quality of life and constituting a greater risk for dementia. Understanding neural mechanisms associated to these symptoms is instrumental for treatment development.

The mismatch negativity (MMN) is an event-related potential evoked by a violation in a sequence of sensory events. It is widely considered an index of sensory change-detection. Reduced mismatch negativity response is one of the most replicated results in schizophrenia and has been suggested to be a superior psychosis marker.

To understand whether this ERP component could be a similarly robust marker for PD psychosis, we used electroencephalography with a change-detection task to study the MMN in the visual modality (vMMN) in 20 patients with PD and visual hallucinations and 18 matched PD without hallucinations. We find that vMMN is clearly present in patients with PD without hallucinations at both parieto-occipital and frontal sites, whereas PD-VH show reduced or no differences in the two waveforms, confirming the sensitivity of MMN to psychosis, even within the same diagnostic group. We also explored the

relationship between VH severity and vMMN amplitude, finding a negative correlation between visual hallucinations severity scores and vMMN amplitude at a central frontal (FZ) and parieto-occipital (POZ) electrodes, whereby the more severe or complex (illusions, formed VH) the symptoms the smaller the amplitude.

We have also tested the potential role of the serotonergic 5-HT_{2A} cascade in VH in PD with hallucinations, following the receptor trafficking hypothesis. We did so with a pilot study in healthy controls (N=18) providing support for the role of the Gi/o-dependent pathway in the psychedelic effect and a case series in PD-VH (N=5) using a double-blind crossover design. Positive results on psychosis scores and MMN amplitude add further to the potential role of serotonergic modulation of visual hallucinations in Parkinson's disease.

Keywords: mismatch negativity; Parkinson's disease; Parkinson's psychosis; 5-HT_{2A}; visual hallucinations

Abbreviations: mismatch negativity (MMN); Parkinson's disease (PD); Parkinson's disease psychosis (PDP); non-motor symptom scale (NMSS); clincal index of severity impression (CISI); visual hallucinations (VH); the Scale for the Assessment of Positive Symptoms-PD (SAPS-PD); North-East Visual Hallucinations Interview (NEVHI)

For Review Only

Introduction

The mismatch negativity (MMN), an event-related potential evoked when there is a violation in a sequence of sensory events, is widely considered an index of sensory change-detection[1], and as a perceptual prediction error[2]. Despite the wealth of research on MMN, the biological underpinnings of the MMN are not completely understood, but several hypotheses have been presented. The most influential one is predictive coding[3-5] – a computational mechanism whereby event-related responses are proposed to be an expression of the brain attempting to minimize prediction errors. Within this framework, the MMN is considered an index of the neural activity reporting such a prediction. Using hierarchical Bayesian inference the brain is proposed to work out the causality of sensory inputs by inverting the process that generated such inputs from the environment and resolve discrepancies such as that produced by the MMN. This view has been used to explain the disruption of MMN in psychosis[6-8]. Indeed, a reduced auditory MMN response is one of the most replicated results in schizophrenia[9,10]. Initially, the MMN was thought to be present only for the auditory modality, but it has been demonstrated for the somatosensory[11] and visual modalities, with the visual MMN (vMMN) also reduced in schizophrenia[12-14].

Psychosis also occurs in Parkinson's disease (PDP) as one of the most common non-motor aspects[15,16]. Psychosis in Parkinson's follows a behavioural continuum, with minor hallucinations (including illusions) presenting first, followed by fully formed visual hallucinations (VH)[17]. Studies of MMN in Parkinson's without psychosis have been limited to the auditory domain and have shown a normal MMN amplitude in patients at the early stages of the disease[18]. As PD progresses, a smaller amplitude can be observed[19]. To the best of our knowledge, there are no reports of the MMN in patients with PDP in the auditory or visual domain. Since hallucinations in PDP are visual, we suggest vMMN is a more appropriate test within the framework of predictive processing[20].

Functional neuroimaging studies investigating VH show widespread alterations in brain function, including dysfunction of the ventral visual pathway[16]. Structural imaging studies also implicate brain regions outside of visual processing areas, including parietal, frontal, hippocampal and cerebellar regions[17,21] together with the involvement of regions of the attentional control networks (i.e. the default mode and the dorsal and ventral attentional networks)[21,22]. Together, these studies fit with models of PDP proposing both disrupted bottom-up processing of sensory information and dysfunctional top-down influences on perceptions[22]. A recent fMRI DCM study of patients with PDP showed the differences between patients with and without VH were best explained when taking into account diminished visual (bottom-up) and dysfunctional top-down (PFC to thalamus) processes, adding further to the predictive coding hypothesis - proposing a strong role of incorrect priors from top-down, prefrontal, areas[23]. This supports our hypothesis that that vMMN might help identify specific neural mechanisms associated with visual hallucinations in PDP.

Analyses using receptor density maps suggest that grey matter loss in PDP is linked to the 5-$HT2_A$ receptor distribution[21]. Two studies have directly tested striatal and extra-striatal serotonin markers in PDP. Direct measurement of 5-$HT2_A$ receptor availability has produced mixed findings with two studies showing an increase in binding in the ventral visual pathway, and one showing a decrease in binding. The increased binding was shown in one *in vivo* PET study using [18F]-setoperone[24] and a post-mortem study using [3H]-ketanserin[25]. The decrease in binding was in a group of PDP with cognitive deficits showing a decrease in [18F]-setoperone binding, which correlated with cognitive function[26].

Several lines of evidence have linked 5-HT_{2A} receptors to visual processing and hallucinations. First, 5-HT_{2A} receptors are highly expressed in the visual cortex layers I, IV and V[27] and altered 5-$HT2_A$ activity in layer V modulates alpha wave activity[28], involved in both visual processing and visual hallucinations. Second, 5-HT_{2A} receptor agonism with psychedelics produces altered visual experiences, which can include hallucinations[28,29] and these can be blocked with ketanserin, a 5-HT_{2A} receptor antagonist. Ketanserin also reduces N170 VEPs that are associated with the visual perceptual alterations produced by psilocybin[28]. The receptor trafficking hypothesis provides a reason why only some 5-HT_{2A} agonists lead to altered visual experiences. The specific proposal is that psychedelic 5-HT_{2A} agonists act through the 5-$HT_{2A}R/mGlu_2R$ heterocomplex and activate Gi/o-dependent signaling as well as Gq/11[30,31]. We reasoned that by targeting the Gi/o-dependent pathway (through SRC-kinase inhibition)

we could reduce the psychedelic effects of psilocybin in healthy volunteers, conferring the potential to reduce VH in PD. In rodents SRC-kinase inhibition blocked 5-HT_{2A} receptor activation induced head twitches associated with a 'hallucinogenic' response[31]. In this paper, we present the results of a study conducted in healthy volunteers to test if the SRC-kinase inhibitor *saracatinib* attenuates the psychedelic experience in humans, to translate the findings from experimental animals[32] before conducting a pilot study in patients with PD-VH.

Overall, we hypothesise vMMN will be affected in PDP/PD-VH, similar to what is observed in other psychoses[20]. Thus, we expect to find an alteration of the vMMN in patients with hallucinations when compared to patients without hallucinations. We expect this to be present at different EEG channels. Studies using auditory stimuli have led to propose separate temporal dynamics of aMMN production, with a frontal and a temporal component[33,34]. The two components have also been proposed to reflect the MMN arising from the comparison of sensory inputs and top-down predictions that rely on a memory trace.[35,36]

Thus, we also hypothesise a similar pattern for vMMN with a frontal and a parieto-occipital components. We also hypothesise that *saracatinib* would reduce behavioural measures of the psychedelic experience. Finally, we test the hypothesis that *saracatinib* can modulate vMMN in PD with hallucinations via SRC-kinase inhibition. We also test the hypothesis that this modulation of vMMN is accompanied by a reduction of visual hallucinations.

For Review Only

Materials and Methods

For all three studies, approval was received by the R&D office of KCL and the NHS Trusts. Ethics approval was granted by the KCL Research Ethics Committee for the healthy volunteer study (PNM/14/15-11) and the HRA ethics committee for the studies in patients (18/LO/2144). Written informed consent was obtained prior commencing study procedures following the Declaration of Helsinki.

Study 1: PD-VH vs. PD-noVH vMMN EEG study

Patients with Parkinson's disease without (PD-noVH) and with visual hallucinations (PD-VH) took part in this study. Participants were recruited through the Parkinson's UK research hub, the Movement Disorder clinics at the Parkinson's center of excellence at King's College Hospital (KRC), Charing Cross Hospital (YT), Cambridge University Hospitals NHS Trust (CWG), and from a previous KCL study[37]. We enrolled 19 PD-noVH and 21 PD-VH, but one participant from each group did not have usable data from one of the main electrodes (POZ). The final sample comprised 18 PD-noVH and 20 PD-VH.

After a phone pre-screening, participants attended one study day at the clinical research facility (CRF) of King's College Hospital where they completed screening, for which we collected medical history, medication, physical and non-motor symptoms examinations (see **SI1** for a complete description and **Table1**), cognitive testing and conducted the EEG session.

To investigate visual hallucinations, participants' partners completed the hallucinations and delusions scale of the Neuropsychiatric Inventory[38], and PD-VH participants were administered the Scale for the Assessment of Positive Symptoms-PD (SAPS-PD) adapted for PD from schizophrenia[39] and an adaptation of the North-East Visual Hallucinations Interview (NEVHI), a semi-structured interview to assess the phenomenology of visual hallucinations[40]. Since there are no specific scoring rules for the adapted version of the NEVHI, we computed temporal severity by multiplying duration and the frequency of the VH, using an ordinal scale. We also computed a continuous temporal severity score multiplying the raw number of minutes spent hallucinating by the raw number of VH in a month (see **SI1**). Study day assessments and descriptions of the questionnaires are reported in **SI1** and **Table1**.

The vMMN task *(Presentation v.17.2)* required participants to decide via button press when the fixation cross at the center of the screen changed in size, while ignoring peripheral stimuli. The task was developed from one of the paradigms presented in Qian et al.[41] and is described in detail together with the EEG acquisition protocol in **SI 2-5** (see **Figure1** for a visual summary).

- **Figure 1** -

Data analysis

Descriptive statistics for sociodemographic and clinical information are reported in **Table 1**.
Thirteen out of 38 participants had a phone screening during the pandemic (instead of in-person). For this we administered the blind MoCA and converted the score to the full MoCA[42] (Melikyan et al., 2021). On the study day the full MoCA was also administered to confirm eligibility (MoCA >22), but the initial score was retained for analysis to minimise the influence of practice effects.
For one of the participants, it was not possible to administer the SCOPA-motor. For this participant we had a recent full UPDRS (< 2 weeks), and the score was converted using a method described previously[43].

For Review Only

Data were pre-processed with EEGLAB v2021.1[44] and ERPLAB v8.3.0[45] (Matlab version R2022b, Mathworks, Inc.) using the standard EEGlab preprocessing pipeline. A detailed description is reported in **Supplementary Information 4**.

Statistical data analysis was carried out in R version 4.0.3[46], using the following packages: dplyr[47], ggcorrplot[48], PairedData[49], ICcmodavg[50], tidyverse[51], ggpubr[52] and broom[53], and SPSS Version 28.0. Armonk, NY: IBM Corp.

First we carried out an across-participants analysis of standard and rare deviant ERP components was performed using a script within EEGlab's STUDY structure[45]. Epoched data was subject to a bin-based sub-setting in 'rare deviant' and 'standard' datasets for each participant. These datasets were entered in the model, with rare deviant/standard being entered as condition. A set of power spectra and event-related measures for each dataset were computed, with rare deviant/standard being entered as condition and PD-VH/PD-noVH as group in a one-way ANOVA design. Each dataset entered in the model is, with this step, associated with an ICA decomposition and thus a series of components. Each component has an equivalent dipole model, based on which pairwise distance measures between components can be then computed. Multiple comparisons were false discovery rate corrected ($p < 0.05$). The same procedure was used for the between-group analysis conducted with both PD-VH and PD-noVH whereby standard/deviant was entered as condition and PD-VH/PD-noVH as group in a one-way ANOVA design.

For Review Only

ERP component analysis and vMMN measurements. The auditory MMN (aMMN) is traditionally expressed at fronto-central electrodes, typically at 100 - 250 milliseconds after stimulus onset. However, results vary across studies when latency is considered, with some reporting a MMN as early as 90ms and others as late as 400ms[20]. Here we observed an earlier (100-125ms) MMN at parieto-occipital sites and a more extended (100-180ms) MMN at frontal sites. Difference waves between the standard and individual deviant conditions were generated for each participant for the time interval considered. The difference waves were then summed to obtain the difference wave in the time interval of the MMN. The interval was defined by plotting the grand average standard and rare deviant waveforms and identifying the vMMN. In addition, individual waveforms were visually inspected to make sure that the interval selected took into account inter-individual variability. We also used the EEGlab study analyses to confirm the time interval selected for the vMMN amplitude analysis.

Once the average vMMN amplitude was estimated for each participant we compared PD and PD-VH with ANOVAs in R. First, we checked the distribution of the data at each channel of interest. For this analysis, we focused on the electrodes where the waveform presented the typical features of an MMN, thus the negative peak followed by a P300 component. We analysed data from specific parieto-occipital and frontal electrodes, based on the results from the EEGlab study.

For the correlational analyses between hallucinations measures and MMN we ran parametric or non-parametric tests as appropriate. We first checked whether SAPS-PD or NEVHI scores correlated with any of the other clinical variables (levodopa equivalent dose, disease duration, SCOPA-MOTOR, age, MoCA, non-motor symptoms). To test the relationship between the severity of VH (SAPS-PD and NEVHI) and MMN, we only analysed the PD-VH group. For the NEVHI, we focussed on complex VH. and ran the analysis both in all 20 participants and in the 17 who presented complex VH. We used one-tailed correlations as our hypotheses were that people with more severe hallucinations would have a more altered (reduced) mismatch.

We focussed on electrodes where we found a difference between the groups. For frontal electrodes, since some but not all participants had a positive mismatch wave, we used a signed MMN index (zero when the deviant and standard were the same). For POZ we kept the raw scores as no participants had a positive mismatch wave at parieto-occipital electrodes. To make sure that what we were measuring was indeed (rare deviant – standard), we used individual peaks rather than the sum of amplitudes over a specific interval. This allowed us to accommodate inter-individual differences in this VH-only sample.

Task data analysis. Task data analysis was carried out to validate the task (pilot analysis in Supplementary information **S1**) and to analyse participants' performance at the cross-change detection task. PD-noVH patients performed better than PD-VH at the distractor task (**Supplementary Information 3** for details).

Study 2: Saractinib and psylocibin behavioural study in healthy volunteers

There were two main predictions of this study which were that a single oral dose of 125mg saracatinib would attenuate the subjective effects of 2mg psilocybin infusion and reduce neuroimaging markers. Here we present the subjective outcomes to validate the use of this drug for the patient pilot study. Detailed methods for this study are reported in **Supplementary Information 6**.

Study 3: PD-VH only saracatinib pilot study

Of 42 potential participants identified, 25 met criteria and were phone screened. Of them, 11 were eligible for the drug study and seven passed the screening and were enrolled into the study, with 5 completers. The drug study was not completed due to interruption of drug manufacturing due to reasons unrelated to the study or safety concerns (see **Supplementary information 6** for additional details and detailed

For Review Only

experimental design and procedure). Here we present the results as a pilot case series. In depth methods for this study are reported in **SI 10**.

Those enrolled were randomised to take a daily oral dose of 100mg of saracatinib every morning for 14 days (+/- 2 days) for one study period and matched placebo for the other study period within a double-blind study design. Order was unblinded after the EEG data were pre-processed and ERP data extracted. The same clinical data was collected as Study 1 for all visits. A secondary aim of the study was to assess whether any of the psychosis symptoms change with the administration of saracatinib, which were compared using a Wilcoxon rank-sum test. Screening data for baseline assessments and tests are presented in **SI 10** as a descriptive snapshot of the patients' cognitive, motor, and psychiatric profile at enrolment.

The EEG procedure is identical to that described for Study 1 for data collection, pre-processing and ERP extraction. We used the two different caps in a randomised order on different sessions, in order to make sure there was no effect related to one specific EEG cap. Playlists, pseudo-randomised with a Poisson distribution, were built and counterbalanced across study visits for the two arms of the study. Once the ERPs were extracted, we selected for each participant and each relevant channel the peak amplitude for the standard and the deviants by inspecting the waveform plot to identify the peak. The use of the amplitude difference at the peak, rather than the sum of the wave differences across an interval used in Study1, was done to maximise precision in taking into account inter-individual variability. In addition, each participant acted as their own control, thus we used a Wilcoxon rank-sum test to compare the MMN amplitude between drug and placebo. We focused on each participant's best frontal electrode, where the signal was the clearest (F8 for 3 participants, F7 and F1 for the remaining two). We also ran a fixed effects analysis. For this analysis we used the three frontal electrodes that had the cleanest signal for each participant to increase statistical power, limited to electrodes that were also relevant in the previously reported analyses.

For Review Only

Results

Study 1. PDP vs. PD-noVH vMMN.

Participants did not differ in age [$F(1,36) = 0.71$, $p = .41$], sex ($\chi 2 = .06$, p =.80), disease duration [$F(1,36) = 2.47$, $p = .13$], SCOPA-MOTOR scores [$F(1,36) = .80$, $p = .38$], MoCA [$F(1,36) = 0.13$, $p = .72$] and LEDD [$F(1,36)=2.44$, $p=.13$] and clinical severity CISI-PD[55] [$F(1,35) = 3.47$, $p = .07$], however PD-VH patients were on average characterised by greater PD severity (1–7 points=mild; 8–14: moderate; ≥15 severe). We report in Table 1 the CISI-PD total score and it scores of each of the subscales, with PD-

VH saying they feel more cognitively impaired than PD-noVH, but similar to PD-noVH for motor symptoms and motor complications. See **Table 1.**

- Table 1 -

In addition to the lower CISI-PD cognitive score, PD-VH differed on non-motor symptoms (NMSS, see **SI1**)[56]. When breaking down this score into domains we find that PD-noVH disclose more gastrointestinal symptoms [F(1,35)=21.74, p <.001] and PD-VH disclose more attentional [F(1,35)=10.64, p =.002], perceptual (including VH and double vision) [F(1,35)=55.72, p <.001] and sleep [F(1,35)=13.43, p =.001] symptoms (see **Figure 2** and **Supplementary Information 5b**).

- Figure 2 -

PD-VH patients were also administered PD-psychosis specific questionnaires (see SI1 for details) with general hallucinations score at the SAPS-PD of 10.45 ± 5.5. Out of 20 patients, only 1 presented delusions, and they retained insight. All patients presented hallucinations in the visual modality, some patients presented also other types of hallucinations (**SI1**), with further details from the NEVHI described in **Table SI1b.**

Within group analysis of ERP components.

We conducted an analysis to compare the standard and the deviant components in each group.

For PD-noVH at the parieto-occipital channels O1, OZ, POZ, PO3, PO4, PO5, PO6, PO8 a significant difference was found between rare deviant and standard at ~100-125ms, with the rare being more negative than the standard. At PO7 instead, whereas the 100-120ms waveform has a negative peak, we found a significant difference at the P300 level, which was also observed at PO8 and PO6, with the rare being more positive consistently with the P300 waveform. When correcting for multiple comparisons the difference in PD-noVH remains significant for POZ in the 100-150 ms interval and at PO4 at 100ms. PD-noVH patients also had greater negativity for the deviant at PZ at 100-170ms and greater positivity for the deviant at ~300-400ms. The negativity at 100-170ms was significant also after FDR correction. A similar result was observed for P2 where the P300 was significant after FDR correction. At P3, P4 and P5 we also observed significantly greater negativity at 100-150ms (FDR corrected) and greater positivity in the 300-400ms interval, surviving FDR correction in P4 and P6 (see **Figure3**.)

For Review Only

Frontal channels. For PD-noVH a significant difference was identified at F1, F3, F5, F7 peaking at ~160ms, whereby the deviant waveform was more *positive* in the PD-noVH, however this did not survive FDR correction. At F2 we observed the same pattern over a longer latency (100-250ms) and when FDR corrected the comparison remains significant in the 150ms interval and a similar pattern is observed for F4. At F6 and F8 the positive difference survives FDR correction in the 170-180ms interval. At FZ (150-220ms) and FPZ (~180ms), a greater positivity and positive difference is observed. However, this does not survive FDR correction.

PD-VH. Parietoccipital channels: At POZ we found a difference at the level of the P300, which did not survive FDR correction. We observed greater positivity at > 300ms for P4 (not FDR corrected). We observed a greater negativity at P2, however with a noisy waveform and a late (400ms) greater positivity at P1 (not surviving FDR correction).

Frontal channels: A significant difference in the amplitudes with the deviant being more positive was found for FZ, F1, F2 and F3 at ~180-190ms (not surviving FDR correction). No significant difference between the deviant and standard waveform were present in the *PD-VH* group in the other frontal electrodes.

- **Figure 3** -

To better explore the difference between the standard and the deviant in each group we analysed significant differences at the whole-brain level using scalp topography (correcting for multiple comparisons, *p*FDR<.05). For PD-noVH, in the 100-180ms interval, selected to include both the earlier MMN observed at the parieto-occipital channels and the slightly delayed mismatch positivity observed frontally, we observed the pattern very clearly with the scalpogram. The whole-brain analysis of channels where the two components significantly differed at this interval revealing a right frontal (F2, F4, AF4) and a more left parieto-occipital (POZ, PZ, P1-P5) pattern, consistent with the more focused analysis reported above. For PD-VH the scalpogram shows a slightly more negative pattern frontally for the deviant if compared to the standard, with no significant difference across channels.

- **Figure 4** -

https://mc.manuscriptcentral.com/braincom

We computed the vMMN by subtracting the standard amplitude to the rare deviant amplitude in the 100-125ms interval identified with the waveform inspection and compared PD-VH and PD-noVH with a one-way ANOVA on the channels where we observed a waveform resembling the MMN. We compared the two groups on the channels where we found a significant difference between standard and deviant that survived *FDR* correction in the within group analysis, in at least one of the two groups: F2, F4, F6 and F8; POZ and PO4. We explored parietal differences at both earlier and later latencies.

Parieto-occipital channels: Amplitude between groups significantly differed at POZ [F (1,36) = 4.32, MS = 289.821, *p* = .045], with PD-noVH having a mean amplitude of -4.09 (sd = 6.73) and PD-VH of. 1.51 (sd = 9.48). P2 we find a greater negativity for PD-noVH (100-180ms interval) (mean =-6.37, sd =1.11) > PD-VH (mean =-1.05, sd =1.21), [F(1,36)= 4.19, p = .048] (see **Supplementary Information 7** for additional exploratory analysis). No other electrodes at this level showed a significant difference (FDR corrected) however, a difference in the visual evoked potential waveform can be appreciated; indeed PD-noVH have a prominent posterior negative wave at 100ms which is missing or attenuated in PD-VH (see **Figure 3-5**).

Frontal channels: Upon visual inspection and consistently with the EEGlab analysis, at a slightly later latency (100-180ms) than observed for MMN for the occipital and parieto-occipital channels, at the frontal channels we have observed a mismatch *positivity*: F2 [F (1,36) = 4.12, *p* = .05] with PD-noVH having vMMN amplitude =9.80 (sd =11.24) and PD-VH mean =-0.04 (sd = 17.58), F4 [F (1,36) = 5.85, *p* = .021] (PD-noVH vMMN =9.52 (sd =10.94) and PD-VH mean =-3.1 (sd = 19.54)), F6 [F (1,36) = 4.03, *p* = .05] (PD-noVH =7.06 (sd =12.32) and PD-VH =-1.14 (sd = 17.91), and AF4, [F(1,36)=4.68, p =.037] (PD-noVH = 10.92, sd = 14.08, PD-VH = -2.38, sd = 22.38).

Parietal channels: P2 was the only parietal channel surviving FDR correction, and when comparing the MMN at the 100-180ms latency we find a greater negativity for PD-noVH (mean =-6.37, sd =1.11) > PD-VH (mean =-1.05, sd =1.21), [F(1,36)= 4.19, p = .048] (see **Supplementary Information 7** for additional exploratory analysis).

We ran a further FDR correction on these comparisons (n=10), with all p_s =.05.

We also ran an EEGlab between groups 2x2 design analysis showing consistent results with those presented in the main text (**Supplementary Information 8**).

- Figure 5 -

Correlation with measures of hallucinations. Neither SAPS-PD nor NEVHI correlated with disease duration, LEDD, non-motor symptoms, MoCA and SCOPA-motor scores (for details **SI9, Figure 6**).

For correlations between rare-standard mismatch and hallucinations scores, we found a significant negative correlation between SAPS-PD score and mismatch at FZ (r = -.54, p =.007) with greater hallucinations scores corresponding to a smaller amplitude difference between standard and rare. We saw a positive correlation between NEVHI temporal severity for complex VH and POZ (r = .49, p =.014), with participants with a more negative MMN having lower complex VH scores. The result is the same when conducting the analysis removing the participant who said they had not had complex VH/had not had any in the past month (r = .53, p =.017) (**Figure 6**).

- Figure 6 -

Study 2. *Saracatinib and psylocibin behavioural study in healthy volunteers: subjective effects*

The main outcome for this study was the first question on the questionnaire taken from Carhart-Harris et al. (2011) asking “How intense were the drug effects when at their peak”. Two participants did not answer this question in one session and were excluded. For the remaining participants (n=18) there was a statistically significant reduction in reported intensity during the psilocybin + saracatinib session compared to psilocybin session (t (17) = 1.99, one-sided p = 0.031, d=0.63; mean psilocybin = 85.4, SE = 3.5, mean psilocybin + sarcatinib = 76.4, SE = 3.6). We explored the other questions, none of which changed.

Study 3*. PD-VH only saracatinib pilot study*

All participants were males. As 5 participants completed the drug study and each participant was used as their own control in the analysis, we report the baseline demographics and clinical data in **Supplementary Information 10**.

Hallucinations scores. A Wilcoxon signed rank test of SAPS-PD scores in the placebo and drug arms of the study showed a significant reduction of VH scores SAPS-PD [SAPS-PD$_{PLA}$= 9.8 ± 3.11, SAPS-PD$_{DRUG}$= 5.8 ±2.17, Z = -2.032, p = .04]. NEVHI temporal severity scores for complex VH [NEVHI$_{PLA}$=

For Review Only

3.8 ± 1.79, $NEVHI_{DRUG}$= 3.4 ± 3.4, Z = -.18, p =.84] were not significantly different. A similar result was found for minor VH (**SI10c**).

vMMN amplitude was shifted when participants had been on the study drug for two weeks. This is significant at frontal electrodes, whereby vMMN (rare-standard) amplitude is significantly more negative at ~200-250ms latencies (**SI 10** for individual waveforms and further details). The Wilcoxon test on the best frontal electrode for each participant showed that there was a significant difference [Z = -2.023, p= 0.043] with a greater negative vMMN for the drug vs. the placebo period [$vMMN_{PLA}$= -0.12 ± 1.09, $vMMN_{DRUG}$= -1.66 ±0.69]. At the fixed effects analysis, we found that the vMMN was significantly greater at the drug vs. the placebo arm of the study [Z= -3.11, p = .002, $vMMN_{PLA}$= -0.14 ± 0.65, $vMMN_{DRUG}$= -1.12 ±0.83], and the rare being more negative during the drug arm [$rare_{PLA}$= -0.83 ± 1.35, $rare_{DRUG}$= -2.23 ± 1.23].

No significant differences were found at parieto-occipital channels (**SI10**).

- **Figure 7** -

Discussion

We have presented clear differences in vMMN between patients with Parkinson's and Parkinson's psychosis, focusing on electrodes typically associated with the aMMN and vMMN, and experimentally validated and piloted a potential mechanism to reduce impairments. When analysing differences between the deviant and the standard ERP components we found that PD-noVH have a vMMN (**Figure 3-5**), consistent with the aMMN in PD[18,57,58]. When comparing the groups, PD-VH had reduced vMMN amplitude at the parieto-occipital and frontal channels (**Figure 3,5**). Any laterality effects are unlikely to be due to laterality of symptoms as patients did not differ in this. Reduced MMN amplitude is one of the most consistent and replicated results in psychosis[9,10,12,13] and thus it is consistent with our initial hypothesis that these patients would respond differently to the sensory environment presented with our vMMN task. A recent GWAS study found MMN to be a superior psychosis marker if compared to others previously associated with psychosis risk, such as lateral ventricular volume and verbal learning[59]. Our results confirm the sensitivity of this measure when comparing patients with and without hallucinations and related perceptual phenomena within the same diagnostic group.

As there is no literature about MMN in PD-VH, we can speculate that the reduced MMN reflects the greater discrepancy between the sensory input and top-down predictions in PD-VH, whereby the brain is impaired in its ability to resolve such a discrepancy, as proposed by the predictive coding account of psychosis symptoms[60]. A more in-depth analysis of models of VH in PD has been recently carried out

For Review Only

to create a consensus framework for VH in Parkinson's[61]. Within this framework, the authors reconcile different models of VH, considering 'ascending' sensory disturbances (i.e. perceptual deficits and environmental factors), 'descending' factors (i.e. overly influent and biasing expectancies, semantic knowledge and memory traces, attentional deficits at spatial, object and orientation level). In this view, defective visual input and biasing (prefrontal) signals concur to the generation of VH, with poor frontal attentional (redirection) and control mechanisms and a potentially impaired frontotemporal semantic processing also playing a role (for an extensive discussion[61]). Our results are also congruent with previous neuroimaging studies of PD-VH in different modalities[17,21,62,63] .

Concerning the finding of differences in frontal and visual regions in our analyses, we hypothesize that the frontal and occipital electrodes are picking up local sources, rather than a dipole halfway between them. The later frontal mismatch we find (100-180ms) if compared to parieto-occipital channels (100-125ms) could be explained in terms of hierarchy in the cortex. We know that 'higher' brain regions respond to incoming information changing over longer intervals[64]. Ascending connections from lower areas, such as the primary visual cortex, project to frontal areas arising from cortical layer III and target layer IV. It has thus been proposed that connections between brain regions at different hierarchical levels may represent the neural correlates of the probabilities mapping hidden causes (higher regions) to the sensory input (lower regions)[60]. In this case, ascending connections between primary visual and frontal regions would carry prediction error signals. In a neurodegenerative condition such as PD we also need to consider that it can be characterized by widespread grey matter loss, especially in progressive subtypes, and such a reduction would correspond to a reduction in the models that the activity patterns across the neurons of a region can represent, thus limiting their accuracy[65]. In addition, dopamine has been proposed to be linked to precision of prior beliefs, and it has been suggested to play a role in movement initiation deficits in PD[66] and in the optimisation of sequences of actions[67]. It is thus possible that dopamine depletion plays a role as well, but we know from previous research[17] that dopaminergic deficits alone cannot account for visual hallucinations and indeed a role of cholinergic[61,68] and serotonergic[24] dysfunction has been proposed.

In the current study, we have tested the potential role of the serotonergic 5-HT_{2A} cascade in VH in PD with hallucinations. The receptor trafficking hypothesis was first experimentally tested in healthy volunteers by pre-dosing a psilocybin administration with saracatinib, providing support for the role of the Gi/o-dependent pathway in the psychedelic effect. The study was not designed to test for a selective effect on different visual experiences (e.g. pareidolia vs. complex VH) but we found a general reduction in the intensity of experience. A longer duration of dosing in a small sample of patients also supported the hypothesis of the involvement of the Gi/o-dependent pathway in PD-VH. Changes in the 5-HT_{2A} signaling pathway following SRC-kinase inhibition appear to modulate the vMMN at frontal channels (**SI 10**), but not at occipital sites. 5-HT_{2A} receptors are widespread in the cerebral cortex, particularly in frontal and occipital areas[69] and 5-HT_{2A} receptors' density is affected in PD[25,70] and in schizophrenia[71]. In addition, serotonin and glutamate ligands have been found to tune the pattern of G-protein coupling

For Review Only

in the 5-HT_{2A}/$mGlu_2R$ complex linked to the pathophysiology of schizophrenia[30], suggesting 5-HT_{2A} and $mGlu_2R$ as potential targets for antipsychotic drug development, which our previous mechanistic work has supported[72], although trials are yet to be successful in patients[73]. Our results in PD-VH, together with previous findings linking 5-HT_{2A} receptors density to differences in cortical thickness between PD-noVH and PD-VH patients[21] adds further to the potential role of serotonergic modulation of visual hallucinations and related symptoms in Parkinson's disease. In our small sample, we observed a reduction in global rating of hallucinations as measured by the SAPS-PD and altered vMMN. We can only speculate why the effect of SRC-kinase inhibition via saracatinib appears to modulate the vMMN only at frontal rather than occipital sites. A possibility is that damage between frontal and occipital areas in the serotonergic pathway prevents the drug from acting evenly throughout the 5-HT_{2A} pathway. Another possibility is that it might take longer than 14 days for the drug to be fully effective.

Another interesting result is the different sign of the MMN we observed at different cortical sites. We found visual MMN at parieto-occipital sites and a 'positivity' at frontal channels (**Figure 3**). Other studies have reported visual mismatch positivity at central[12,14] and frontal[74] sites, where we also found longer latencies at frontal electrodes, consistent with previous research[75]. Among the possible explanations for this is that, as a dual generator is proposed for auditory MMN (for an in-depth review[36]), with a temporal and a frontal generator, this could also be the case for visual MMN, with a parieto-occipital and a frontal generator or components. However, it is unclear why the polarity of the MMN activity is different in each region and, to confirm this suggestion, detailed source localisation analysis would be important. We can speculate that these parieto-occipital and frontal components may be part of a network of hierarchical cortical sources[2] with these regions being in turn responsible for the comparison of the standard and deviant and the subsequent direction of attention, as previously found at the temporal and frontal level for aMMN[76]. Indeed, the P300 component is thought to signal the full attention switch[77] but is not always there; in our study, we found it at some, but not all, parietal and parieto-occipital in PD-noVH only (**Figure 2**) (a negative N300 component was also observed at frontal electrodes but was rarely significant).

We also explored the relationship between different aspects of VH intensity and vMMN amplitude, finding a correlation between SAPS-PD and NEVHI (temporal severity of complex VH) scores and (deviant – standard) amplitude at FZ and POZ, whereby the more severe the symptoms, the smaller the difference between the two waveforms.

Limitations

The study has some limitations. First, we could not enroll the original target number for each group for Study3, thus limiting the power of our analyses. The study started shortly before the COVID-19 pandemic and was severely impacted by both lockdowns. Since the safety of patients who were particularly at risk was the primary concern, recruitment slowed significantly. This impacted more

For Review Only

heavily on the drug study, for which only 5 participants completed both arms before the drug company stopped manufacturing the study drug, which is a second limitation. Second, unfortunately, due to the challenges in recruitment over the pandemic, we did not include sufficient participants to have different blocks of patients administered different versions of the task featuring a different orientation for standard and deviant stimuli.

Conclusions

MMN is sensitive to PD with hallucinations, and we also proposed and tested a mechanism through which PD MMN impairments may be reduced in PD psychosis, validated through a psychedelic model of altered visual experience.

We demonstrate that MMN is a sensitive and potentially useful tool in experimental testing of mechanisms to reduce symptoms in PD psychosis with an exemplar pilot case series with positive results.

Data availability

Summary data (EEG pre-processed amplitude for standard and deviant components (time range for each epoch from -100 to 500ms) is made available; clinical anonymised data (SCOPA-MOTOR, LEDD, disease duration, age, sex, SAPS-PD, NEVHI-complex, NPI, NMSS) is also made available; peak dataset for correlations between POZ and FZ mismatch and NEVHI and SAPS-PD in the VH group are made available). Raw EEG .CNT files are not at the moment available as these are currently being processed for further analyses. The data can be made available upon reasonable request (e.g. for collaborations).

Acknowledgments and Funding

This is independent research funded by the Medical Research Council (grant number MR/R005931/1 awarded to M.A.M., D.F, K.R.C and Dag Aarsland, and MRC grant MR/K015192 awarded to M.A.M and D.N.) and carried out at the National Institute for Health and Care Research (NIHR) Maudsley Biomedical Research Centre (BRC). The views expressed are those of the author(s) and not necessarily those of the MRC, the NIHR or the Department of Health and Social Care.

MV was supported by MRC and is currently supported by ARUK (grant ARUK-RF2022B-002).

CHWG is supported by the Medical Research Council (MR/W029235/1) and the NIHR Cambridge Biomedical Research Centre (NIHR203312). The views expressed are those of the authors and not necessarily those of the NIHR or the Department of Health and Social Care.

We thank the CRISP (patient representation) group for support and advice during the inception of this study and all our participants for taking part in the research and their partners for their support on the study days.

We thank AstraZeneca UK for providing the study drug.

We also thank Robin Carhart-Harris for advice on the psilocybin study and Dag Aarsland for his support with the development of important study ideas.

We thank Stephanie Stephenson for training the study researchers with clinical trials procedures and for her support throughout the duration of the study; and we thank Caroline Woolridge for her support with study logistics and ethics applications.

For Review Only

We thank Dr Daniel van Wamelen, Dr Claudia Lazcano Ocampo for their help with the study. We thank Simon Hill for the contribution to the EEG task and recording setup.
We thank Paulo Silva for the contribution to the EEG task programming. We also wish to thank Dr Maria Teresa Pascarelli for helpful advice on EEG analysis.
We thank Glynis Ivin and David Taylor South London and Maudsley pharmacy for their support.
We thank the KCL Clinical Research Facility staff for their support with the study and the CRF director Elka Giemza.

For the purpose of open access, the author has applied a Creative Commons Attribution (CC BY) licence to any Author Accepted Manuscript version arising from this submission.

Author contributions

MAM conceptualised Study 1-2-3. MAM and DF conceptualised Study 3.
MAM, DF and MV developed the EEG procedure for Study 1 and Study 3.
MV conducted study visits and collected and analysed the EEG, behavioural and clinical data and wrote the manuscript with MAM.
DF provided important supervision during the processing of EEG data and the analyses of the EEG pilot study and the online pilot task.
NM was the main study physician for all studies.
GP helped collect the PD-noVH data and analysed the behavioural data for the online task and helped with the the behavioural task analysis for this group.
MM and SD helped with study set up and data collection for Study 1 and 3.
DN was involved in designing the study and obtaining the funding of Study 2. ASG recruited and tested all participants and analyses data for Study2.
KRC, YT, LB, CWG, VL pre-screened and referred patients.

MV and MAM wrote the manuscript. All authors contributed to manuscript revision and approval.

For Review Only

Figures and Tables

Figure 1. *vMMN task sequence of events and channels of interest in our analyses.*

Figure 2. *NMSS by symptom cluster. ** p <.005, *** p =<.001 Median (blue dot) and interquartile range (black bar in the center of each plot) are represented in the figure.*

Figure 3. *Standard (violet) and deviant (pink) waveforms in PD-noVH and PD-VH in the within group analysis. The black bars indicate the latencies at which the two waveforms significantly differ (p <.05). The blue lines indicate the latencies at which such differences survive multiple comparisons correction (pFDR < .05).*

Figure 4. *Within group scalp topography in the 100-180ms (EEGlab). Top row: scalp topography for PD-noVH. The channels represented with a red dot are those where a significant difference between standard and deviant (deviant was more negative posteriorly or more positive frontally) was found. Bottom row: the same analysis was used for PD-VH patients. The lateral insets show (clearly in PD-noVH) the parieto-occipital negative and frontal positive pattern we observed in the vMMN analysis (plots created with ggplot2 in R).*

Figure 5*. Electrodes (red dots) that presented significant deviant-standard vMMN amplitude differences in the groups (p <.05, corrected for multiple comparisons with the Benjamini-Hochberg correction). Electrodes pictured in red on the map: POZ, P2, F2, F4, F6, AF4. F4, F6, F7, F5, P1, P2, P3, P4, PZ, POZ. Standard (violet) and deviant (pink) waveforms for PD-VH and PD-noVH. The shaded area represents the variance.*

Figure 6*. a) Proportion of patients with a specific type of visual hallucinations as detected by the NEVHI interview. The figures used to represent the symptom categories are in part derived from ffytche et al., 2017; b) Pearson's product moment correlation coefficients for the clinical variables and*

For Review Only

neuropsychiatric assessments (PD-VH only) c) Left: SAPS-PD scores as a function of mismatch positivity and negativity at FZ after considering the sign of the mismatch between standard and deviant. Right: NEVHI temporal severity for complex VH–as a function of mismatch positivity POZ._ The shaded green area represents those values that correspond to a normal deviant-standard difference, negative in the case of POZ and in this study positive for FZ. The red shaded area contains the deviant-standard values whereby the standard was greater than the deviant or there was no difference.

Figure 7. *Left column: frontal electrodes in the drug arm, Right column: frontal electrodes in the placebo arm. Deviant is shown in pink, standard in violet. The difference between the drug and placebo was captured in a comparison of individual peaks, described in the text; individual waveforms are displayed in SI10.*

For Review Only

Table1. *Participants demographics and clinical information. We report disease duration (in years), LEDD (levodopa equivalent dose), Montreal Cognitive assessment (MoCA) scores (participants were not included if they presented a score lower to 22), SCOPA-motor* total score, NMSS (non-motor symptom scale) total score, Clinical Impression of Severity Index for PD, completed by a Parkinson's neurologist upon physical exam and the breakdown of the CISI-PD in the four categories (motor severity, motor complications, cognitive status and disability; cognitive status and disability are computed based on the self-reported deficits of the patients). *One PD-noVH participant did not have a SCOPA-motor or UPDRS-III score and was excluded from this comparison*

References

1. Näätänen,R.,Kujala,T.,Kreegipuu,K.,Carlson,S.,Escera,C.,Baldeweg,T.,etal. (2011). The mismatch negativity: an index of cognitive decline in neuropsychiatric and neurological diseases and in ageing. *Brain* 134, 3435–3453.doi: 10.1093/brain/awr064

2. Garrido, M. I., Kilner, J. M., Stephan, K. E., & Friston, K. J. (2009). The mismatch negativity: a review of underlying mechanisms. *Clinical neurophysiology*, *120*(3), 453-463.

3. Feldman, H., & Friston, K. J. (2010). Attention, uncertainty, and free-energy. *Frontiers in human neuroscience*, *4*, 215.
4. Friston K. A theory of cortical responses. Philos Trans R Soc Lond B Biol Sci (2005) 360(1456):815–36. doi: 10.1098/rstb.2005.1622

5. Rao, R. P., & Ballard, D. H. (1999). Predictive coding in the visual cortex:a functional interpretation of some extra-classical receptive-field effects. *Nature neuroscience*, *2*(1), 79-87.

6. Sterzer, P., Adams, R. A., Fletcher, P., Frith, C., Lawrie, S. M., Muckli, L., ... & Corlett, P. R. (2018). The predictive coding account of psychosis. *Biological psychiatry*, *84*(9), 634-643.

7. Adams, R. A., Stephan, K. E., Brown, H. R., Frith, C. D., & Friston, K. J. (2013). The computational anatomy of psychosis. *Frontiers in psychiatry*, *4*, 47.

8. Fong, C. Y., Law, W. H. C., Uka, T., & Koike, S. (2020). Auditory mismatch negativity under predictive coding framework and its role in psychotic disorders. *Frontiers in Psychiatry*, *11*, 557932.

9. Todd, J., Michie, P. T., Schall, U., Ward, P. B., & Catts, S. V. (2012). Mismatch negativity (MMN) reduction in schizophrenia—Impaired prediction-error generation, estimation or salience?. *International Journal of Psychophysiology*, *83*(2), 222-231.

10. Umbricht, D., & Krljes, S. (2005). Mismatch negativity in schizophrenia: a meta-analysis. *Schizophrenia research*, *76*(1), 1-23.

11. He, X., Zhang, J., Zhang, Z., Go, R., Wu, J., Li, C., ... & Chen, D. (2020). Effects of visual attentional load on the tactile sensory memory indexed by somatosensory mismatch negativity. *Frontiers in Neuroinformatics*, *14*, 575078.

12. Csukly, G., Stefanics, G., Komlósi, S., Czigler, I., & Czobor, P. (2013). Emotion-related visual mismatch responses in schizophrenia: impairments and correlations with emotion recognition. *PLoS One*, *8*(10), e75444.

13. Urban, A., Kremláček, J., Masopust, J., & Libiger, J. (2008). Visual mismatch negativity among patients with schizophrenia. *Schizophrenia research*, *102*(1-3), 320-328.

14. Stefanics, G., Kremláček, J., & Czigler, I. (2014). Visual mismatch negativity: a predictive coding view. *Frontiers in human neuroscience*, *8*, 666.

15. Rodriguez-Blazquez, C., Schrag, A., Rizos, A., Chaudhuri, K. R., Martinez-Martin, P., & Weintraub, D. (2021). Prevalence of non-motor symptoms and non-motor fluctuations in Parkinson's disease using the MDS-NMS. *Movement disorders clinical practice*, *8*(2), 231-239.

16. Chang, A., & Fox, S. H. (2016). Psychosis in Parkinson's disease: epidemiology, pathophysiology, and management. *Drugs*, *76*(11), 1093-1118.

17. Ffytche, D. H., Creese, B., Politis, M., Chaudhuri, K. R., Weintraub, D., Ballard, C., & Aarsland, D. (2017). The psychosis spectrum in Parkinson disease. *Nature Reviews Neurology*, *13*(2), 81-95.

18. Solís-Vivanco, R., Ricardo-Garcell, J., Rodríguez-Camacho, M., Prado-Alcalá, R. A., Rodríguez, U., Rodríguez-Violante, M., & Rodríguez-Agudelo, Y. (2011). Involuntary attention impairment in early Parkinson's disease: an event-related potential study. *Neuroscience letters*, *495*(2), 144-149.

19. Pekkonen, E., Jousmäki, V., Reinikainen, K., & Partanen, J. (1995). Automatic auditory discrimination is impaired in Parkinson's disease. *Electroencephalography and clinical neurophysiology*, *95*(1), 47-52.

20. Kremláček, J., Kreegipuu, K., Tales, A., Astikainen, P., Poldver, N., Näätänen, R., & Stefanics, G. (2016). Visual mismatch negativity (vMMN): A review and meta-analysis of studies in psychiatric and neurological disorders. *Cortex*, *80*, 76-112.

21. Vignando, M., Ffytche, D., Lewis, S. J., Lee, P. H., Chung, S. J., Weil, R. S., ... & Mehta, M. A. (2022). Mapping brain structural differences and neuroreceptor correlates in Parkinson's disease visual hallucinations. *Nature communications*, *13*(1), 519.

22. Shine, J. M., Muller, A. J., O'Callaghan, C., Hornberger, M., Halliday, G. M., & Lewis, S. J. (2015). Abnormal connectivity between the default mode and the visual system underlies the manifestation of

For Review Only

visual hallucinations in Parkinson's disease: a task-based fMRI study. *npj Parkinson's Disease*, *1*(1), 1-8.

23. Thomas, G. E., Zeidman, P., Sultana, T., Zarkali, A., Razi, A., & Weil, R. S. (2023). Changes in both top-down and bottom-up effective connectivity drive visual hallucinations in Parkinson's disease. *Brain Communications*, *5*(1), fcac329

24. Ballanger, B., Strafella, A. P., van Eimeren, T., Zurowski, M., Rusjan, P. M., Houle, S., & Fox, S. H. (2010). Serotonin 2A receptors and visual hallucinations in Parkinson disease. *Archives of neurology*, *67*(4), 416-421.

25. Huot, P., Johnston, T. H., Darr, T., Hazrati, L. N., Visanji, N. P., Pires, D., ... & Fox, S. H. (2010). Increased 5-HT2A receptors in the temporal cortex of parkinsonian patients with visual hallucinations. *Movement disorders*, *25*(10), 1399-1408.

26. Cho, S. S., Strafella, A. P., Duff-Canning, S., Zurowski, M., Vijverman, A. C., Bruno, V., ... & Fox, S. H. (2017). The relationship between serotonin-2A receptor and cognitive functions in nondemented Parkinson's disease patients with visual hallucinations. *Movement disorders clinical practice*, *4*(5), 698-709.

27. Zhang, G., & Stackman Jr, R. W. (2015). The role of serotonin 5-HT2A receptors in memory and cognition. *Frontiers in pharmacology*, *6*, 225.

28. Kometer, M., Schmidt, A., Jäncke, L., & Vollenweider, F. X. (2013). Activation of serotonin 2A receptors underlies the psilocybin-induced effects on α oscillations, N170 visual-evoked potentials, and visual hallucinations. *Journal of Neuroscience*, *33*(25), 10544-10551.

29. González-Maeso, J., Weisstaub, N. V., Zhou, M., Chan, P., Ivic, L., Ang, R., ... & Gingrich, J. A. (2007). Hallucinogens recruit specific cortical 5-HT2A receptor-mediated signaling pathways to affect behavior. *Neuron*, *53*(3), 439-452
30. Muguruza, C., Meana, J. J., & Callado, L. F. (2016). Group II metabotropic glutamate receptors as targets for novel antipsychotic drugs. *Frontiers in pharmacology*, *7*, 130.

31. Gonzalez-Maeso, J., & Sealfon, S. C. (2009). Psychedelics and schizophrenia. *Trends in neurosciences*, *32*(4), 225-232.

32. Byock, I. (2018). Taking psychedelics seriously. *Journal of palliative medicine*, *21*(4), 417-421.

33. Deouell, L. Y. (2007). The frontal generator of the mismatch negativity revisited. *Journal of Psychophysiology*, *21*(3-4), 188-203.

34. Rinne, T., Alho, K., Ilmoniemi, R. J., Virtanen, J., & Näätänen, R. (2000). Separate time behaviors of the temporal and frontal mismatch negativity sources. *Neuroimage*, *12*(1), 14-19.

35. Näätänen, R., & Winkler, I. (1999). The concept of auditory stimulus representation in cognitive neuroscience. *Psychological bulletin*, *125*(6), 826.

36. Fitzgerald, K., & Todd, J. (2020). Making sense of mismatch negativity. *Frontiers in Psychiatry*, *11*, 468.

37. Renouf, S., Ffytche, D., Pinto, R., Murray, J., & Lawrence, V. (2018). Visual hallucinations in dementia and Parkinson's disease: a qualitative exploration of patient and caregiver experiences. *International Journal of Geriatric Psychiatry*, *33*(10), 1327-1334.

38. Cummings JL, Mega M, Gray K, Rosenberg-Thompson S, Carusi DA, Gornbein J. The Neuropsychiatric Inventory: comprehensive assessment of psychopathology in dementia. *Neurology.* 1994;44(12):2308–2314. doi: 10.1212/WNL.44.12.2308

For Review Only

39. Voss, T., Bahr, D., Cummings, J., Mills, R., Ravina, B. & Williams, H. 2013. Performance of a shortened Scale for Assessment of Positive Symptoms for Parkinson's disease psychosis. *Parkinsonism Relat Disord,* 19**,** 295-9.

40. D'Antonio, F., Boccia, M., Di Vita, A., Suppa, A., Fabbrini, A., Canevelli, M., ... & Ffytche, D. (2022). Visual hallucinations in Lewy body disease: pathophysiological insights from phenomenology. *Journal of Neurology*, *269*(7), 3636-3652.

41. Qian, X., Liu, Y. I., Xiao, B., Gao, L. I., Li, S., Dang, L., ... & Zhao, L. (2014). The visual mismatch negativity (vMMN): toward the optimal paradigm. *International journal of psychophysiology*, *93*(3), 311-315.

42. Melikyan, Z. A., Malek-Ahmadi, M., O'Connor, K., Atri, A., Kawas, C. H., & Corrada, M. M. (2021). Norms and equivalences for MoCA-30, MoCA-22, and MMSE in the oldest-old. *Aging clinical and experimental research*, *33*(12), 3303-3311.

43. Verbaan, D., Van Rooden, S. M., Benit, C. P., van Zwet, E. W., Marinus, J., & van Hilten, J. J. (2011). SPES/SCOPA and MDS-UPDRS: formulas for converting scores of two motor scales in Parkinson's disease. *Parkinsonism & related disorders*, *17*(8), 632-634.

44. Brunner, C., Delorme, A., & Makeig, S. (2013). Eeglab–an open source matlab toolbox for electrophysiological research. *Biomedical Engineering/Biomedizinische Technik*, *58*(SI-1-Track-G), 000010151520134182.

45. Lopez-Calderon, J., & Luck, S. J. (2014). ERPLAB: an open-source toolbox for the analysis of event-related potentials. *Frontiers in human neuroscience*, *8*, 213.

46. R Core Team (2021). R: A language and environment for statistical computing. R Foundation for Statistical Computing, Vienna, Austria. https://www.R-project.org/.

47. Hadley Wickham, Romain François, Lionel Henry and Kirill Müller (2021). dplyr: A Grammar of Data Manipulation. R package version 1.0.4. https://CRAN.R-project.org/package=dplyr

48. Alboukadel Kassambara (2023). ggcorrplot: Visualization of a Correlation Matrix using 'ggplot2'. R package version 0.1.4.1. https://CRAN.R-project.org/package=ggcorrplot

49. Stephane Champely (2018). PairedData: Paired Data Analysis. R package version 1.1.1. https://CRAN.R-project.org/package=PairedData

50. Marc J. Mazerolle (2020) AICcmodavg: Model selection and multimodel inference based on (Q)AIC(c). R package version 2.3-1. https://cran.r project.org/package=AICcmodavg.

51. Wickham et al., (2019). Welcome to the tidyverse. Journal of Open Source Software, 4(43), 1686, https://doi.org/10.21105/joss.01686

52. Alboukadel Kassambara (2020). ggpubr: 'ggplot2' Based Publication Ready Plots. R package version 0.4.0. https://CRAN.R-project.org/package=ggpubr

53. David Robinson, Alex Hayes and Simon Couch (2021). broom: Convert Statistical Objects into Tidy Tibbles. R package version 0.7.4. https://CRAN.R-project.org/package=broom

54. Delorme, A., Mullen, T., Kothe, C., Acar, Z. A., Bigdely-Shamlo, N., Vankov, A., & Makeig, S. (2011). EEGLAB, SIFT, NFT, BCILAB, and ERICA: new tools for advanced EEG processing. *Computational intelligence and neuroscience*, *2011*, 10-10.

55. Martinez-Martin P, Forjaz MJ, Cubo E, Frades B, de Pedro Cuesta J, et al. (2006) Global versus factor-related impression of severity in Parkinson's disease: A new clinimetric index (CISI-PD). *Mov Disord* 21: 208–214.M

For Review Only

56. Chaudhuri, K. R., Martinez-Martin, P., Brown, R. G., Sethi, K., Stocchi, F., Odin, P., ... & Schapira, A. H. (2007). The metric properties of a novel non-motor symptoms scale for Parkinson's disease: results from an international pilot study. *Movement disorders*, *22*(13), 1901-1911.

57. Pekkonen, E., Ahveninen, J., & Teräväinen, H. (2000). Auditory discrimination is preserved in Parkinson's disease. *Neuroimage*, *5*(11), S142.

58. Brønnick, K. S., Nordby, H., Larsen, J. P., & Aarsland, D. (2010). Disturbance of automatic auditory change detection in dementia associated with Parkinson's disease: a mismatch negativity study. *Neurobiology of aging*, *31*(1), 104-113.

59. Bhat, A., Irizar, H., Thygesen, J. H., Kuchenbaecker, K., Pain, O., Adams, R. A., ... & Bramon, E. (2021). Transcriptome-wide association study reveals two genes that influence mismatch negativity. *Cell reports*, *34*(11), 108868

60. Friston, K. J., Parr, T., & de Vries, B. (2017). The graphical brain: belief propagation and active inference. *Network neuroscience*, *1*(4), 381-414.

61. Collerton, D., Barnes, J., Diederich, N. J., Dudley, R., Friston, K., Goetz, C. G., ... & Weil, R. S. (2023). Understanding visual hallucinations: a new synthesis. *Neuroscience & Biobehavioral Reviews*, *150*, 105208.

62. Firbank, M. J., Parikh, J., Murphy, N., Killen, A., Allan, C. L., Collerton, D., ... & Taylor, J. P. (2018). Reduced occipital GABA in Parkinson disease with visual hallucinations. *Neurology*, *91*(7), e675-e685.

63. Lenka, A., Jhunjhunwala, K. R., Saini, J., & Pal, P. K. (2015). Structural and functional neuroimaging in patients with Parkinson's disease and visual hallucinations: a critical review. *Parkinsonism & related disorders*, *21*(7), 683-691.

64. Murray, J. D., Bernacchia, A., Freedman, D. J., Romo, R., Wallis, J. D., Cai, X., ... & Wang, X. J. (2014). A hierarchy of intrinsic timescales across primate cortex. *Nature neuroscience*, *17*(12), 1661-1663.

65. Parr, T., Rees, G., & Friston, K. J. (2018). Computational neuropsychology and Bayesian inference. *Frontiers in human neuroscience*, 61.

66. Friston, K., Schwartenbeck, P., FitzGerald, T., Moutoussis, M., Behrens, T., & Dolan, R. J. (2014). The anatomy of choice: dopamine and decision-making. *Philosophical Transactions of the Royal Society B: Biological Sciences*, *369*(1655), 20130481.

67. O'Reilly, R. C., & Frank, M. J. (2006). Making working memory work: a computational model of learning in the prefrontal cortex and basal ganglia. *Neural computation*, *18*(2), 283-328.

68. Manganelli, F., Vitale, C., Santangelo, G., Pisciotta, C., Iodice, R., Cozzolino, A., ... & Santoro, L. (2009). Functional involvement of central cholinergic circuits and visual hallucinations in Parkinson's disease. *Brain*, *132*(9), 2350-2355.

69. Beliveau, V., Ganz, M., Feng, L., Ozenne, B., Højgaard, L., Fisher, P. M., ... & Knudsen, G. M. (2017). A high-resolution in vivo atlas of the human brain's serotonin system. *Journal of Neuroscience*, *37*(1), 120-128.

70. Yasue, I., Matsunaga, S., Kishi, T., Fujita, K., & Iwata, N. (2016). Serotonin 2A receptor inverse agonist as a treatment for Parkinson's disease psychosis: a systematic review and meta-analysis of serotonin 2A receptor negative modulators. *Journal of Alzheimer's Disease*, *50*(3), 733-740.

71. Moreno, J. L., Miranda-Azpiazu, P., García-Bea, A., Younkin, J., Cui, M., Kozlenkov, A., ... & González-Maeso, J. (2016). Allosteric signaling through an mGlu2 and 5-HT2A heteromeric receptor complex and its potential contribution to schizophrenia. *Science signaling*, *9*(410), ra5-ra5.

For Review Only

72. Mehta, M. A., Schmechtig, A., Kotoula, V., McColm, J., Jackson, K., Brittain, C., ... & Schwarz, A. J. (2018). Group II metabotropic glutamate receptor agonist prodrugs LY2979165 and LY2140023 attenuate the functional imaging response to ketamine in healthy subjects. *Psychopharmacology*, *235*, 1875-1886.

73. Romeo, B., Willaime, L., Rari, E., Benyamina, A., & Martelli, C. (2023). Efficacy of 5-HT2A antagonists on negative symptoms in patients with schizophrenia: a meta-analysis. *Psychiatry Research*, 115104.

74. Stefanics,G.,andCzigler,I.(2012).Automatic prediction errorr esponse to hands with un expected laterality: an electrophysiological study. *Neuroimage* 63, 253–261. doi:10.1016/j.neuroimage.2012.06.068

75. *Liégeois-Chauvel C, Musolino A, Badier JM, Marquis P, Chauvel P. Evoked potentials recorded from the auditory cortex in man: evaluation and topography of the middle latency components. Electroencephalography Clin Neurophysiol (1994)*

76. Näätänen, R. (1995). The mismatch negativity: a powerful tool for cognitive neuroscience. *Ear and hearing*, *16*(1), 6-18.

77. Rinne, T., Degerman, A., & Alho, K. (2005). Superior temporal and inferior frontal cortices are activated by infrequent sound duration decrements: an fMRI study. *Neuroimage*, *26*(1), 66-72.

For Review Only

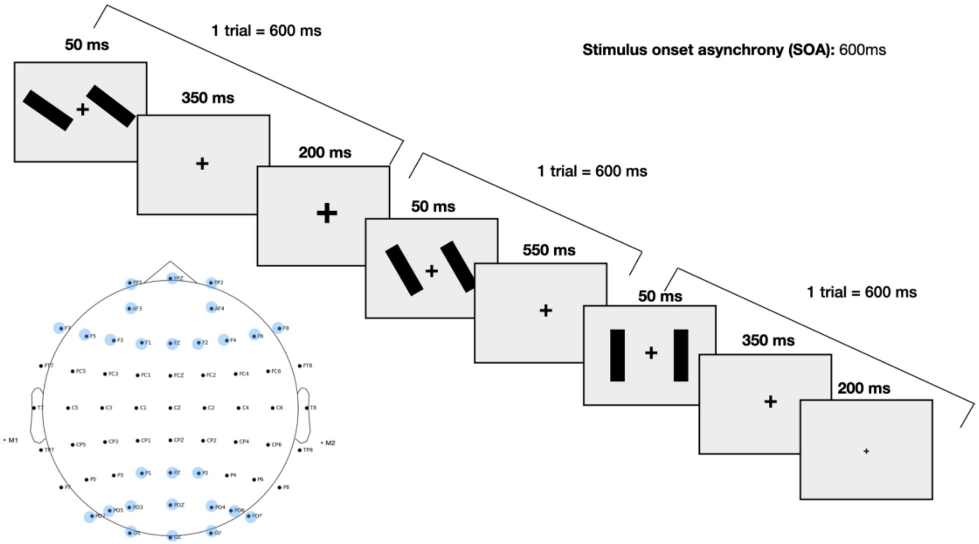

Figure 1. vMMN task sequence of events and channels of interest in our analyses.

677x381mm (72 x 72 DPI)

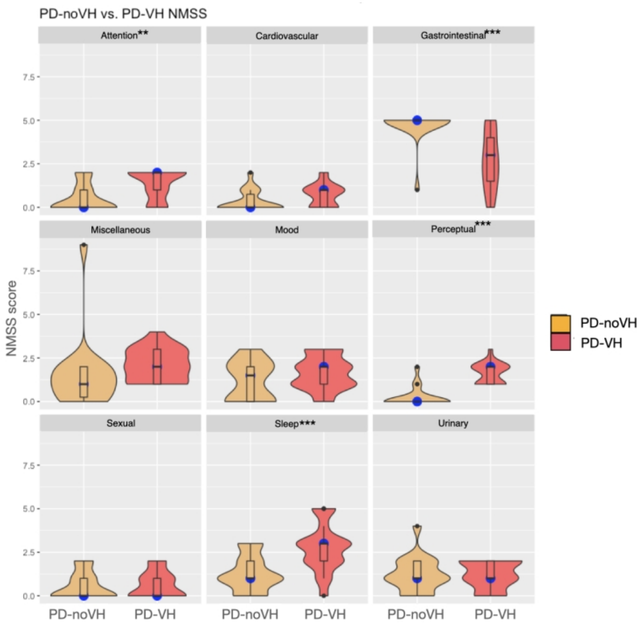

Figure 2. NMSS by symptom cluster. ** p <.005, *** p =<.001 Median (blue dot) and interquartile range (black bar in the center of each plot) are represented in the figure.

677x423mm (72 x 72 DPI)

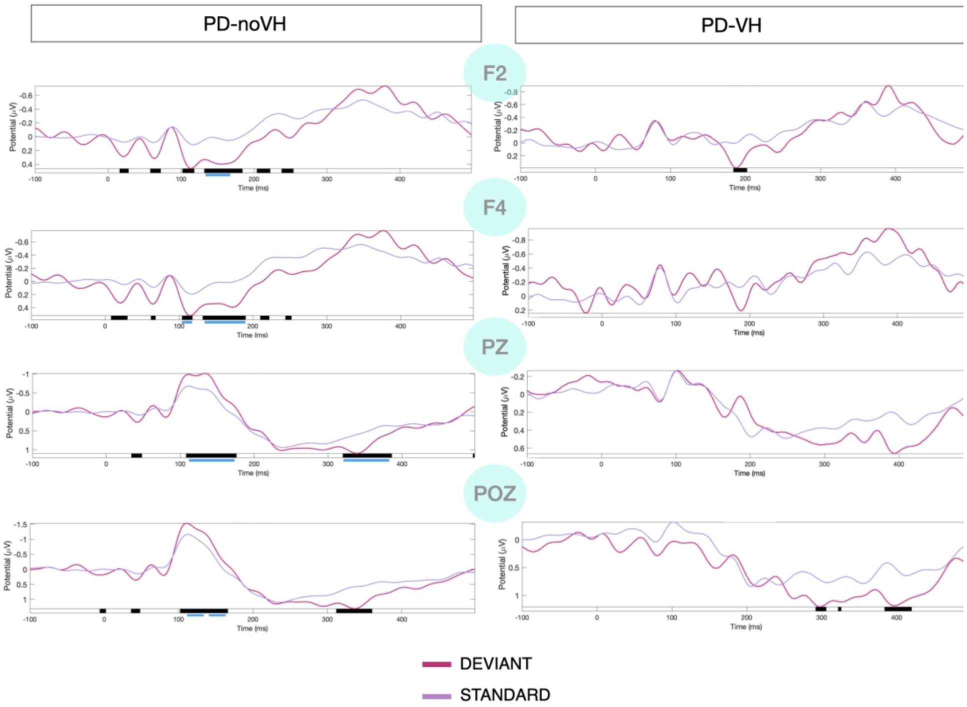

Figure 3. Standard (violet) and deviant (pink) waveforms in PD-noVH and PD-VH in the within group analysis. The black bars indicate the latencies at which the two waveforms significantly differ ($p < .05$). The blue lines indicate the latencies at which such differences survive multiple comparisons correction ($pFDR < .05$).

677x705mm (72 x 72 DPI)

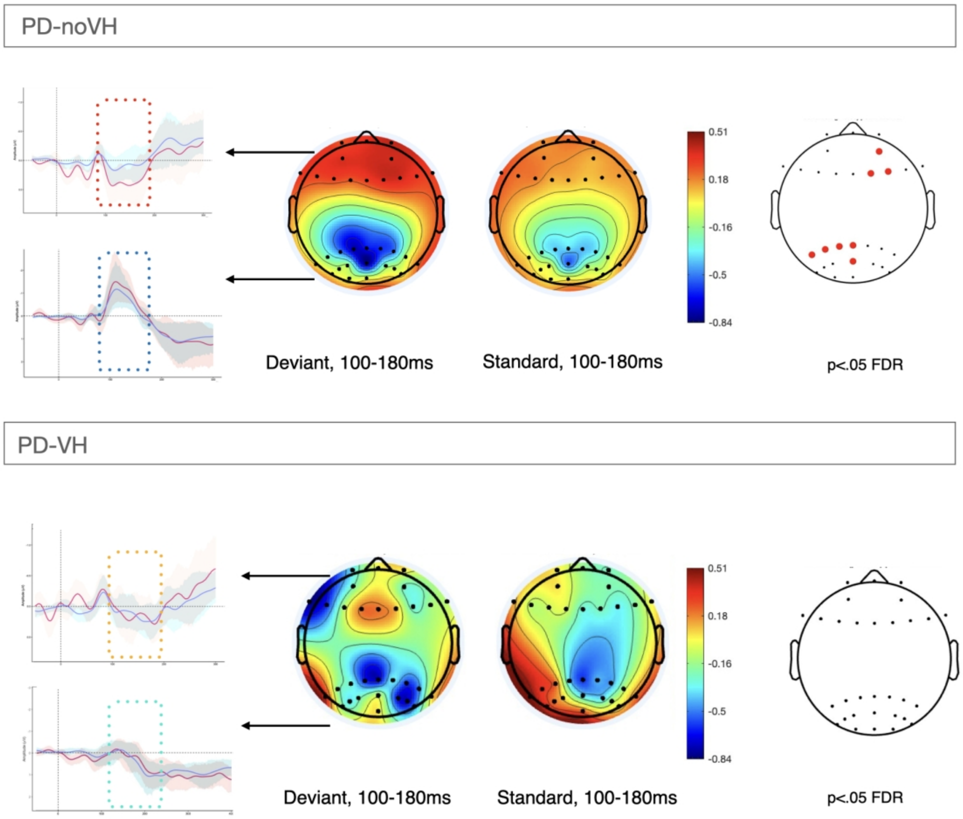

Figure 4. Within group scalp topography in the 100-180ms (EEGlab). Top row: scalp topography for PD-noVH. The channels represented with a red dot are those where a significant difference between standard and deviant (deviant was more negative posteriorly or more positive frontally) was found. Bottom row: the same analysis was used for PD-VH patients. The lateral insets show (clearly in PD-noVH) the parieto-occipital negative and frontal positive pattern we observed in the vMMN analysis (plots created with ggplot2 in R).

677x705mm (72 x 72 DPI)

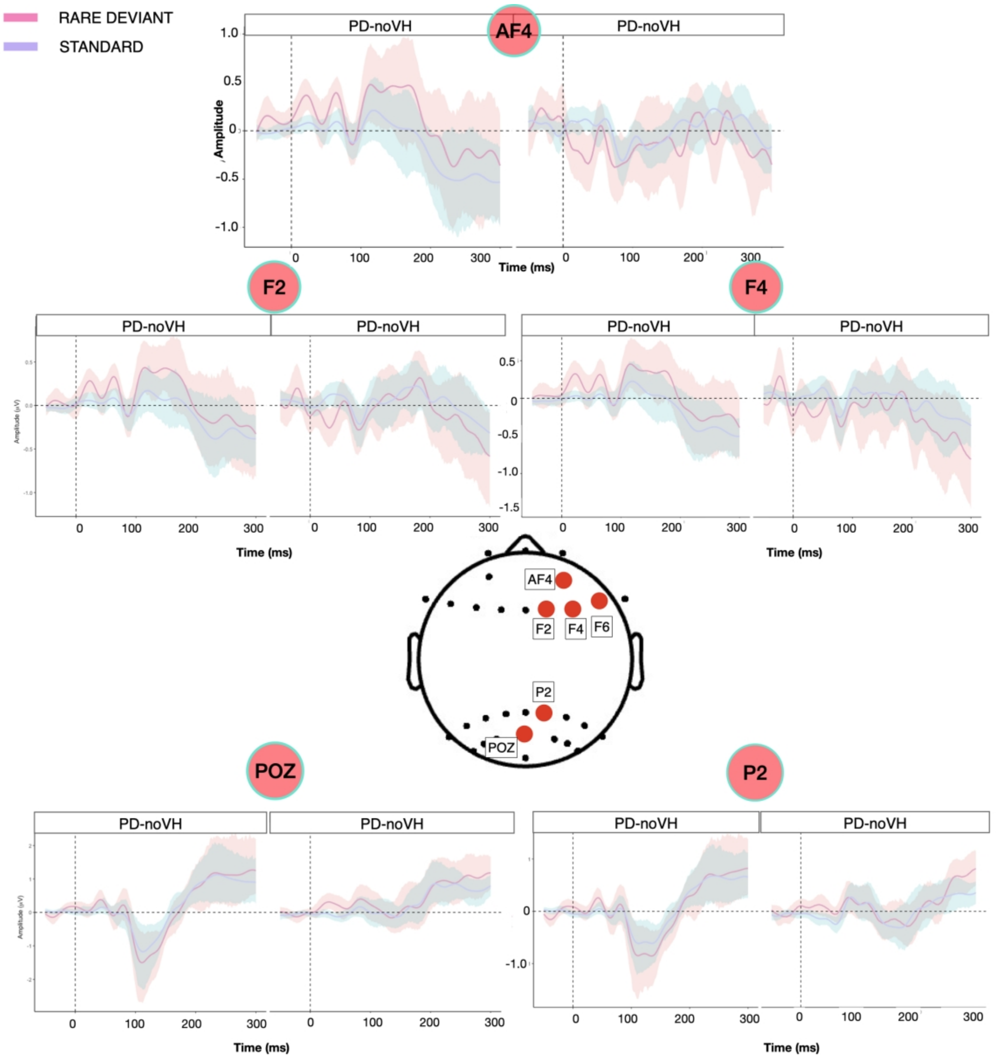

Figure 5. Electrodes (red dots) that presented significant deviant-standard vMMN amplitude differences in the groups (p <.05, corrected for multiple comparisons with the Benjamini-Hochberg correction). Electrodes pictured in red on the map: POZ, P2, F2, F4, F6, AF4. F4, F6, F7, F5, P1, P2, P3, P4, PZ, POZ. Standard (violet) and deviant (pink) waveforms for PD-VH and PD-noVH. The shaded area represents the variance.

705x740mm (72 x 72 DPI)

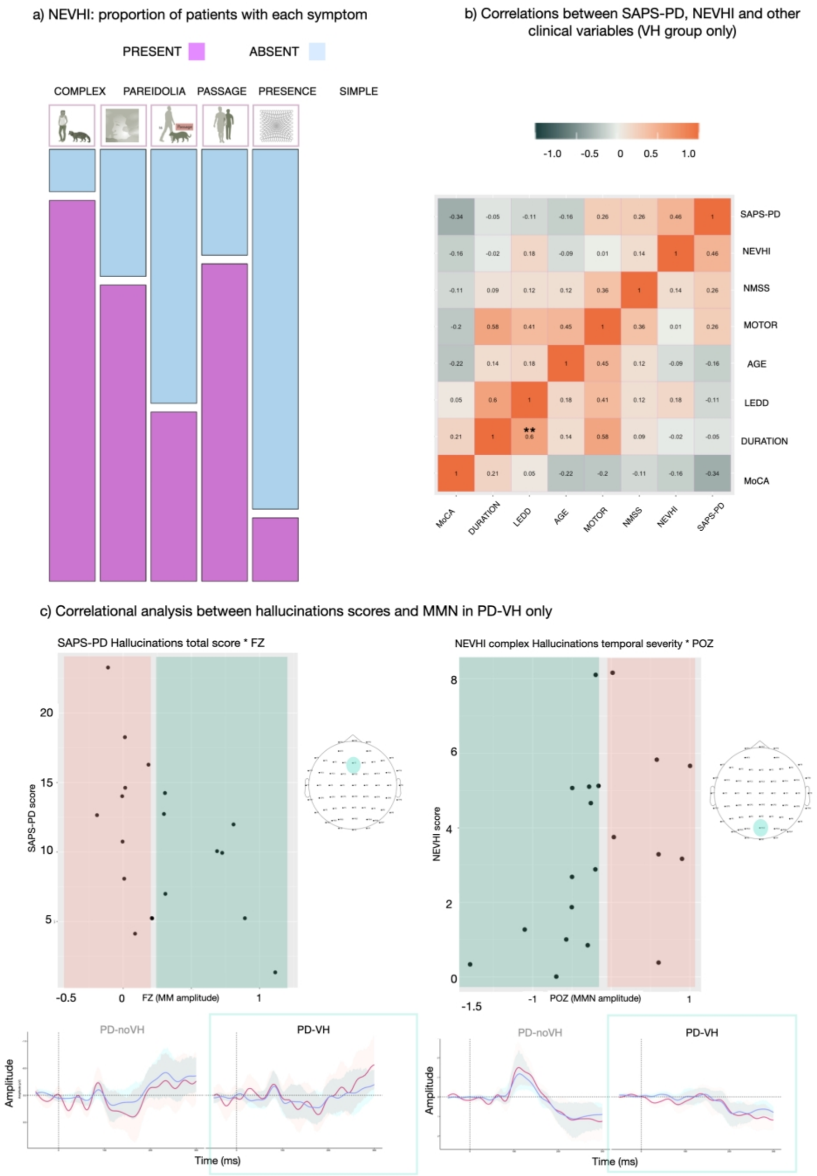

Figure 6. a) Proportion of patients with a specific type of visual hallucinations as detected by the NEVHI interview. The figures used to represent the symptom categories are in part derived from ffytche et al., 2017; b) Pearson’s product moment correlation coefficients for the clinical variables and neuropsychiatric assessments (PD-VH only) c) Left: SAPS-PD scores as a function of mismatch positivity and negativity at FZ after considering the sign of the mismatch between standard and deviant. Right: NEVHI temporal severity for complex VH. as a function of mismatch positivity POZ. The shaded green area represents those values that correspond to a normal deviant-standard difference, negative in the case of POZ and in this study positive for FZ. The red shaded area contains the deviant-standard values whereby the standard was greater than the deviant or there was no difference.

599x881mm (72 x 72 DPI)

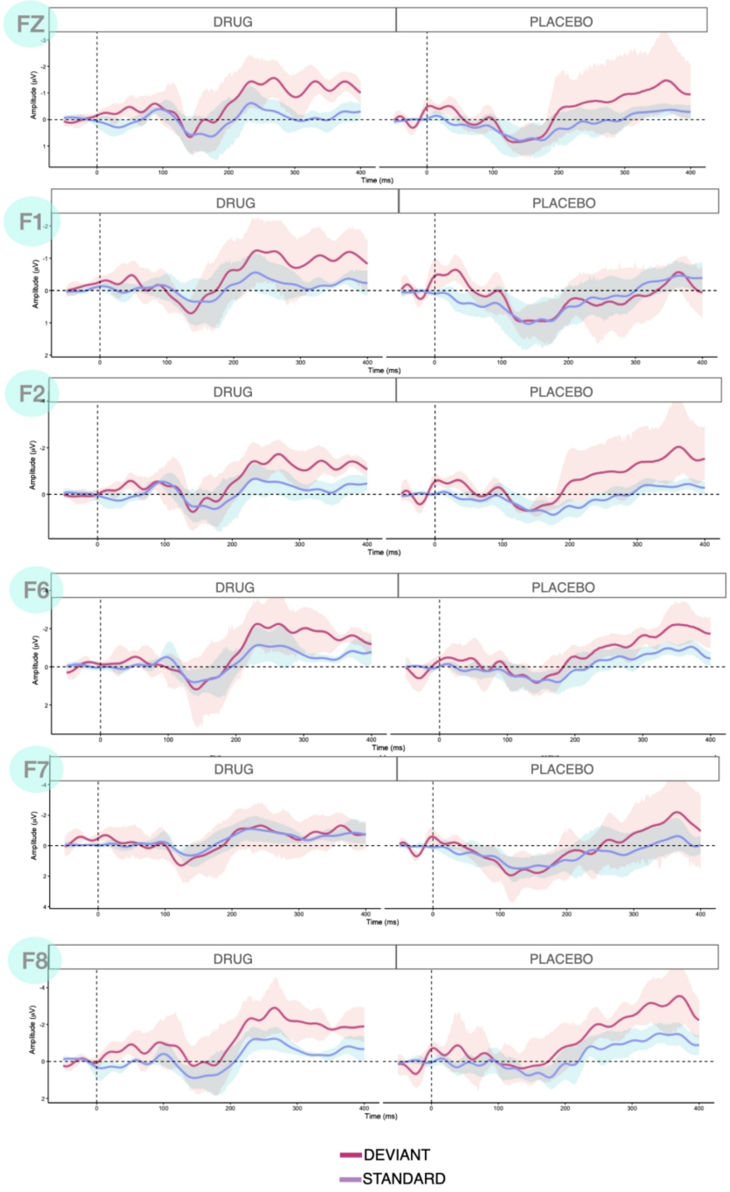

Figure 7. Left column: frontal electrodes in the drug arm, Right column: frontal electrodes in the placebo arm. Deviant is shown in pink, standard in violet. The difference between the drug and placebo was captured in a comparison of individual peaks, described in the text; individual waveforms are displayed in SI10.

599x881mm (72 x 72 DPI)

	Group	Mean	St. dev	p
Age	PD-NOVH	65.74	10.42	0.320
	PD-VH	68.85	8.60	
Disease duration	PD-NOVH	4.57	2.84	0.125
	PD-VH	6.28	3.73	
LEDD	PD-NOVH	329.78	268.33	0.198
	PD-VH	454.13	311.67	
MoCA	PD-NOVH	27.17	2.79	0.934
	PD-VH	27.10	2.79	
*SCOPA-motor**	PD-NOVH	17.88	7.29	0.310
	PD-VH	20.72	9.14	
NMSS	PD-NOVH	7.47	3.48	<.001
	PD-VH	14.44	3.33	
CISI-PD	PD-NOVH	6.35	3.28	0.071
	PD-VH	8.5	3.78	
CISI-PD motor	PD-NOVH	2.47	0.94	0.79
	PD-VH	2.55	0.83	
CISI-PD motor complications	PD-NOVH	1.29	1.36	0.29
	PD-VH	1.79	1.40	
CISI-PD cognitive	PD-NOVH	0.65	0.86	0.02
	PD-VH	1.60	1.35	
CISI-PD disability	PD-NOVH	1.94	0.90	0.05
	PD-VH	2.60	1.05	

Table1. *Participants demographics and clinical information. We report disease duration (in years), LEDD (levodopa equivalent dose), Montreal Cognitive assessment (MoCA) scores (participants were not included if they presented a score lower to 22), SCOPA-motor* total score, NMSS (non-motor symptom scale) total score, Clinical Impression of Severity Index for PD, completed by a Parkinson's neurologist upon physical exam and the breakdown of the CISI-PD in the four categories (motor severity, motor complications, cognitive status and disability; cognitive status and disability are computed based on the self-reported deficits of the patients). *One PD-noVH participant did not have a SCOPA-motor or UPDRS-III score and was excluded from this comparison*

Only

Visual Mismatch Negativity Signatures in Parkinson's disease Visual Hallucinations

Supplementary Information

Supplementary Information 1. *Study questionnaires details* 2

Supplementary Information 2. *Behavioural task pilot* 5

Supplementary Information 3. *Behavioural task PD-noVH and PD-VH patients* 9

Supplementary Information 4. *EEG procedure* 10

Supplementary Information 5. *EEG pilots* 12

Supplementary Information 6. *Drug study design and procedure* 12

Supplementary Information 7. *Parietal channels early vMMN* 13

Supplementary Information 8. *EEGlab studies* 17

Supplementary Information 9. *Clinical scores correlations in PD-VH* 22

Supplementary Information 10. *PD-VH drug additional information* 22

For Review Only

Supplementary information 1. *Details about the assessments and questionnaires used to investigate patients motor symptoms and their hallucinations and other non-motor symptoms.*

Participants underwent a phone pre-screening and attended one study day at the clinical research facility (CRF) of King's College Hospital (London, UK) where they completed screening, for which we collected medical history, medication, physical and non-motor examinations (NMSS, Chaudhuri et al., 2007), PD-specific exams to assess disease severity (Clinical Impression of Severity Index (CISI-PD), Martinez-Marin et al., 2006), motor examination (SCOPA-MOTOR) and conducted the EEG session. We also conducted a baseline cognitive assessment comprising the Montreal Cognitive Assessment (MoCA), verbal and semantic fluency, months reversed and the Wechsler Test of Adult Reading (WTAR) for estimation of pre-morbid ability (Whitney et al., 2010). Inclusion criteria included MoCA above 21 and NPI>4 or recurrent visual hallucinations as recorded with other questionnaires (see below and SI1) if there was no study partner. Exclusion criteria included ongoing disabilities, medical history and medical conditions that could interfere with the study conduct or clinical assessments; this included current psychiatric diagnosis (except psychosis in the PD-VH group) as this could interfere with the EEG task results and severe motor impairment judged to make the study days too challenging for participants. People on medication potentially interfering with study measurements were excluded on a case-by-case basis.

The Non motor symptoms scale (NMSS) is a nine-domain questionnaire consisting of 30 questions designed to test the frequency and severity of the non-motor symptoms of Parkinson's disease over the past month (Chaudhuri and Martinez-Martin, 2008).

Table SI1a. *NMSS domains details.*

Domain	Group	Mean	Std. Dev	*F*	*p*
**Gastrointestinal**	PD-noVH	4.78	0.94	21.74	<.001
	PD-VH	2.68	1.67		
**Perceptual**	PD-noVH	0.22	0.55	55.72	<.001
	PD-VH	1.63	0.60		
**Attention**	PD-noVH	0.50	0.79	10.64	0.002
	PD-VH	1.37	0.83		
**Sleep**	PD-noVH	1.28	1.02	13.43	0.001
	PD-VH	2.74	1.37		

To investigate visual hallucinations, study partners completed the hallucinations and delusions scale of the Neuropsychiatric Inventory (Cummings et al., 1994). The Neuropsychiatric inventory (NPI) was administered to the study partner as standard procedure and assesses neuropsychiatric symptoms. It consists of 12 items, with section A (delusions) and B (hallucinations) exploring the domains of these symptoms. If symptoms are present, questions about frequency (1-3) and severity (1-4) are collected

and multiplied, and hallucinations and delusions are added up to get the NPI total score Cummings et al., 1994).

PD-VH participants were administered the Scale for the Assessment of Positive Symptoms-PD (SAPS-PD) adapted for PD from schizophrenia (Voss et al., 2013) and an adaptation of the NEVHI to assess the phenomenology of visual hallucinations (D'Antonio et al., 2022).

The Scale for the Assessment of Positive Symptoms (SAPS) originally designed for schizophrenia has been found to be reliable for people with Parkinson's disease (Fernandez et al., 2008). The SAPS-PD is composed of the 9 items found to be most frequently reported in patients with PD psychosis (Voss et al., 2013). In the article, we only used scores for hallucinations in these patients. Only one patient presented delusions, and they retained insight about it. SAPS-PD scores reported in the article range from 2 to 20. The hallucinations scale of the SAPS-PD counts 5 items: auditory hallucinations, voices conversing, visual hallucinations, somatic/tactile hallucinations, global rating of hallucinations. The maximum score for each item is five, for a total score of the hallucinations scale reaching a maximum of 25. Participants with >10 had also auditory or somatic hallucinations.

For Review Only

The NEVHI is a visual complaints questionnaire that is used to assess the phenomenology of visual hallucinations (D'Antoni et al., 2022). The scale includes 6 questions assessing different types of VH: complex hallucinations, pareidolia (a specific type of visual illusion), presence, passage, simple hallucinations. The last question 'other' is designed to pick up any other visual experience and classified under the relevant section. The NEVHI also provides a score for distress, and information about insight and delusions specific to the type of VH, differing from the general delusions assessed by SAPS-PD. Participants' scores at the different VH types are also reported in **table S1b**.

The adapted version of the NEVHI has no scoring rules, so we computed temporal severity by multiplying duration and the frequency of the VH, using an ordinal scale following as closely as possible D'Antonio et al., 2022. We also computed a continuous temporal severity score multiplying the raw number of minutes spent hallucinating by the raw number of VH in a month. We computed temporal severity as a continuous measure and as an ordinal measure, following the method used by D'Antoni et al., 2022, as two patients constituted outliers as they hallucinated 'continuously' or 'for hours', whereas the rest of the patients had hallucinations lasting seconds or minutes. Thus to be able to carry out correlational analyses retaining all the patients but taking this into account, we used an ordinal scale as in D'Antoni et al. for duration, where seconds =1, minutes =2, hours =3 and continuously =4. For frequency, as our new version of the NEVHI did not have an ordinal scale to rate this, but asked participants to state how many VH in a month they had, we adapted the ordinal scale reported in the article cited above. **Table S1c** summarises how we computed the temporal severity score, using the raw data from the complex VH question as an example.

	Complex	Pareidolia	Presence	Passage	Simple
N	17	14	15	8	3
%	85	70	75	40	15
Duration (sec)	1119*	1296*	1243*	8	4841.66*
Frequency(vh/month)	29.5	23.50	20.39	25.58	6.17
Distress	0.5 (2)	2.23 (4)	0.73 (4)	0	0.7(1)
Frustration	1.25 (6)	1.93 (4)	1.53 (5)	0	0
Insight***	0.07% (1)	0.14% (2)	1.46% (5)	0	33% (1)
Delusion***	0.1% (2)	0.07% (1)	0.13v (2)	12.5%(1)	0

Supplementary Table S1b. *NEVHI scores reported per scale. Duration is reported in seconds, frequency in how many VH are experienced in a typical month.*

Average scores are reported for distress and frustration ratings (rated on a scale from 0 to 10), in brackets we report how many participants had the symptom.

% of participants for insights and delusions are reported: we included in the calculations those who had a certainty the VH was real at the time they occurred; 2 more participants stated it might happen at times.

*one participant said they had one of the VH type continuously; in order to put a number in seconds we assigned 18000 seconds to their VH

***Insight: some patients said that for the reported VH type it took them a few seconds to realise the experience is indeed a VH; delusion: 2 of the patients report VH-specific delusions in the sense that they responded 'sometimes' to the questions asking if they had a different explanation for their VH.

For Review Only

Table SI1c – *The table shows how from raw scores of duration (seconds) and frequency (how many experiences in a month) have been converted to ordinal scores and in the multiplicative factor that we used as a measure of temporal severity.*

Duration (sec	Ordinal_duration	N. of VH per month	Ordinal category assigned	Ordinal_frequency	Severity (duration*freq)
30	1	3	every few weeks	1	1
2.5	1	12	more than once a week	3	3
5	1	15	more than once a week	3	3
0	0	0	Not reported	0	0
0	0	0	Not reported	0	0

1	1	40	more than once a day	5	5
900	2	4	every few weeks	1	2
10	1	2.5	every few weeks	1	1
2	1	50	more than once a day	5	5
5	1	60	more than once a day	5	5
0	0	0	Not reported	0	0
5	1	2	every few weeks	1	1
5	1	21	daily/almost daily	4	4
5	1	8	more than once a week	3	3
5	1	55	more than once a day	5	5
5	1	90	every few hours	6	6
2	1	12	more than once a week	3	3
continuously (18000)	4	2 to 6	once a week	2	8
60	2	25	daily/almost daily	4	8
5.5	1	110	every few hours	6	6

Supplementary Information 2. *Behavioural task pilot with healthy participants.*

The EEG task administered to PD patients was programmed in the software compatible with the EEG Neuroscan computer at the research facility *(Presentation v.17.2)* with stimuli presented in a pseudo-randomized manner, with details reported in the Methods in the main text. In the online behavioural pilot, we used the same stimuli and timings used in the EEG task, but the bars showed on the screen were blue with a white coloured background. whereas in the visual task shown during the EEG session the bars were black with a light grey background and a dim light, for participants' comfort as the light grey background was easier on the eyes.

The peripheral stimuli aimed at eliciting the vMMN were symmetrical flashing bars, with the variation being in orientation: bars were presented with a 0 (rare deviant), 30 (frequent deviant) and 60 (standard) degree orientation (see **SI2** for details). The bars for each orientation were simultaneously presented in the left and right peripheral visual field (offset 400/-400) centred on the horizontal meridian, staying on the screen for 50ms. The cross changes had a duration of 200ms. The stimulus onset asynchrony (SOA) was 600ms.

Healthy participants Pilot. Ninety-four healthy volunteers participated in the pilot study (64 F, 30 M, age range 25-73 years, mean age 41.1 years, SD 11.4), with 66 (28 drop out) completed the online visual task. Participants had normal or corrected to normal vision. The volunteers signed written informed

consent before participating in the study. The online task was designed with the online experiment platform Gorilla (Anwyl-Irvine et al., 2020).

Participants were instructed to pay attention at fixation cross at the centre of the screen and to ignore any peripheral stimuli. They were instructed to press the left or the right button of their keyboard when the cross became bigger or smaller.

The peripheral stimuli were flashing bars that should elicit the MMN in the EEG task. The standard orientation of the bars had a target prevalence of 64%, the frequent deviant of 24% and rare deviant of 12%. In the pilot study participants were divided into three groups of 22, each group with a different visual configuration of the standard orientation (30°- 60°- 90°) to determine whether this influenced the salience of the peripheral stimuli or waveform of the visual evoked potential. At the end of the task, participants were also asked to report the most and least frequent orientation of the peripheral flashing bars presented in the task to investigate the extent to which they might have redirected their attention to the peripheral stimuli.For the EEG study, only one of the configurations of the visual task was shown (see figure S1 below). In order to select which condition to use a small pilot EEG study was carried out with healthy participants. Upon repeated testing with the three task configurations, we chose configuration 2, as it evoked the clearest visual evoked response but did not differ from the other configurations in terms of evoked waveforms.

The bar distance from the fixation cross was at a visual angle of approximately ± 9 °; the same angle was measured for the bar length; the bar width was at a ± 2 ° visual angle; for the screen width the visual angle was 27 ° and for screen height the angle was 21 °.

For Review Only

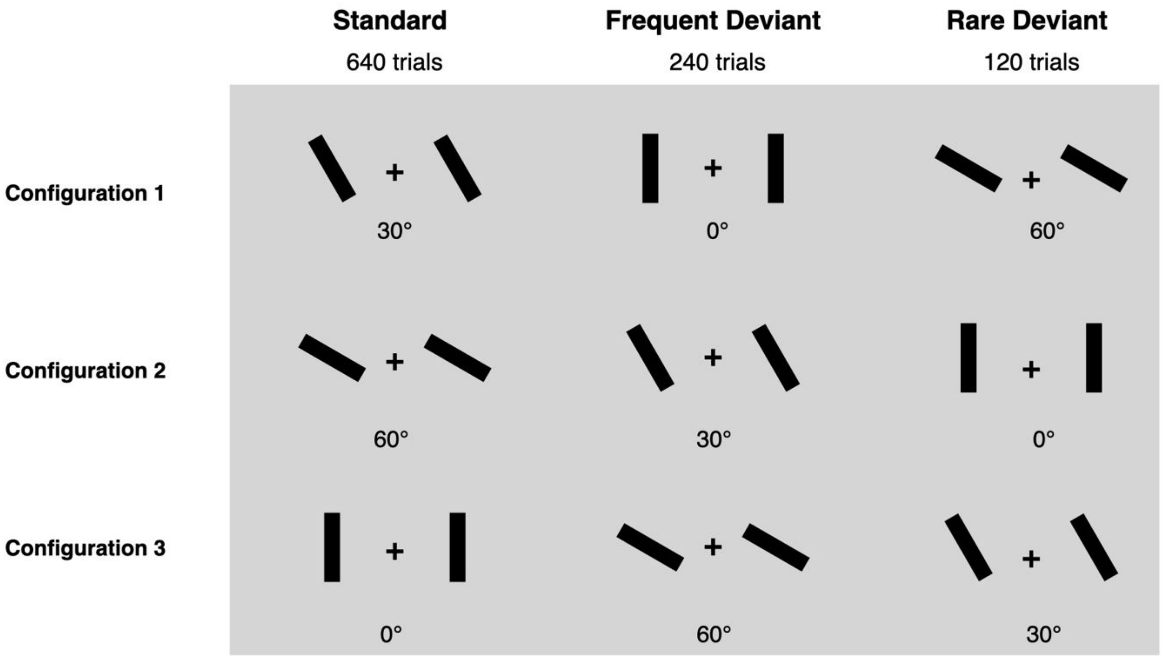

Figure S2a: The visual configurations of the flashing bars used in the pilot study with the target prevalence of the three orientations of the bars in the trials. Configuration 2 was selected for the study.

During the visual task, similar stimuli and instructions were provided to the participants of pilot and EEG studies. We included in the analysis of the orientation frequency responses, only participants that achieved 70% accuracy rate at the cross-change detection task (58, 8 excluded).The participants of both studies were able to discriminate the cross change displayed at the centre of the screen and their performance was associated with different types of errors: i) omission errors (no response for trial change); ii) commission errors outside trials (responses for no trial change) iii) response errors (incorrect response to the trial).

- Under configuration 1 (30 standard) the mean accuracy (%) to cross change was 84% (SD 24.9), with omission errors on 10% of the trials (10.1, SD 23.0), commission errors on 17% of the trials (16.1, SD 26.1) and response errors on 6% of the trials (6.10, SD 8.72)
- Under configuration 2 (60 standard) the mean accuracy (%) to cross change was 87% (SD 14.6), with omission errors on 6% of the trials (5.48, SD 9.09), commission errors on 60% of the trials (59.6, SD 170) and response errors on 7% of the trials (7.05 SD 12.5)
- Under configuration 3 (90 standard) the mean accuracy (%) to cross change was 93% (SD 14.0) with omission errors on 5% of the trials (5.19 SD 12.9), commission errors on 16% of the trials (16.1 SD 24.6) and response errors on 2% of the trials (2.38 SD 3.38).

Review Only

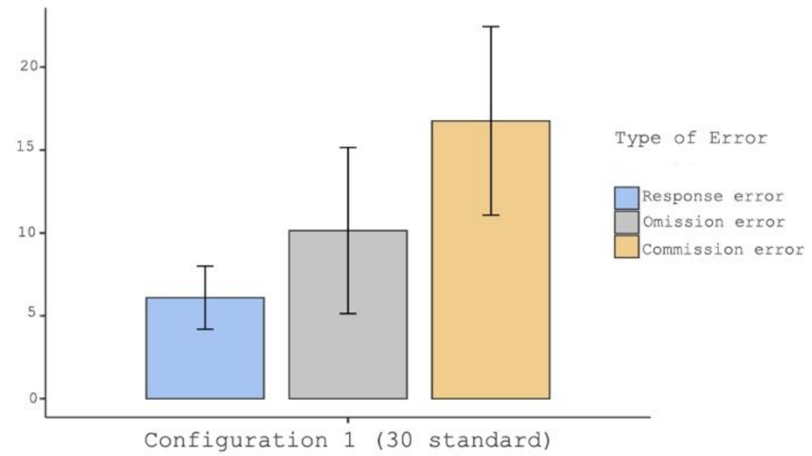

A

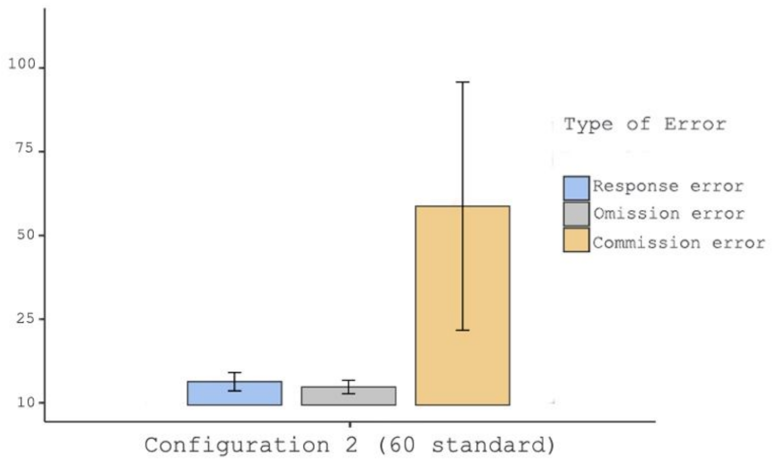

B

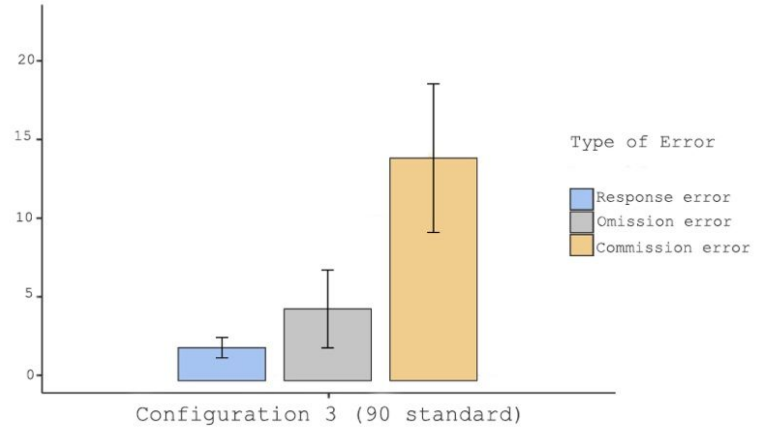

C

Figure S2b. Bar plots displayed the type of errors made with: A) Configuration 1; B) Configuration 2, C) Configuration 3.

The results of Chi Squared test shows a significant association between the three configurations of the visual task (30°,60°,90°) and the behavioural responses to the orientation frequency discrimination task

(Most frequent orientation: χ (4, N = 58) = 27.054, p= .000; Least frequent: χ (4, N = 58) = 19.828, p= .001). The association between the variables was not significant when only configurations 1 (standard 30°) and 2 (standard 60°) were included in the analysis (Most frequent orientation: χ (2, N = 38) =1.292, p= 0.524; Least frequent orientation: χ (2, N = 38) =3.474, p= 0.176). Therefore, the observed responses were significantly different from the expected responses when the configuration 3 (90° standard) was included in the analysis but not when only configuration 1 (30° standard) and 2 (60° standard) were included.

Supplementary Information 3. *Behavioural task with patients: PD vs. PD-VH.*

When comparing the two groups of patients at the cross-change detection task, patients with PD-VH performed worse than PD-noVH [F(1,34) = 10.64, p = .003]. For PD-noVH, the overall accuracy was 91.3% (mean =40.17 sd = 4.63) whereas for PD-noVH it was 70% (mean = 31.06 sd 10.90). PD-VH and PD-noVH did not differ in the amount of trials they missed however [F(1,34) = 2.86, p = .100].

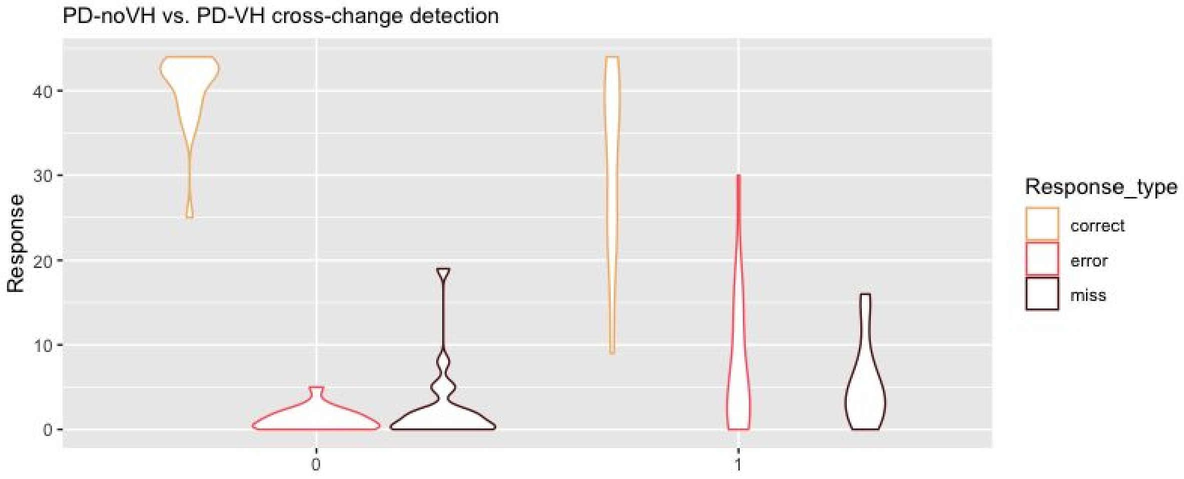

Figure S3. On the left panel PD-noVH, on the right PD-VH.

One PD-noVH and two PD-VH participants were excluded from the behavioural analysis. Inspection of their response outputs was necessary to detect that participants were paying attention to the task (responses given in correspondence to triggers); however these participants did not understand completely the instructions, and pressed the same key for all trials. We excluded these participants as we thought this would affect the results not allowing to investigate relevant differences.

Supplementary Information 4. *Description of the vMMN task and EEGlab preprocessing.*

During the EEG session, participants sat on a comfortable chair in a darkened, sound attenuated, and electrically shielded room, at a distance of 74-76 cm from the computer screen. Participants were fitted with a Compumedics Neuroscan 64-electrode EasyCap with sintered Ag-AgCl sensors and the SynAmps RT amplifier. We recorded vertical eye movements with two electrodes above and below the left eye, and horizontal eye movements with two electrodes at the outer canthi of the eyes, bilaterally. Signals were grounded to channel CZ, located at the midline. We also acquired signals from left and right mastoid (M1 and M2). Nevertheless, during the pre-processing, the signal was re-referenced to common average (see data analysis paragraph for details). Impedances of all electrodes were kept at 15kΩ or below. Signals were recorded using Scan 4.5 software. A sampling rate of 1000Hz was used for the recording. We used two EasyCaps for the study which were alternated across participants within each group.

Task. The task was a visual change detection task, adapted from Quian et al. 2014. Participants were given a joystick and were asked to pay attention to the fixation cross at the centre of the screen only and to press a button if the cross became smaller and another button if the cross became bigger. Participants were instructed to ignore the peripheral stimuli. The use of a concomitant visual task is recommended to minimize possible attentional effects in processing the stimuli designed to evoke the MMN and its relative simplicity was aimed to avoid exhausting participants (Stefanics et al., 2014). Participants attended a practice run first, to familiarise with the task, consisting of 50 initial training standard only trials (6 with cross change). Participants were allowed to retake the practice trials if they felt they were not completely confident in starting the task. The experimental task (1000 trials) was divided in 2 blocks with a total of 44 trials where they cross changed in size. A two-minutes break was given between the blocks to allow participants to rest their eyes. The visual task was presented on a CRT monitor in the soundproof recording room; the monitor was connected to the experimental computer in a separate control room. The CRT monitor was chosen for its phosphor temporal characteristics to guarantee the best possible synchronisation between the presentation of the stimuli from the control room computer and the CRT monitor, consistently with what found in the literature (Husain et al., 2009). Using an oscilloscope, we verified that the delay between trigger and light sensor with VGA to CRT was of 6ms only (with LED monitor 13ms). We inverted the colours for the test to obtain a clean black to white signal, to check that the CRT produced a very immediate image, switching to full brightness immediately, while the LCD response ramped up in brightness. We also took a slow-motion video of the task running on the CRT monitor to check that it was truly v-synced, and we failed to see any partial images being drawn to the screen, which is a sign of good syncing.

The EEG task was programmed with *Presentation* with stimuli presented in a pseudo-randomized manner as the trial list was generated with a MATLAB script designed to create the task list according to the Poisson distribution. As it is required to elicit proper MMN and to avoid confounding processes being elicited, the stimuli differed only in the feature carrying the deviant information, thus orientation.

For Review Only

The visual stimuli presented in the task were: a black cross (*Presentation* units height = 90; width = 9) positioned at the centre of the screen that became briefly smaller (*Presentation* units height = 45; width = 4.5) or bigger (*Presentation* units height = 180; width = 18), At the periphery, two flashing bars (*Presentation* units height = 450; width = 100), were presented with a 0 (rare deviant), 30 (frequent deviant) and 60 (standard) degree orientation. The bars for each orientation were simultaneously presented from a distance from the centre of the screen/cross of 400/-400 *Presentation* units, staying on screen for 50ms. The cross changes had a duration of 200ms. The stimulus onset asynchrony (SOA) was 600ms. To control for potential ERP differences related to the differences in the visual stimuli, we decided not to change the probabilities of standard and deviant stimuli across experimental blocks in order to keep the task under 15 minutes, as it would have been fatiguing for the patients. As the sample size did not allow to counterbalance the task across participants, we ran pilot sessions to make sure that the waveforms elicited by the standard and deviant stimuli did not differ (**Supplementary Information 5**) and then we picked one of the possible combinations as our task.

EEGlab preprocessing pipeline. For each participant, we removed VEO and HEO eye channels before high-pass filtering (lower edge of 0.1Hz and a higher edge of 30 Hz) to minimise the introduction of artefacts and to remove high-frequency noise. Data was re-referenced to the common average after this step. We used the *runica* algorithm to run independent component analysis on the data. After checking the ICA components with 2D component activations, maps and spectra, we removed those components that included eye blinks or motion artefacts. Data was once again re-referenced to the common average. After this step, an event list was created to divide the recording in standard, rare deviant and frequent deviant events. Data was epoched from 100 ms pre-stimulus to 500 ms post-stimulus onset and epochs were manually inspected. We rejected trials when the cross changed in size, when participants hit the joystick button, and bad epochs were rejected when necessary. The epochs were averaged separately for standard and deviant stimuli in the three different conditions to produce the final averaged ERPs. This procedure was done separately for frontal (FPZ, FZ, F1:F8), parieto-occipital electrodes (OZ, O1, O2, PO1:PO8) and parietal electrodes (PZ, P1:P8). We exported MATLAB waveform plots to access peak amplitude data, together with .txt and ERP files for quantitative MMN data analysis. In addition, we divided the recordings in standard and rare deviant to be entered as datasets for an EEGlab study of the two different ERP components in PD vs. PD-VH as an additional way to analyse the differences between standard and deviant stimuli in the two groups.

For Review Only

Supplementary Information and Figure 5. *EEG pilots to ensure MMN was being measured in each possible condition and not a stimulus-specific waveform.* The deviant is represente in pink and the standard in violet, as in the plots in the main text.

Participants were: for Condition 1, P1 age = 28 student; Condition 2, P2 male age = 32, MS student; condition 3, P3 age = 24 university student. Participants 1-3 had normal vision; Participant 2 had corrected to normal vision.

The deviant is the pink line, the standard is the violet line.

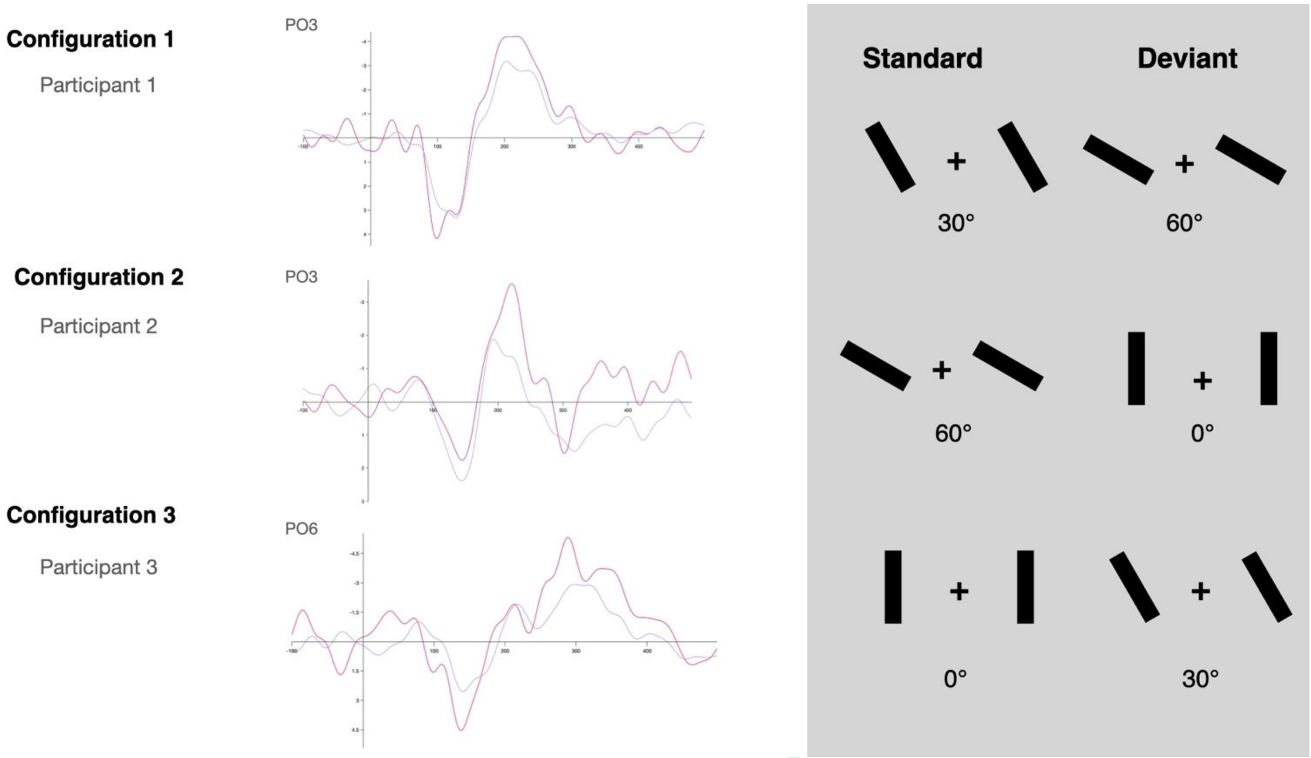

Supplementary Information 6. *Saracatinib and psylocibin behavioural study in healthy volunteers.*

One group of 23 healthy male volunteers participated in the study. As there was no information available on the effects of psilocybin and saracatinib on the developing foetus we took a conservative approach in excluding females. Participants were excluded if they had a personal history of psychiatric illness (assessed through a structured interview); first-order relative with a history of psychotic illness; evidence of cardiac, hepatic, renal, gastrointestinal or neurological disorders; excessive use of caffeine (> six cups of coffee per day or equivalent) and alcohol (> 28 units per week); current use of medication

and failure of drugs of abuse test at screening or on a study day using a 10-panel test. Only participants with previous experience of hallucinogenic drugs were included in this study. No participant was a current regular user.

Three participants did not complete the study: the QTc reading of one participant's ECG exceeded the upper limit specified in the protocol on the day of testing; one participant experienced high anxiety prior to the psilocybin dosing on his first session and withdrew from the study (was in placebo arm) and one participant tested positive for cocaine on the morning of his second session. As such, 20 participants completed the study (mean age 26.6, SD 7.1, range 19 – 47).

This was a double-blind, placebo-controlled, cross-over, counter-balanced design for saracatinib. The drug is a SRC kinase inhibitor. SRC is a tyrosine kinase that plays a role in glucose metabolism regulation in cancer cells and had been used as a candidate therapeutic target in patients with solid tumours. Here we had evidence for brain penetration from rodent CSF after a single dose (AZ in-house data), although subsequently it was shown multiple days of dosing produces clear exposure and measurable effect on peripheral SRC kinase inhibition (Nygaard et al. 2015).

Eligible, consented participants attended two study days at least one week apart (mean 13.3 days, SD 3.5, range 7 – 15). Participants were randomised to receive an oral placebo followed by psilocybin or oral saracatinib 125mg followed by psilocybin. At approximately 240 minutes post-oral drug dose participants entered the scanner. The scanning session lasted 90 minutes, with a pump-controlled infusion of 2mg psilocybin in 10mL saline (prepared as a suspension and infused through a sterile filter) over 2 minutes occurring approximately 40 minutes into the scanning session.

The psychedelic experience was recorded using the top ten items from the questionnaire taken from Carhart-Harris et al. (2011), with the principle question of interest being "How intense were the drug effects when at their peak." One-tailed paired sample t-tests were used to analyse the data.

Supplementary Information 7. *PD-VH only saracatinib pilot study details and procedures.*

Participants. Out of 42 potential participants identified, 25 met our main criteria and were phone-screened. Of them, 11 were eligible for the drug trial and 7 patients passed the screening and were enrolled in the study, with 5 completers. Of the 7 patients enrolled, one patient had to be excluded from the analysis due to an incidental finding in the MRI scan. Another patient completed one arm of the study but before they could start the second arm the pandemic and the first lockdown occurred and by the time they could re-enter the study, the disease had progressed too quickly to make it safe. The initial recruitment target was 30 patients with 26 completers, however the study was paused twice during the

For Review Only

pandemic, affecting subsequent recruitment, with patients also not feeling immediately up for an on-site 5 visit ~2 months study. At the end of 2021, the drug stopped being manufactured, thus we stopped recruiting for the drug study, but the patients that were lined up for screening joined the non-drug arm of the study (Study 1). (More on this in the *Limitations* section).

Inclusion Criteria (criteria 1-7 were shared with the no-drug study)

To be eligible for participation in this study, the subject must:

1. Understand the study procedures and agree to participate by providing written informed consent.
2. Have a confirmed diagnosis of Parkinson's disease using internationally accepted UK brain bank criteria.
3. Be male or female
4. Be right handed
5. Aged 40 years or over
6. Have a score of at least 22 on the Montreal Cognitive Assessment (MoCA).
7. Have a diagnosis of idiopathic PD with moderate severity
8. Be judged to be in good health by the investigator, based on clinical evaluations including laboratory safety tests, medical history, physical examination, 12 lead ECG and vital signs measurements performed at screening and prior to administration of the initial dose of study drug.
9. Have a combined score of at least 6 or an individual score of at least 4 on the neuropsychiatric inventory (NPI [20]) 23 items A (delusions) and/or B (hallucinations).

Exclusion Criteria (criteria 1-9 were shared with the no-drug study)

The subject must be excluded from participating in the study if the subject:

1. Has an ongoing disability, medical or neurological history, cognitive impairment, or conditions that in the opinion of the investigator may interfere with study conduct or clinical assessments.
2. Has a hairstyle which would affect EEG recording.
3. Has a history of regular alcohol consumption exceeding 14 units/week (6 glasses of 13.0% wine (175ml), 6 pints of 4.0% lager or ale (568ml), 5 pints of 4.5% cider (568 ml) or 14 glasses of 10.0% spirits (25ml)) within 6 months of screening.
4. Uses tobacco- or nicotine-containing products in excess of the equivalent of 5 cigarettes per day.
5. Uses caffeine containing products of the equivalence of 5 cups of regular filter coffee per day
6. Is unwilling or unable to comply with the Lifestyle guidelines.
7. Has, in the opinion of the investigator, any medical or psychological condition or social circumstances which would impair their ability to participate reliably in the study, or who may increase the risk to themselves or others by participating.

For Review Only

8. Known to have tested positive for human immunodeficiency virus.

9. Participation in another clinical study with an investigational product administered in the last 3 months for the PDP cohort and 1 month for the PD cohort

10. Is a female of child bearing potential

11. Is currently taking anticholinergic medication.

12. Is currently taking any medication known to be a moderate or potent CYP3A4 inducer or inhibitor.

13. Refuses to be withdrawn from quetiapine.

14. Has a family history of psychosis in a first degree relative

15. Has poor peripheral arterial/venous access or recent wrist trauma that will restrict ability to gain venous access.

16. Is currently using prescription or non-prescription drugs and herbal supplements, which are deemed to affect the integrity of the study, within 7 days or 5 half-lives (whichever is longer) prior to the first dose of study medication. As an exception, paracetamol or acetaminophen may be used at doses of £1 g/day.

17. Has a history of sensitivity to any of the study medications or any of the excipient constituents.

18. Has a history of febrile illness within 5 days prior to the first dose

19. Has any condition possibly affecting drug absorption (eg, gastrectomy).

20. Has a positive urine drug screen on or after the screening visit during their active involvement in the study for opiates, methadone, cocaine, amphetamines (including MDMA), barbiturates, benzodiazepines and cannabinoids.

21. Is male and is unwilling to follow the contraception guidance or has a female partner of child bearing potential who is unwilling to follow the contraception guidance throughout the study.

22. Serum alanine aminotransferase (ALT) or aspartate aminotransferase (AST) $\geq$ 2.5 x upper limit of normal (ULN)

23. Total bilirubin $\geq$ 1.25 x ULN

24. Baseline resting QTcF > 470ms on 12 lead ECG

25. Positive hepatitis C antibody, hepatitis B virus surface antigen or hepatitis B virus core antibody at screening

26. Known congenital long QT syndrome

27. Below the lower limit of normal Hb, total WBC and neutrophils on blood counts as per the reference ranges of the laboratory conducting the tests.

*Experimental design and procedure***.** Patients were required to attend the study site for 5 visits: the initial screening visit, where patients were asked their medical history and underwent a physical and neurological exam and a vital observations check to make sure it was safe to administer the drug, and two visits for each of two study periods. On day 1 of each study period patients underwent baseline cognitive assessments to provide a profile for the patient and the physical exam and vital observation

For Review Only

checks were repeated. The clinical study drug, *saracatinib,* an inhibitor of the Src/abl family of kinases, was supplied by AstraZeneca. *Saracatinib* was provided as 50mg pink film coated tablets in bottles containing 32 tablets to cover a potential 16 days of dosing together with matching placebo tablets.
The drug was administered by the study doctor, who was also tasked with discharging the patients after a 3 hour stay during which patients were regularly checked upon by the study team.
Participants took a daily oral dose of 100mg of Saracatinib at one study period and matched placebo at the other study period, in a randomised order not known to the study team. Unblinding was done after the EEG data were pre-processed and ERP data extracted. For each study arm, the drug was administered every morning for 14 days (+/- 2 days), as it is known that 10 days of dosing with 100 mg Saracatinib will achieve a steady state level that is known to be well tolerated in patients with Alzheimer's disease (Nygaard et al 2015). A minimum 14-day washout period between the final dose of period one and the first dose of period 2 was kept. Participants returned to the study site on day 14 to undergo the study assessments (EEG session, clinical and psychiatric questionnaires, physical and neurological examination). We also collected blood samples to measure pharmacokinetics for the drug. Samples were collected at day1 pre-dose, day14 pre- and post-dose. In some cases (see table) it was not possible to collect all the samples due to issues with patients blood pressure.

Samples were collected in 4 mL K2EDTA green top Becton-Dickinson Vacutainers and after processing stored in 1.8 mL cryogenic vials. Upon collection the tubes were gently inverted 8 to 10 times to mix the additive with the collected blood prior to centrifugation and placed immediately on ice. Vacutainers were centrifuged for 10 minutes at approximately 1100 to 1300 x g (RCF) at 4°C in a pre-refrigerated centrifuge. Immediately following centrifugation, the plasma was removed from the packed cells and transferred into one (2 if the quantity allowed) pre-labelled 1.8 mL cryogenic vials. A minimum of 0.6 mL needed to be obtained for each aliquot. Plasma samples were frozen immediately at -20°C until shipment to the processing lab (Charles Rivers). No more than 45 minutes elapsed between blood collection and freezing the plasma sample.

For Review Only

Supplementary Information 7. a) *Parietal channels vMMN at early latencies (100-125 ms).* As we had hypotheses about parieto-occipital channels and frontal channels, but no strong hypotheses about parietal channels, we analysed vMMN at both 120-180ms and 100-125ms for these electrodes, finding that at later latencies there was a significant difference in the vMMN at P2 and a trend towards significance at PZ, reported in the main text. These results are congruent with the EEGlab study of standard vs. deviant waveforms. When analysed at the earlier latency where we found a vMMN at parieto-occipital channels, we did not find significant results at these channels. b) As some of the frontal electrodes showed a significance at the later latencies (~140-180ms) we also compared these differences in a shorter interval (140-180ms): F2 [$F(1,36) = 4.38$, $p = .04$] with PD-noVH MMN = 6.8 (sd 7.2) and PD-VH MMN= -0.83 (sd = 13.88), F4 [$F(1,36) = 5.02$, $p = .03$] with PD-noVH MMN = 6.9 (sd 7.6)

and PD-VH MMN= -2.11 (sd = 15.24), F6 [F (1,36) = 4.54, p = .04] with PD-noVH MMN = 5.62 (sd 8.7) and PD-VH MMN= -3.61 (sd = 16.93), and AF4, [F(1,26)=5.02, p =.03] with PD-noVH MMN = 6.8 (sd 7.2) and PD-VH MMN= -0.83 (sd = 13.88).

Supplementary Information and figure 8.

a) Waveform plots for the within group analysis. Frontal and parieto-occipital electrodes and the intervals where deviant-standard difference is significant (FDR correction is reported). Standard is green and deviant is pink.

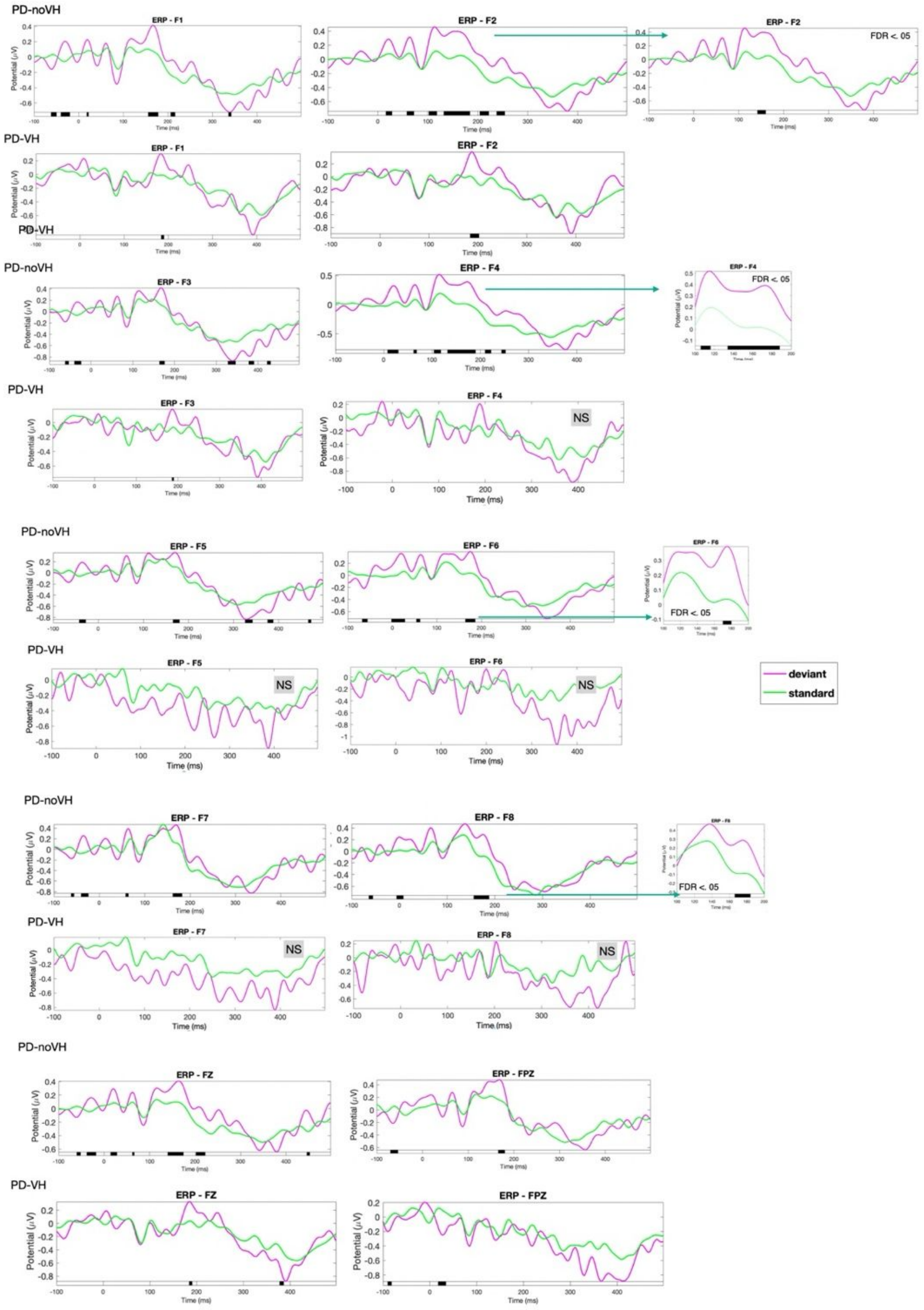

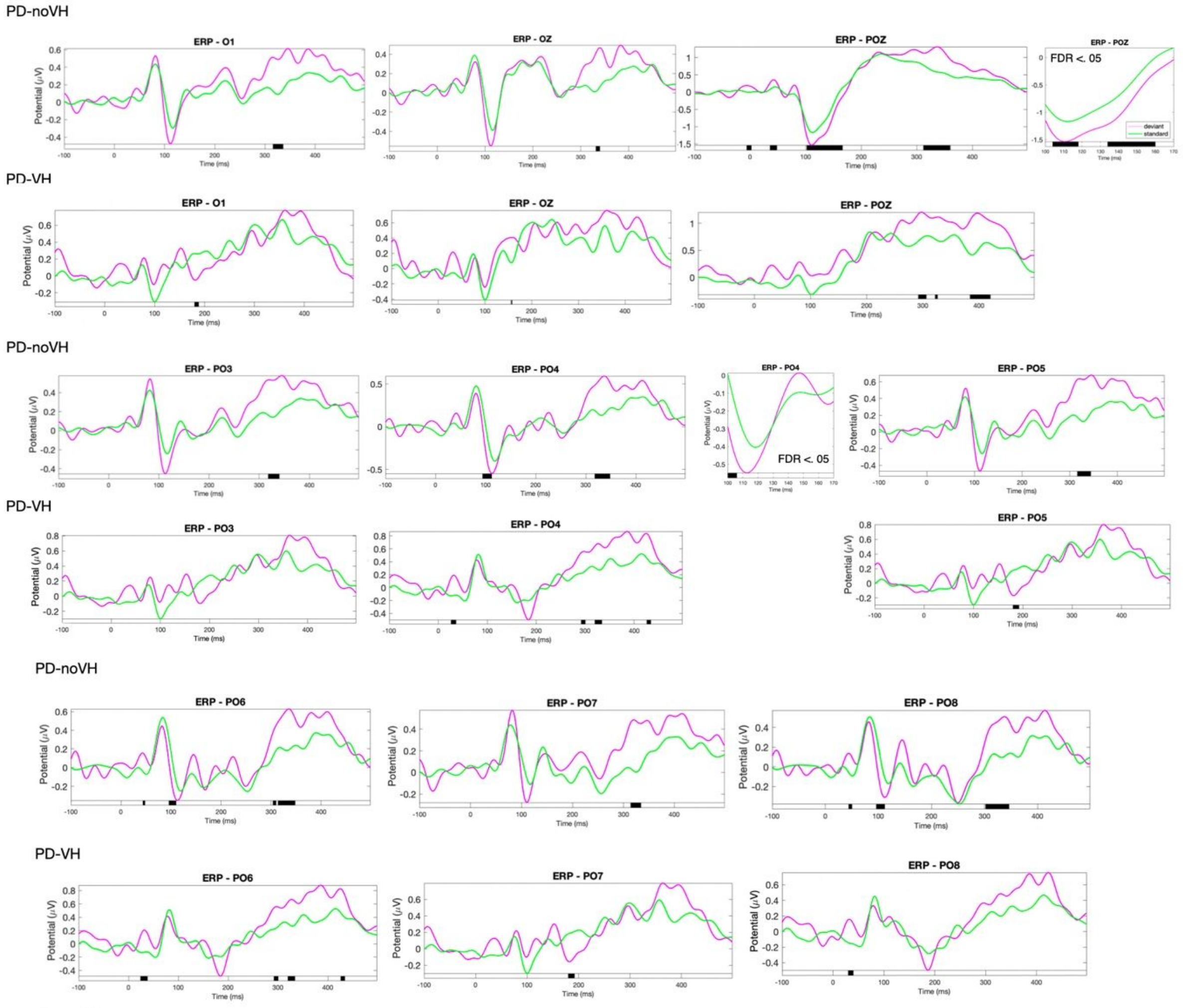

b) Between group analysis of ERP components. To further look at the differences between the groups and between standard and deviant, we also compared the groups with a 2x2 design in a EEGlab study structure.

Parieto-occipital channels. At POZ a significant difference is found for the condition in the latency of interest, surviving multiple comparison correction (*p*FDR <.05, see **Figure 8** for further details). Similar, but weaker, results were observed for O1 and PO3, not surviving multiple comparison correction. A similar pattern is observed at parietal channels (PZ, P1 with FDR, P2), with a difference for condition throughout the presentation interval, an early interaction (stimulus onset) and a late (~350ms) group difference; a similar profile is found at P4, with an earlier group difference detected. A difference for condition is also found at P3 (~150-200ms).

Frontal channels. At FZ, comparing VH and noVH participants on the standard/deviant ERPs we find that there is a difference of condition at ~90, and at intervals between 120 and 170ms with an earlier (< 100ms) interaction, nevertheless both do not survive multiple comparison correction. A similar pattern

is also observed for F2, F4, F5 and F6 (see **Figure 8**), with interactions at F2 and F6, consistently with what we found in the analysis performed in R and reported in the previous section. FPZ and FP2 show a significant difference for condition at 140-160ms (*p*FDR <.05).

For Review Only

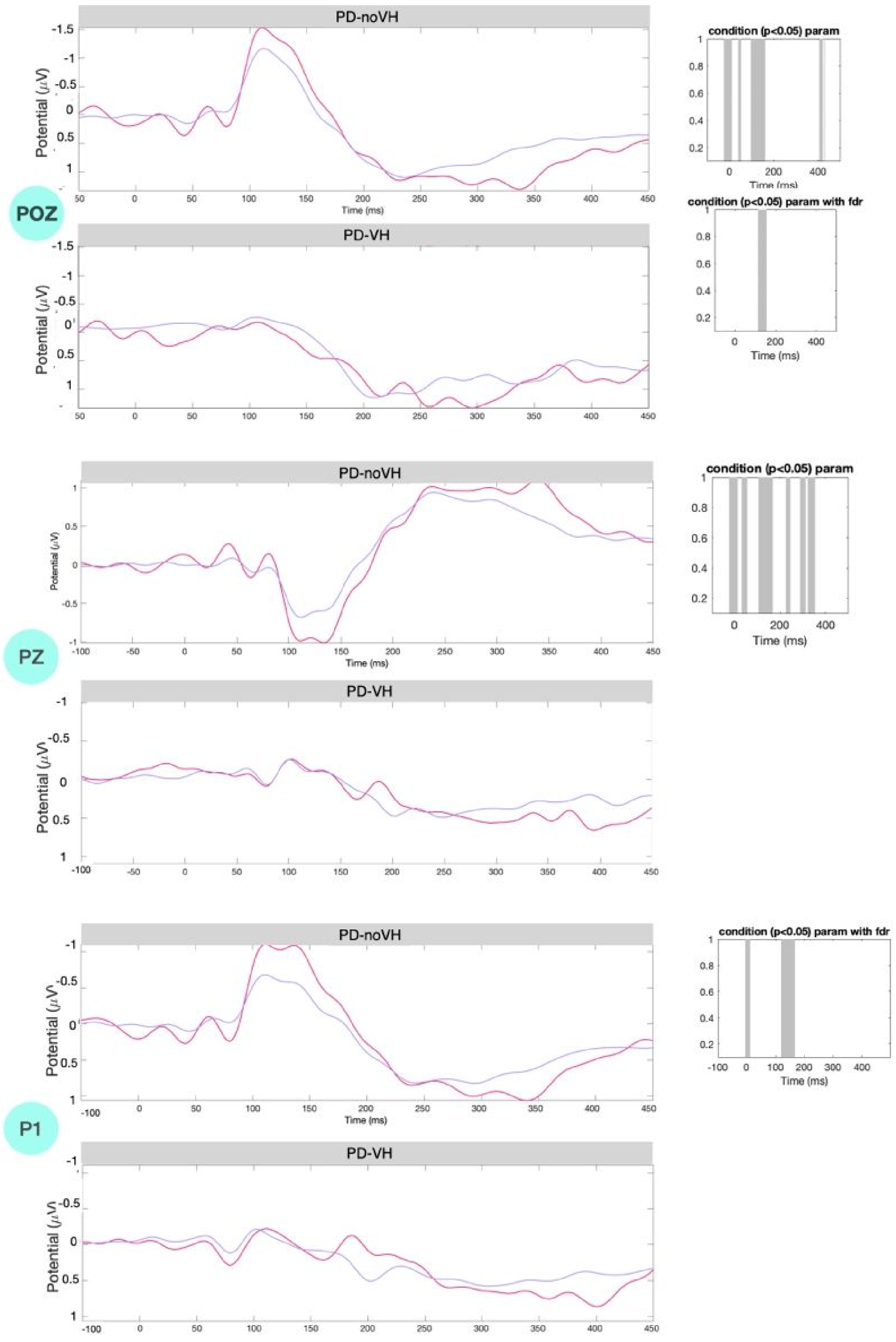
POZ
PD-noVH
PD-VH
Potential (μV)
Time (ms)
condition (p<0.05) param
condition (p<0.05) param with fdr
PZ
PD-noVH
PD-VH
Potential (μV)
Time (ms)
condition (p<0.05) param
P1
PD-noVH
PD-VH
Potential (μV)
Time (ms)
condition (p<0.05) param with fdr

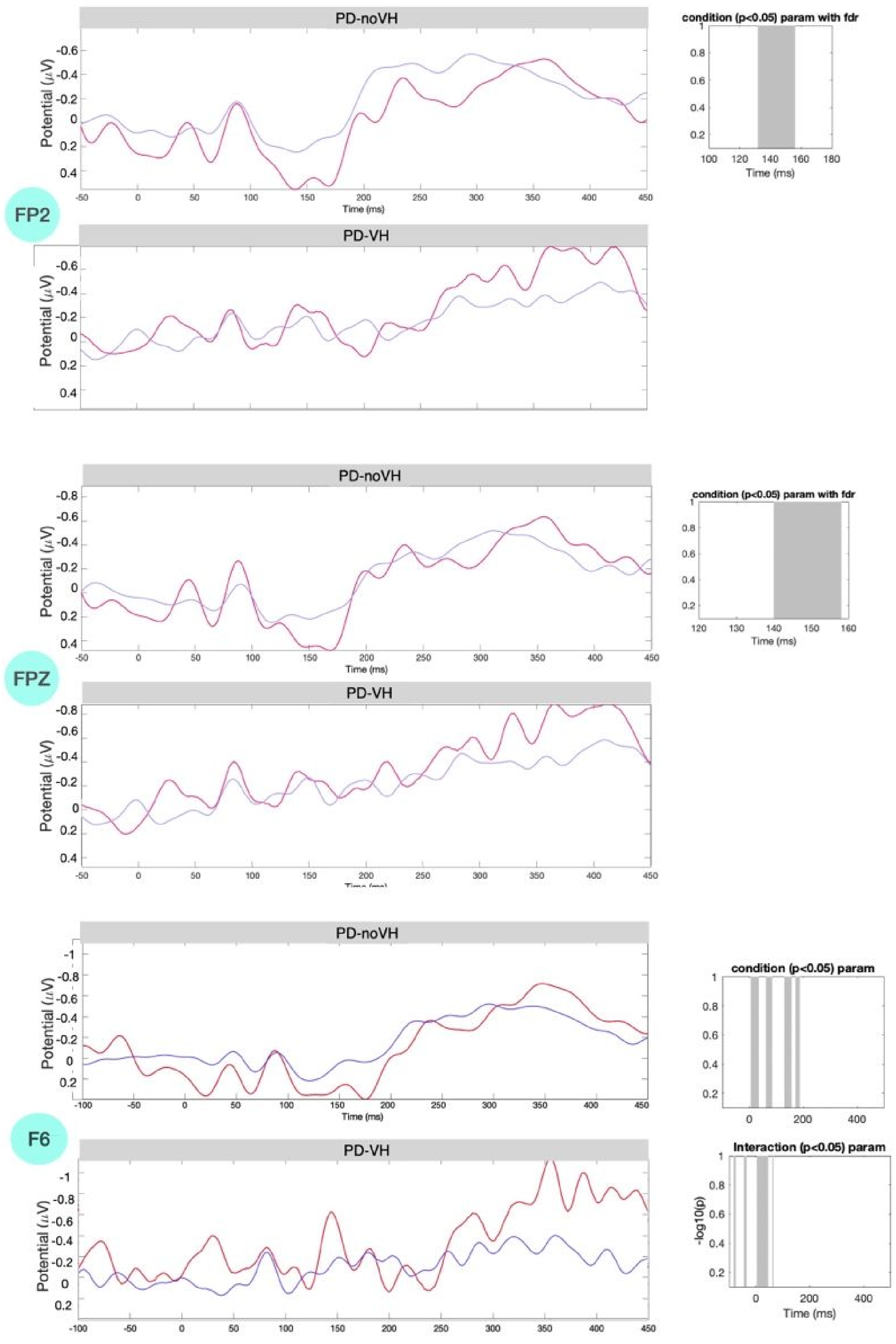
FP2
PD-noVH
PD-VH
condition (p<0.05) param with fdr
FPZ
PD-noVH
PD-VH
condition (p<0.05) param with fdr
F6
PD-noVH
PD-VH
condition (p<0.05) param
Interaction (p<0.05) param
Potential (μV)
Time (ms)
-log10(p)

Supplementary Information 9. *Correlational analysis with the clinical variables and neuropsychiatric assessments (PD-VH only). NEVHI scores at baseline, placebo and drug,*

As reported in the main text, SAPS-PD and NEVHI were positively correlated (*r*=.59 *p* =.006). A significant correlation was found between levodopa equivalent daily dose (LEDD) and disease onset (*r*=.58 *p* =.01) and disease onset and SCOPA-motor score (*r*=.58 *p* =.007). No other significant correlations were found. The hallucinations score at SAPS-PD and NEVHI temporal severity for complex VH scores were positively correlated both when using the NEVHI continuous severity score (*r* = .57, *p* =.009) and the temporal score we created multiplying ordinal duration and ordinal frequency (*r* = .47, *p* =.035).

Supplementary Information 10. *PD-VH drug additional information.*

id	age	onset	LED	MoCA	SAPS PD	NPI	CSI Motor	CSI Disability	CSI. Mot compl	CSI. Cognitive	SCOPA-motor
Pt2	76	7	370	25	11	3	4	4	1	1	31
Pt3	70	10	1080	25	7	6	4	3	2	1	28
Pt4	55	4	500	22	23	4	1	3	1	2	19
Pt5	75	10	635	24	18	3	3	4	4	4	36
Pt7	61	5	575	30	10	6	1	1	0	0	11

10a. *Baseline clinical information on the participants of the drug study.*

Period	Pt2	Pt3	Pt4	Pt5	Pt7
Period 1 day1 pre-dose	<LLOQ<0.500	<LLOQ<0.500	<LLOQ<0.500	<LLOQ<0.500	<LLOQ<0.500
Period 1 day14 pre-dose	<LLOQ<0.500	54.8	<LLOQ<0.500		<LLOQ<0.500
Period 1 day14 postdose	<LLOQ<0.500	53.9	<LLOQ<0.500	<LLOQ<0.500	<LLOQ<0.500
Period 2 day1 pre-dose	<LLOQ<0.500	<LLOQ<0.500	<LLOQ<0.500		<LLOQ<0.500
Period 2 day14 pre-dose	114	0.577		61.8	85.8
Period 2 day14 postdose	111	<LLOQ<0.500	70.2	103	73.1

10b. *PK sample analysis.* grey cells = no drug period or day1 pre-dose, white cells = drug period. PT3 had the drug on period 1 and placebo on period 2, whereas the others were randomised to the opposite order.

10c. Wilcoxon paired tests revealed no difference between drug and placebo conditions also for pareidolia [NEVHI_{PLA}= 3.8 ± 4.15, NEVHI_{DRUG}= 5.4 ± 4.26, *p* =.2], presence [NEVHI_{PLA}=

1.2 ± 3.3, $NEVHI_{DRUG}$= 3 ± 3.3, p =.5] and passage [$NEVHI_{PLA}$= 1 ± 1.1, $NEVHI_{DRUG}$= 1.6 ± 1.6, p =1] VH.

The table shows the individual scores (P is for 'placebo').

	SAPS_P	SAPS_DRUG	NPI_P	NPI_DRUG	NEVHI_P	NEVHI_DRUG
Pt2	7.00	5.00	3.00	4.00	9.00	1.00
Pt3	9.00	3.00	1.00	2.00	7.00	8.00
Pt4	15.00	8.00	4.00	4.00	9.00	1.00
Pt5	10.00	8.00	13.00	11.00	6.00	6.00
Pt7	8.00	5.00	4.00	4.00	5.00	9.00

10d. *vMMN analyses at parieto-occipital channels.* At POZ, we find no significant difference between drug and placebo condition [Z = -.94, p= 0.35, $vMMN_{PLA}$= -0.46 ± 0.53, $vMMN_{DRUG}$= -0.22 ±0.74]. When carrying out the fixed effects analysis with POZ, PZ and OZ, we find similar results [Z = -.126, p= 0.9, $vMMN_{PLA}$= -0.29 ± 0.44, $vMMN_{DRUG}$= -0.33 ±0.63].

For Review Only

10e. *Individual waveforms in the placebo and drug conditions: Frontal, Parietal and Parieto-occipital.*

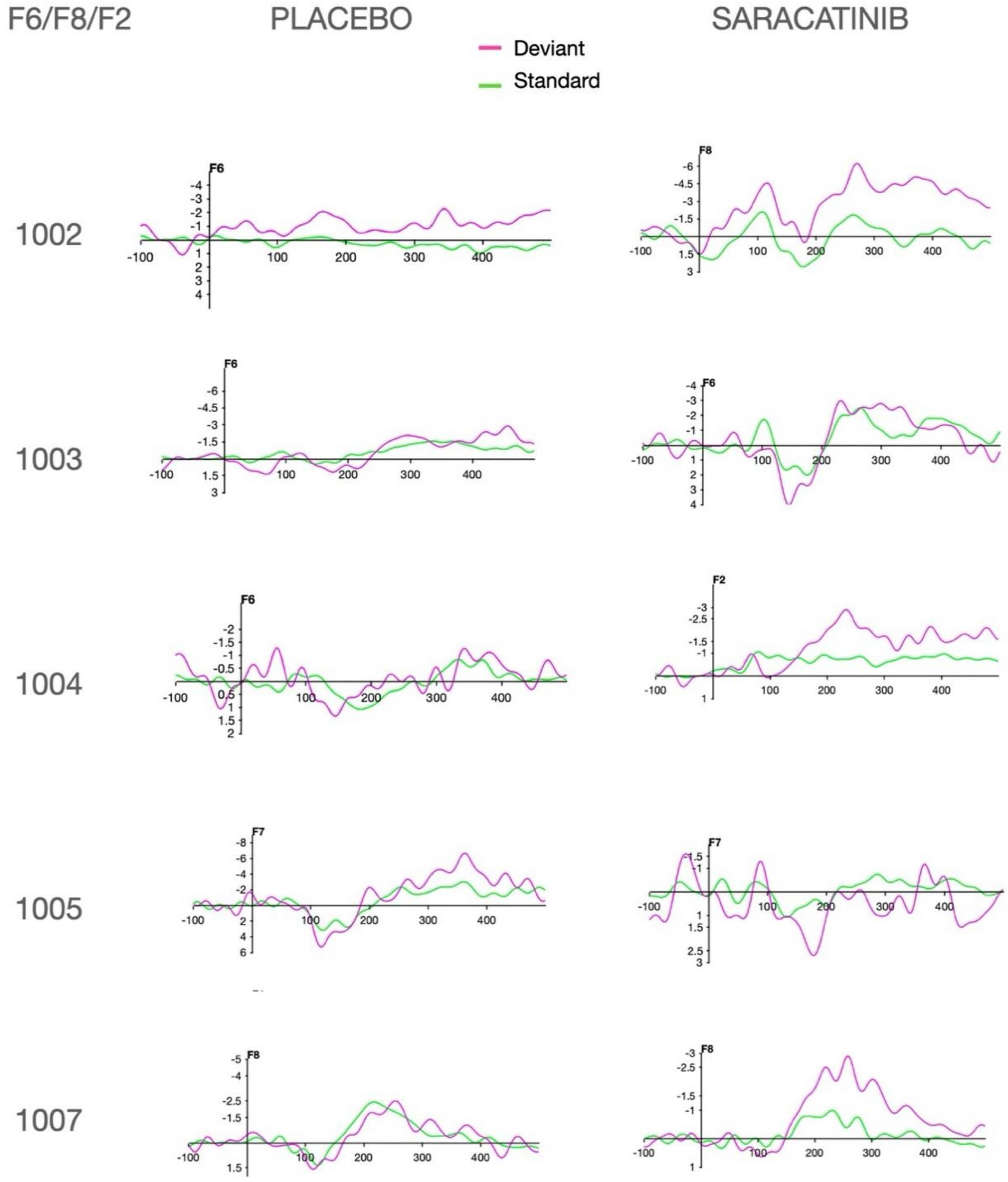

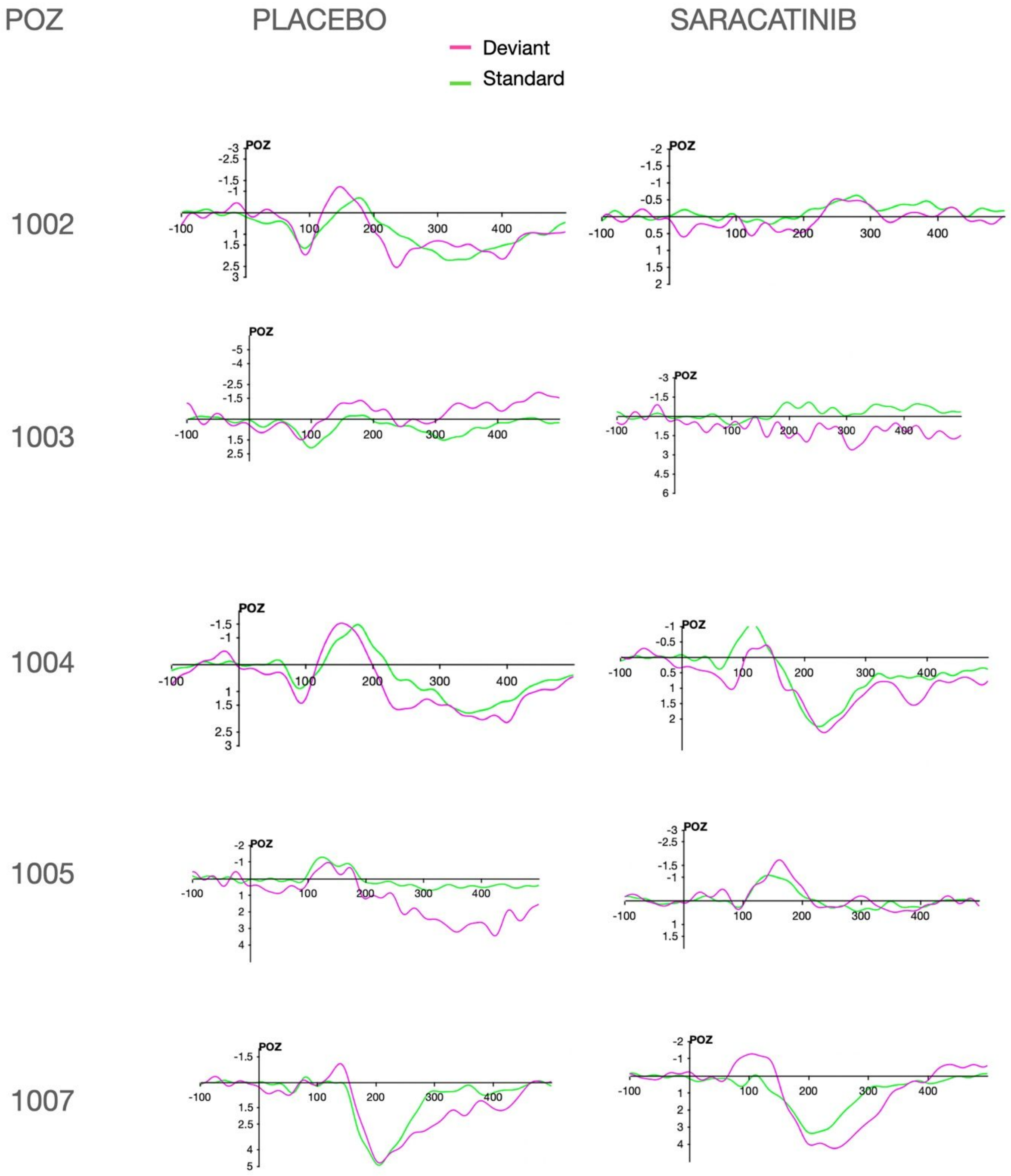
POZ
PLACEBO
SARACATINIB
Deviant
Standard
1002
1003
1004
1005
1007

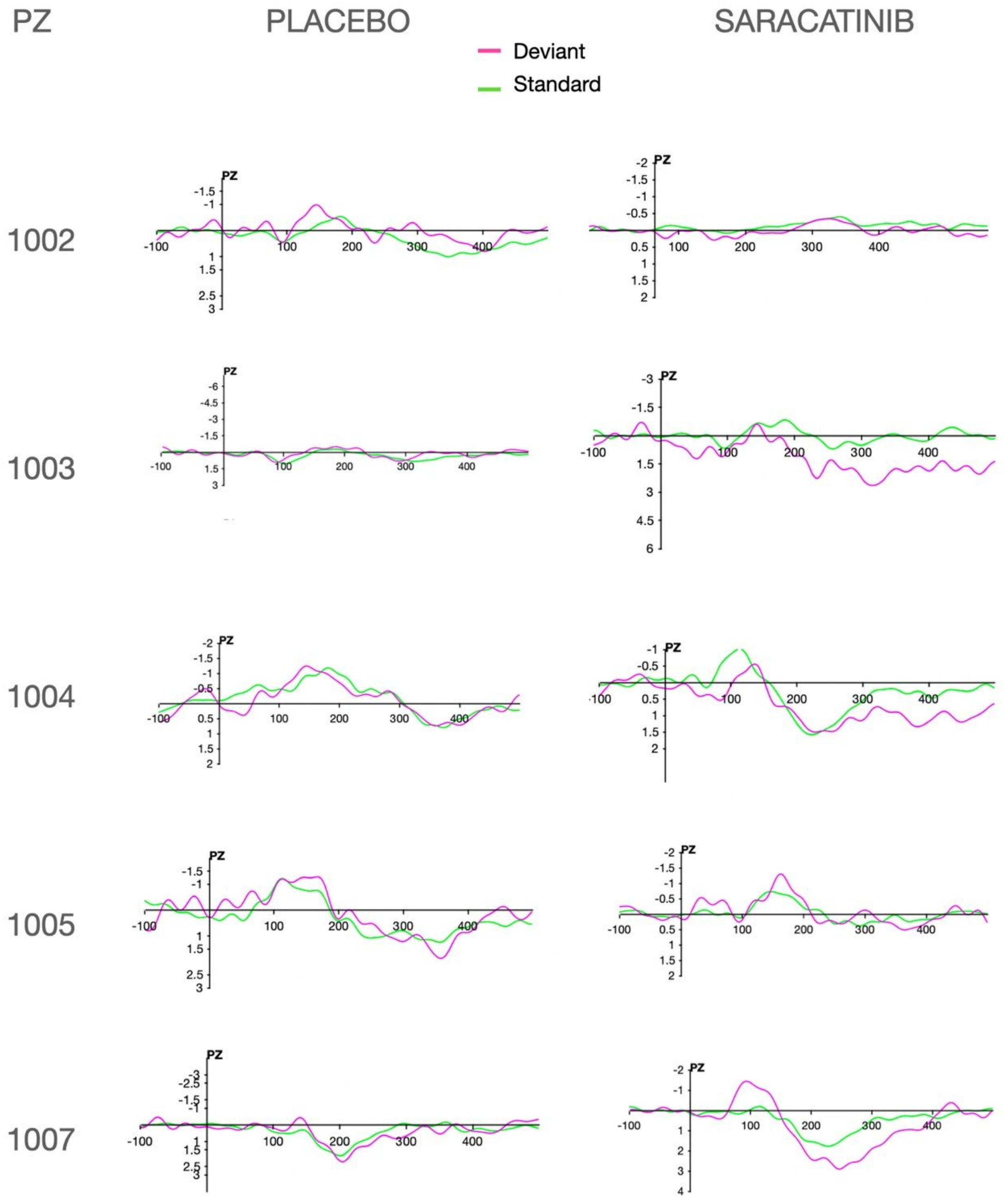
PZ
PLACEBO
SARACATINIB
Deviant
Standard
1002
1003
1004
1005
1007

Supplementary References

Martinez-Martin P, Forjaz MJ, Cubo E, Frades B, de Pedro Cuesta J, et al. (2006) Global versus factor-related impression of severity in Parkinson's disease: A new clinimetric index (CISI-PD). *Mov Disord* 21: 208–214.M

Chaudhuri, K. R., Martinez-Martin, P., Brown, R. G., Sethi, K., Stocchi, F., Odin, P., ... & Schapira, A. H. (2007). The metric properties of a novel non-motor symptoms scale for Parkinson's disease: results from an international pilot study. *Movement disorders*, *22*(13), 1901-1911.

Voss, T., Bahr, D., Cummings, J., Mills, R., Ravina, B. & Williams, H. 2013. Performance of a shortened Scale for Assessment of Positive Symptoms for Parkinson's disease psychosis. *Parkinsonism Relat Disord,* 19**,** 295-9.

D'Antonio, F., Boccia, M., Di Vita, A., Suppa, A., Fabbrini, A., Canevelli, M., ... & Ffytche, D. (2022). Visual hallucinations in Lewy body disease: pathophysiological insights from phenomenology. *Journal of Neurology*, *269*(7), 3636-3652.

Whitney, K. A., Shepard, P. H., Mariner, J., Mossbarger, B., & Herman, S. M. (2010). Validity of the Wechsler Test of Adult Reading (WTAR): Effort considered in a clinical sample of US military veterans. *Applied Neuropsychology*, *17*(3), 196-204.

Cummings JL, Mega M, Gray K, Rosenberg-Thompson S, Carusi DA, Gornbein J. The Neuropsychiatric Inventory: comprehensive assessment of psychopathology in dementia. *Neurology.* 1994;44(12):2308–2314. doi: 10.1212/WNL.44.12.2308

Fernandez, H. H., Aarsland, D., Fénelon, G., Friedman, J. H., Marsh, L., Tröster, A. I., ... & Goetz, C. G. (2008). Scales to assess psychosis in Parkinson's disease: critique and recommendations. *Movement disorders: official journal of the Movement Disorder Society*, *23*(4), 484-500.

Anwyl-Irvine, A. L., Massonnié, J., Flitton, A., Kirkham, N., & Evershed, J. K. (2020). Gorilla in our midst: An online behavioral experiment builder. *Behavior research methods*, *52*, 388-407.

Nygaard, H. B., Wagner, A. F., Bowen, G. S., Good, S. P., MacAvoy, M. G., Strittmatter, K. A., ... & van Dyck, C. H. (2015). A phase Ib multiple ascending dose study of the safety, tolerability, and central nervous system availability of AZD0530 (saracatinib) in Alzheimer's disease. *Alzheimer's research & therapy*, *7*(1), 1-11.

For Peer Review Only
