# Supplementary material for: Visual mismatch negativity in Parkinson's psychosis and potential for testing treatment mechanisms
Source: Brain Commun. 2024 Sep 3;6(5):fcae291. doi: 10.1093/braincomms/fcae291 (PMC11443450; doi:10.1093/braincomms/fcae291)
Supplement: fcae291_Supplementary_Data [file fcae291_supplementary_data.zip › Supplementary Materials.pdf]

Supplementary Materials

Supplementary Information 1. *Study questionnaires details* *2*

Supplementary Information 2. *Behavioural task pilot* *5*

Supplementary Information 3. *Behavioural task PD-noVH and PD-VH patients* *9*

Supplementary Information 4. *Study 1 details and EEG procedure* *10*

Supplementary Information 5. *EEG pilots* *13*

Supplementary Information 6. *Behavioural study in healthy volunteers* *14*

Supplementary Information 7. *Drug study design and procedure* *15*

Supplementary Information 8. *Parietal channels early vMMN* *18*

Supplementary Information 9. *Individual VEPs.* *19*

Supplementary Information 10. *Between group analysis of ERP components* *21*

Supplementary Information 11. *Correlational analysis with the clinical variables* *23*

Supplementary Information 12. *PD-VH drug additional information.* *23*

Study Flowchart 28

Supplementary References *29*

Supplementary information 1. *Details about the assessments and questionnaires used to investigate patients motor symptoms and their hallucinations and other non-motor symptoms.*

Participants underwent a phone pre-screening and attended one study day at the clinical research facility (CRF) of King's College Hospital (London, UK) where they completed screening, for which we collected medical history, medication, physical and non-motor examinations[1], PD-specific exams to assess disease severity (Clinical Impression of Severity Index (CISI-PD)[2], motor examination (SCOPA-MOTOR) and conducted the EEG session. We also conducted a baseline cognitive assessment comprising the Montreal Cognitive Assessment (MoCA), verbal and semantic fluency, months reversed and the Wechsler Test of Adult Reading (WTAR) for estimation of pre-morbid ability[3]. Inclusion criteria included MoCA above 21 and NPI>4 or recurrent visual hallucinations as recorded with other questionnaires (see below and SI1) if there was no study partner. Exclusion criteria included ongoing disabilities, medical history and medical conditions that could interfere with the study conduct or clinical assessments; this included current psychiatric diagnosis (except psychosis in the PD-VH group) as this could interfere with the EEG task results and severe motor impairment judged to make the study days too challenging for participants. People on medication potentially interfering with study measurements were excluded on a case-by-case basis.

The Non motor symptoms questionnaire (NMSQ) is a nine-domain questionnaire consisting of 30 questions designed to test the frequency and severity of the non-motor symptoms of Parkinson's disease over the past month[1].

Supplementary Table 1a. *NMSQ domains details.*

Domain	Group	Mean	Std. Dev	*F*	*p*
**Gastrointestinal**	PD-noVH	4.78	0.94	21.74	<.001
	PD-VH	2.68	1.67		
**Perceptual**	PD-noVH	0.22	0.55	55.72	<.001
	PD-VH	1.63	0.60		
**Attention**	PD-noVH	0.50	0.79	10.64	0.002
	PD-VH	1.37	0.83		
**Sleep**	PD-noVH	1.28	1.02	13.43	0.001
	PD-VH	2.74	1.37		

To investigate visual hallucinations, study partners completed the hallucinations and delusions scale of the Neuropsychiatric Inventory[4]. The Neuropsychiatric inventory (NPI) was administered to the study partner as standard procedure and assesses neuropsychiatric symptoms. It consists of 12 items, with section A (delusions) and B (hallucinations) exploring the domains of these symptoms. If symptoms are present, questions about frequency (1-3) and severity (1-4) are collected and multiplied, and hallucinations and delusions are added up to get the NPI total score[4].

PD-VH participants were administered the Scale for the Assessment of Positive Symptoms-PD (SAPS-PD) adapted for PD from schizophrenia[5] and an adaptation of the NEVHI to assess the phenomenology of visual hallucinations[6,7].

The Scale for the Assessment of Positive Symptoms (SAPS) originally designed for schizophrenia has been found to be reliable for people with Parkinson's disease[8] . The SAPS-PD is composed of the 9 items found to be most frequently reported in patients with PD psychosis[5]. In the article, we only used scores for hallucinations in these patients. Only one patient presented delusions, and they retained insight about it. SAPS-PD scores reported in the article range from 2 to 20. The hallucinations scale of the SAPS-PD counts 5 items: auditory hallucinations, voices conversing, visual hallucinations, somatic/tactile hallucinations, global rating of hallucinations. The maximum score for each item is five, for a total score of the hallucinations scale reaching a maximum of 25. Participants with >10 had also auditory or somatic hallucinations.

The NEVHI is a visual complaints questionnaire that is used to assess the phenomenology of visual hallucinations[6]. The scale includes 6 questions assessing different types of VH: complex hallucinations, pareidolia (a specific type of visual illusion), presence, passage, simple hallucinations. The last question 'other' is designed to pick up any other visual experience and classified under the relevant section. The NEVHI also provides a score for distress, and information about insight and delusions specific to the type of VH, differing from the general delusions assessed by SAPS-PD. Participants' scores at the different VH types are also reported in **Supplementary Table 1b**.

The adapted version of the NEVHI has no scoring rules, so we computed temporal severity by multiplying duration and the frequency of the VH, using an ordinal scale following as closely as possible as that used in a previous study[7]. We also computed a continuous temporal severity score multiplying the raw number of minutes spent hallucinating by the raw number of VH in a month. We computed temporal severity as a continuous measure and as an ordinal measure, as two patients constituted outliers as they hallucinated 'continuously' or 'for hours', whereas the rest of the patients had hallucinations lasting seconds or minutes. Thus, to be able to carry out correlational analyses retaining all the patients but taking this into account, we used an ordinal scale as in D'Antoni et al.[7] for duration, where seconds =1, minutes =2, hours =3 and continuously =4. For frequency, as our new version of the NEVHI did not have an ordinal scale to rate this, but asked participants to state how many VH in a month they had, we adapted the ordinal scale reported in the article cited above. **Supplementary Table 1c** summarises how we computed the temporal severity score, using the raw data from the complex VH question as an example.

	Complex	Pareidolia	Presence	Passage	Simple
N	17	14	15	8	3
%	85	70	75	40	15
Duration (sec)	1119*	1296*	1243*	8	4841.66*
Frequency(vh/month)	29.5	23.50	20.39	25.58	6.17
Distress	0.5 (2)	2.23 (4)	0.73 (4)	0	0.7(1)
Frustration	1.25 (6)	1.93 (4)	1.53 (5)	0	0
Insight***	0.07% (1)	0.14% (2)	1.46% (5)	0	33% (1)
Delusion***	0.1% (2)	0.07% (1)	0.13v (2)	12.5%(1)	0

Supplementary Table 1b. *-NEVHI scores reported per scale. Duration is reported in seconds, frequency in how many VH are experienced in a typical month. Average scores are reported for distress and frustration ratings (rated on a scale from 0 to 10), in brackets we report how many participants had the symptom.*

% of participants for insights and delusions are reported: we included in the calculations those who had a certainty the VH was real at the time they occurred; 2 more participants stated it might happen at times.

*one participant said they had one of the VH type continuously; in order to put a number in seconds we assigned 18000 seconds to their VH

***Insight: some patients said that for the reported VH type it took them a few seconds to realise the experience is indeed a VH; delusion: 2 of the patients report VH-specific delusions in the sense that they responded ‘sometimes’ to the questions asking if they had a different explanation for their VH.

Supplementary Table 1c *– The table shows how from raw scores of duration (seconds) and frequency (how many experiences in a month) have been converted to ordinal scores and in the multiplicative factor that we used as a measure of temporal severity.*

**Duration (sec**	**Ordinal_duration**	**N. of VH per month**	**Ordinal category assigned**	**Ordinal_frequency**	**Severity (duration*freq)**
30	1	3	every few weeks	1	1
2.5	1	12	more than once a week	3	3
5	1	15	more than once a week	3	3
0	0	0	Not reported	0	0
0	0	0	Not reported	0	0
1	1	40	more than once a day	5	5
900	2	4	every few weeks	1	2
10	1	2.5	every few weeks	1	1
2	1	50	more than once a day	5	5
5	1	60	more than once a day	5	5
0	0	0	Not reported	0	0
5	1	2	every few weeks	1	1
5	1	21	daily/almost daily	4	4
5	1	8	more than once a week	3	3
5	1	55	more than once a day	5	5
5	1	90	every few hours	6	6
2	1	12	more than once a week	3	3
continuously (18000)	4	2 to 6	once a week	2	8
60	2	25	daily/almost daily	4	8
5.5	1	110	every few hours	6	6

Supplementary Information 2. *Behavioural task pilot with healthy participants.*

The EEG task administered to PD patients was programmed in the software compatible with the EEG Neuroscan computer at the research facility *(Presentation v.17.2)* with stimuli presented in a pseudo-randomized manner, with details reported in the Methods in the main text. In the online behavioural pilot, we used the same stimuli and timings used in the EEG task, but the bars showed on the screen were blue with a white coloured background. whereas in the visual task shown during the EEG session the bars were black with a light grey background and a dim light, for participants' comfort as the light grey background was easier on the eyes.

The peripheral stimuli aimed at eliciting the vMMN were symmetrical flashing bars, with the variation being in orientation: bars were presented with a 0 (rare deviant), 30 (frequent deviant) and 60 (standard) degree orientation (see **SI2** for details). The bars for each orientation were simultaneously presented in the left and right peripheral visual field (offset 400/-400) centred on the horizontal meridian, staying on the screen for 50ms. The cross changes had a duration of 200ms. The stimulus onset asynchrony (SOA) was 600ms.

Healthy participants Pilot. Ninety-four healthy volunteers participated in the pilot study (64 F, 30 M, age range 25-73 years, mean age 41.1 years, SD 11.4), with 66 (28 drop out) completed the online visual task. Participants had normal or corrected to normal vision. The volunteers signed written informed consent before participating in the study. The online task was designed with the online experiment platform Gorilla[9].

Participants were instructed to pay attention at fixation cross at the centre of the screen and to ignore any peripheral stimuli. They were instructed to press the left or the right button of their keyboard when the cross became bigger or smaller.

The peripheral stimuli were flashing bars that should elicit the MMN in the EEG task. The standard orientation of the bars had a target prevalence of 64%, the frequent deviant of 24% and rare deviant of 12%. In the pilot study participants were divided into three groups of 22, each group with a different visual configuration of the standard orientation (30°- 60°- 90°) to determine whether this influenced the salience of the peripheral stimuli or waveform of the visual evoked potential. At the end of the task, participants were also asked to report the most and least frequent orientation of the peripheral flashing bars presented in the task to investigate the extent to which they might have redirected their attention to the peripheral stimuli. For the EEG study, only one of the configurations of the visual task was shown (see figure S1 below). To select which condition to use a small pilot EEG study was carried out with healthy participants. Upon repeated testing with the three task configurations, we chose configuration 2, as it evoked the clearest visual evoked response but did not differ from the other configurations in terms of evoked waveforms.

The bar distance from the fixation cross was at a visual angle of approximately ± 9 °; the same angle was measured for the bar length; the bar width was at a ± 2 ° visual angle; for the screen width the visual angle was 27 ° and for screen height the angle was 21 °.

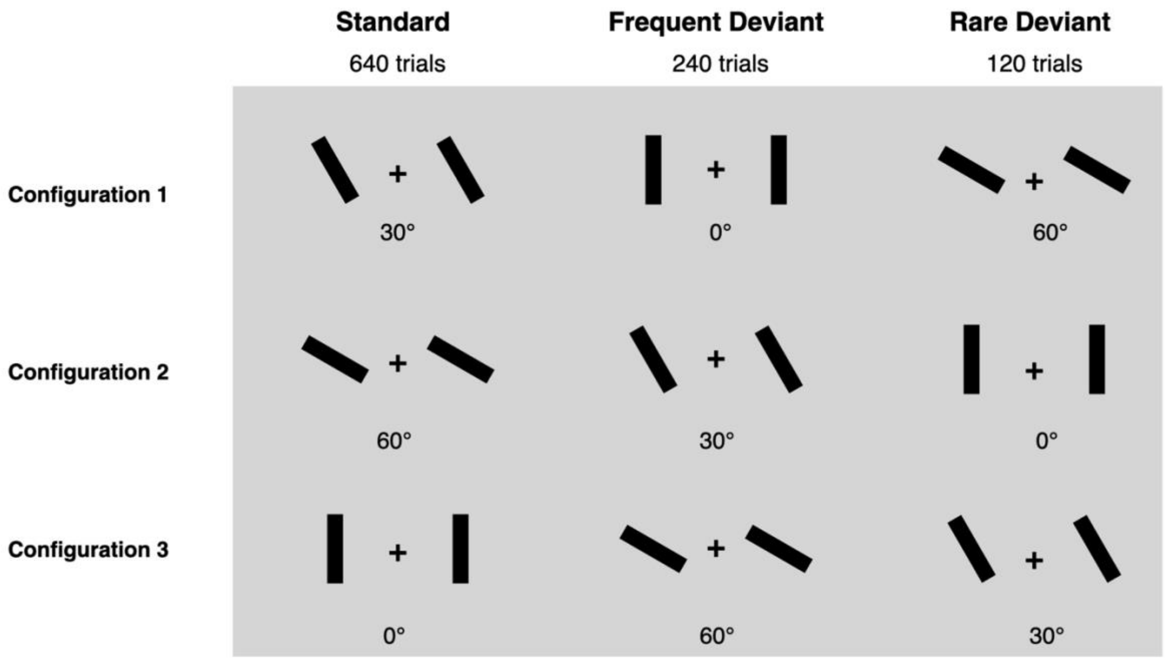

Supplementary Figure 1*: The visual configurations of the flashing bars used in the pilot study with the target prevalence of the three orientations of the bars in the trials. Configuration 2 was selected for the study.*

During the visual task, similar stimuli and instructions were provided to the participants of pilot and EEG studies. We included in the analysis of the orientation frequency responses, only participants that achieved 70% accuracy rate at the cross-change detection task (58, 8 excluded).The participants of both studies were able to discriminate the cross change displayed at the centre of the screen and their performance was associated with different types of errors: i) omission errors (no response for trial change); ii) commission errors outside trials (responses for no trial change) iii) response errors (incorrect response to the trial).

- Under configuration 1 (30 standard) the mean accuracy (%) to cross change was 84% (SD 24.9), with omission errors on 10% of the trials (10.1, SD 23.0), commission errors on 17% of the trials (16.1, SD 26.1) and response errors on 6% of the trials (6.10, SD 8.72)
- Under configuration 2 (60 standard) the mean accuracy (%) to cross change was 87% (SD 14.6), with omission errors on 6% of the trials (5.48, SD 9.09), commission errors on 60% of the trials (59.6, SD 170) and response errors on 7% of the trials (7.05 SD 12.5)
- Under configuration 3 (90 standard) the mean accuracy (%) to cross change was 93% (SD 14.0) with omission errors on 5% of the trials (5.19 SD 12.9), commission errors on 16% of the trials (16.1 SD 24.6) and response errors on 2% of the trials (2.38 SD 3.38).

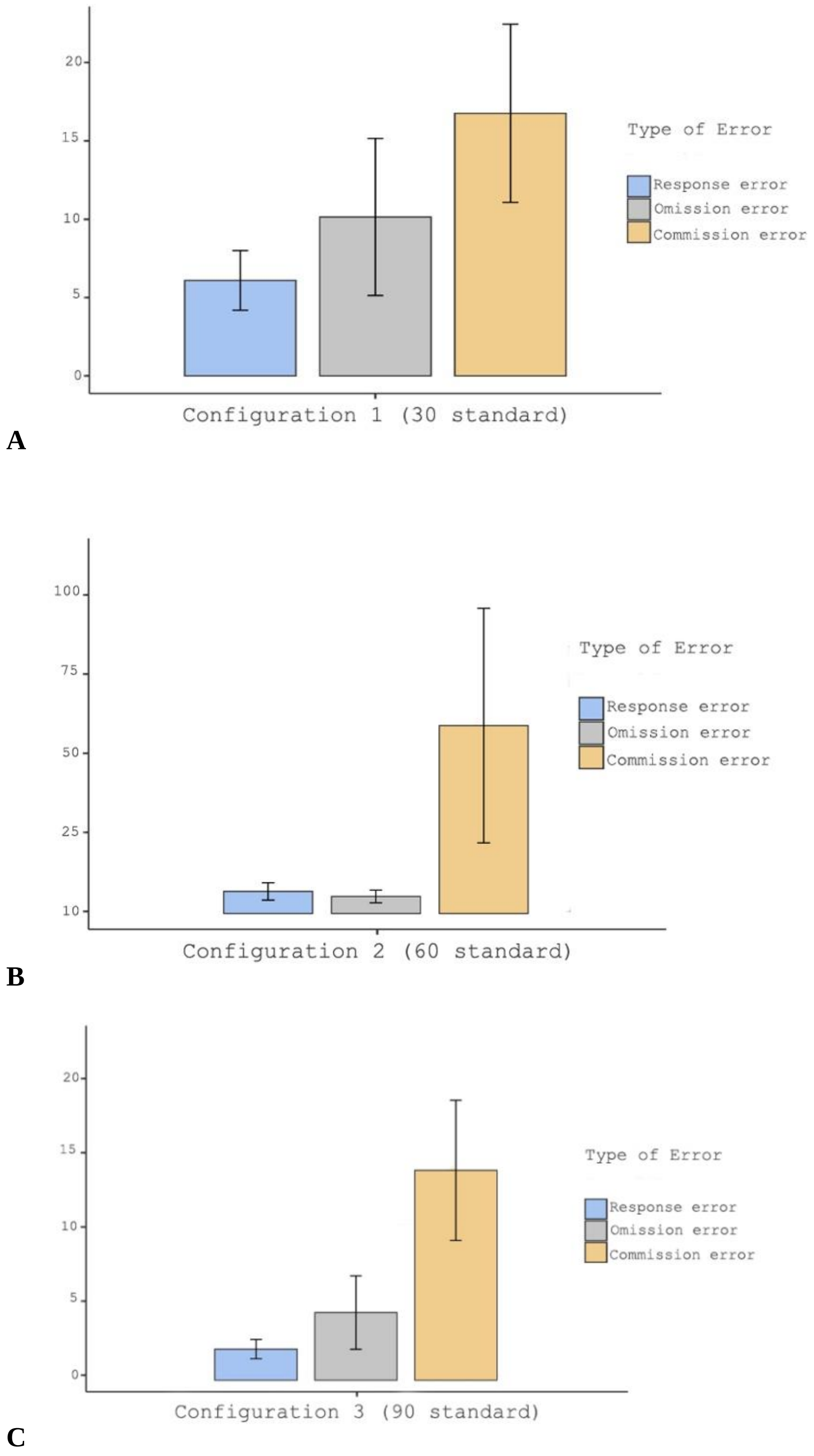

Supplementary Figure 2. *Bar plots displayed the type of errors made with: A) Configuration 1; B) Configuration 2, C) Configuration 3. Data was analysed with Chi Squared statistics showing a significant association between the three configurations of the visual task (30°,60°,90°) and the behavioural responses to the orientation frequency discrimination task (Most frequent orientation: χ (4, N = 58) = 27.054, p= .000; Least frequent: χ (4, N = 58) = 19.828, p= .001). N participants = 94.*

The results of Chi Squared test shows a significant association between the three configurations of the visual task (30°,60°,90°) and the behavioural responses to the orientation frequency discrimination task

(Most frequent orientation: χ (4, N = 58) = 27.054, p= .000; Least frequent: χ (4, N = 58) = 19.828, p= .001). The association between the variables was not significant when only configurations 1 (standard 30°) and 2 (standard 60°) were included in the analysis (Most frequent orientation: χ (2, N = 38) =1.292, p= 0.524; Least frequent orientation: χ (2, N = 38) =3.474, p= 0.176). Therefore, the observed responses were significantly different from the expected responses when the configuration 3 (90° standard) was included in the analysis but not when only configuration 1 (30° standard) and 2 (60° standard) were included.

Supplementary Information 3. *Behavioural task with patients: PD vs. PD-VH.*

When comparing the two groups of patients at the cross-change detection task, patients with PD-VH performed worse than PD-noVH [F(1,34) = 10.64, *p* = .003]. For PD-noVH, the overall accuracy was 91.3% (mean =40.17 sd = 4.63) whereas for PD-noVH it was 70% (mean = 31.06 sd 10.90). PD-VH and PD-noVH did not differ in the amount of trials they missed however [F(1,34) = 2.86, *p* = .100].

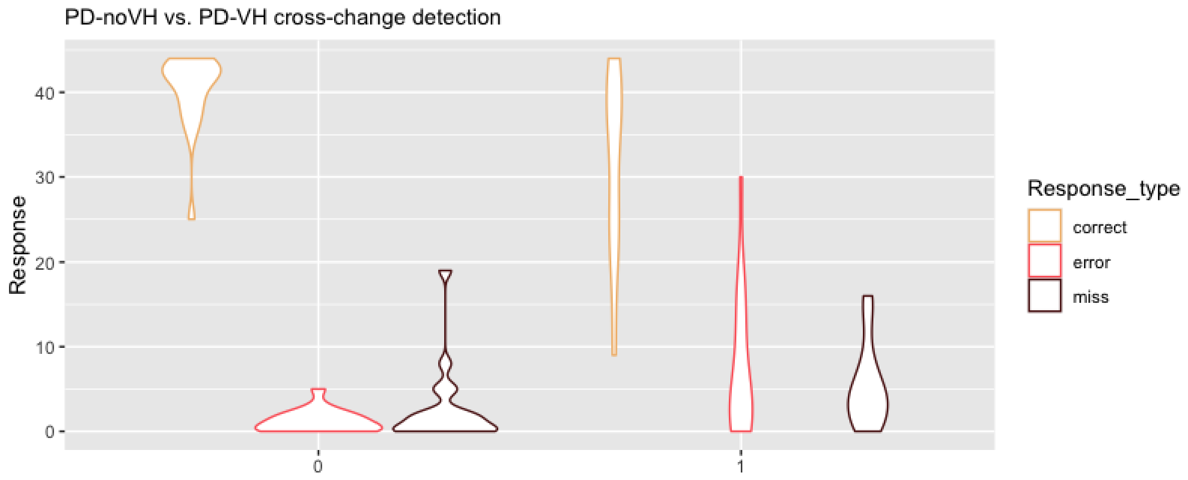

Supplementary Figure 3. *On the left panel PD-noVH (18), on the right PD-VH (20). Violin plots represent distribution of responses: in yellow the % of correct responses to the cross change (bigger or smaller); in pink the % of errors and in purple the misses. We performed a one-way ANOVA finding that PD-VH performed worse than PD-noVH [F(1,34) = 10.64, p = .003] but the two groups did not differ in the misses [F(1,34) = 2.86, p = .100].*

One PD-noVH and two PD-VH participants were excluded from the behavioural analysis. Inspection of their response outputs was necessary to detect that participants were paying attention to the task (responses given in correspondence to triggers); however, these participants did not understand completely the instructions, and pressed the same key for all trials. We excluded these participants as we thought this would affect the results not allowing to investigate relevant differences.

Supplementary Information 4. *Additional details on Study 1, description of the vMMN task, power calculations and EEGlab preprocessing.*

Participant medication: additional information. We provide a table with medication information for each participant, including whether they were on dopamine agonists, on SSRIs or any other relevant drug.

VH	Age	Onset	Sex	LEDD	Dopamine agonist Y/N	SSRI Y/N	Specific drug information
no	74.00	2.00	male	625.00	no	no	sinemet, rivaroxpane
no	73.00	6.00	male	310.00	yes	no	sinement, roprinole
no	52.00	3.00	female	765.00	no	no	stalevo
no	49.00	2.50	female	0.00	no	no	drug naïve, vitamines
no	77.00	3.00	male	250.00	no	no	sinemet,rasagline, entacapone,olanzapine
no	52.00	1.00	female	403.00	no	no	citalopmra, siniement,rsasgiline,lamotragine
no	73.00	10.00	male	150.00	no	no	sinemet
no	64.00	4.30	male	150.00	no	no	amiodpine
no	81.00	9.00	male	500.00	no	no	rasagiline, sinement, atrial fibrillation medication
no	56.00	3.50	female	920.00	yes	no	co-careldopa, amantadine, ropirinol, vitamins
no	77.00	3.50	female	100.00	no	no	rampiril, atorvastatin, rasagiline
no	72.00	4.30	male	500.00	no	no	co careldopa, rasagiline, atorva, lasnopraz,naproxen
no	65.00	8.00	male	0.00	no	no	cocodamol
no	49.00	2.00	male	560.00	yes	no	sinemet, ropirinole, ramipril
no	75.00	4.00	male	400.00	no	no	atorva, ramipril, co-careldopa, solifenacin
no	61.00	2.00	male	281.00	no	no	co-careldopa
no	66.31	4.19	female	250.00	no	no	sinemet, madopar
no	67.00	10.00	female	175.00	yes	no	sinemet, rasagiline, rotigotine, vitamis, propanolol, naproxen
yes	74.00	5.00	female	780.00	yes	no	rotigotine, sinement, clonazepam, aziled
yes	67.00	3.00	male	445.00	no	no	opicapone, madopar, sinemet, rasagiline
yes	53.00	3.00	male	408.00	yes	no	rasagiline, ropirinole, sinement, propanilol
yes	65.00	8.00	female	150.00	yes	yes	ropirinole, cobenacareldopa, simvastatine, diltazem, sertraline
yes	74.00	4.00	male	175.00	missing	no	no specific info
yes	56.00	4.00	male	325.00	missing	no	no specific info
yes	72.00	1.00	female	87.50	no	no	cocodamol, cobenladopa, madopar, tamoxifen
yes	77.00	13.00	male	1100.00	no	no	rsagiline, entacampone, sinemet, co careldopa, simvastatine, thyroxine, carcadin
yes	70.00	14.00	male	605.00	yes	no	amantadine, co benaldopa, meformine, prampexole, co careldopa, alogliotin, amlodpine
yes	76.00	4.00	female	200.00	no	no	sinemet, madopar
yes	68.00	5.00	female	100.00	no	no	co careldopa, rivastigmine, lorazepam, clozapine
yes	57.00	12.00	female	400.00	yes	no	pramipex, madopar, opicapone
yes	85.00	7.00	male	797.00	no	no	sinement, madopar, stalevo
yes	75.00	2.50	male	0.00	no	no	drug naïve
yes	71.00	4.00	female	350.00	no	no	sinemet, amantadine, rasagiline, ramipril, cbd
yes	76.00	7.00	male	370.00	yes	no	amantadine, atorvastatin, entacapone, pramipexole, clonazepam
yes	70.00	10.00	male	1080.00	yes	yes	citalopram, co careldopa, colecalciferol, ropirinole
yes	55.00	4.00	male	500.00	no	no	madopar, opicapone, rivastigmine
yes	75.00	10.00	male	635.00	yes	no	sinemet, pramipexole, ropirinole
yes	61.00	5.00	male	575.00	yes	no	sinemet, ropirinole, atorvastatin, vitamins

Supplementary Table 2. Specific medication information for each patient enrolled in Study.

Power calculations for vMMN for the no-drug study (here reported as Study1, no drug effect, only difference in vMMN amplitude): we did both a one-tailed and a two-tailed test. We expect PD no VH to show a greater vMMN if compared to PDP patients, hence the one-tailed test. However, since there is no literature regarding vMMN in PD patients, we ran a two-tailed test as well, to take into account possible unexpected effects. We selected the effect size based on the literature on visual MMN in psychiatric disorders, based on differences in vMMN amplitude between an experimental and a control group[10]. For the one-tailed test, we entered an effect size of 0.75, α=0.05, power =0.8. with and Allocation ratio N2/N1 = 1.2 (assuming that we might have more PD no VH patients). Estimated sample size was 25 and 29 (total N = 60) for a power of .81. For the two-tailed test, we entered the same parameters as above, and the estimated sample size was 27 and 33 (N=58) for a power of .81. Due to the challenges and delays imposed to the pandemic we reached a N=38 of participants retained for Study 1.

During the EEG session, participants sat on a comfortable chair in a darkened, sound attenuated, and electrically shielded room, at a distance of 74-76 cm from the computer screen. Participants were fitted with a Compumedics Neuroscan 64-electrode EasyCap with sintered Ag-AgCl sensors and the SynAmps RT amplifier. We recorded vertical eye movements with two electrodes above and below the left eye, and horizontal eye movements with two electrodes at the outer canthi of the eyes, bilaterally. Signals were grounded to channel CZ, located at the midline. We also acquired signals from left and right mastoid (M1 and M2). Nevertheless, during the pre-processing, the signal was re-referenced to common average (see data analysis paragraph for details). Impedances of all electrodes were kept at 15kΩ or below. Signals were recorded using Scan 4.5 software. A sampling rate of 1000Hz was used for the recording. We used two EasyCaps for the study which were alternated across participants within each group.

Task. The task was a visual change detection task, adapted from Quian et al.[11]. Participants were given a joystick and were asked to pay attention to the fixation cross at the centre of the screen only and to press a button if the cross became smaller and another button if the cross became bigger. Participants were instructed to ignore the peripheral stimuli. The use of a concomitant visual task is recommended to minimize possible attentional effects in processing the stimuli designed to evoke the MMN and its relative simplicity was aimed to avoid exhausting participants[12]. Participants attended a practice run first, to familiarise with the task, consisting of 50 initial training standard only trials (6 with cross change). Participants were allowed to retake the practice trials if they felt they were not completely confident in starting the task. The experimental task (1000 trials) was divided in 2 blocks with a total of 44 trials where they cross changed in size. A two-minutes break was given between the blocks to allow participants to rest their eyes. The visual task was presented on a CRT monitor in the soundproof recording room; the monitor was connected to the experimental computer in a separate control room. The CRT monitor was chosen for its phosphor temporal characteristics to guarantee the best possible synchronisation between the presentation of the stimuli from the control room computer and the CRT

monitor, consistently with what found in the literature[13]. Using an oscilloscope, we verified that the delay between trigger and light sensor with VGA to CRT was of 6ms only (with LED monitor 13ms). We inverted the colours for the test to obtain a clean black to white signal, to check that the CRT produced a very immediate image, switching to full brightness immediately, while the LCD response ramped up in brightness. We also took a slow-motion video of the task running on the CRT monitor to check that it was truly v-synced, and we failed to see any partial images being drawn to the screen, which is a sign of good syncing.

The EEG task was programmed with *Presentation* with stimuli presented in a pseudo-randomized manner as the trial list was generated with a MATLAB script designed to create the task list according to the Poisson distribution. As it is required to elicit proper MMN and to avoid confounding processes being elicited, the stimuli differed only in the feature carrying the deviant information, thus orientation. The visual stimuli presented in the task were: a black cross (*Presentation* units height = 90; width = 9) positioned at the centre of the screen that became briefly smaller (*Presentation* units height = 45; width = 4.5) or bigger (*Presentation* units height = 180; width = 18), At the periphery, two flashing bars (*Presentation* units height = 450; width = 100), were presented with a 0 (rare deviant), 30 (frequent deviant) and 60 (standard) degree orientation. The bars for each orientation were simultaneously presented from a distance from the centre of the screen/cross of 400/-400 *Presentation* units, staying on screen for 50ms. The cross changes had a duration of 200ms. The stimulus onset asynchrony (SOA) was 600ms. To control for potential ERP differences related to the differences in the visual stimuli, we decided not to change the probabilities of standard and deviant stimuli across experimental blocks in order to keep the task under 15 minutes, as it would have been fatiguing for the patients. As the sample size did not allow to counterbalance the task across participants, we ran pilot sessions to make sure that the waveforms elicited by the standard and deviant stimuli did not differ (**Supplementary Information 5**) and then we picked one of the possible combinations as our task.

EEGlab preprocessing pipeline. For each participant, we removed VEO and HEO eye channels before high-pass filtering (lower edge of 0.1Hz and a higher edge of 30 Hz) to minimise the introduction of artefacts and to remove high-frequency noise. Data was re-referenced to the common average after this step. We made the decision to re-reference to common average because the signals from the mastoids was often not as good as initially hoped. For some of our participants the adherence of the electrode to the skin behind the ear was not stable thorough the recording, thus we decided to re-reference to average in order to have a better signal. We believe this was the best choice for our dataset to help standardise the data[14]. We used the *runica* algorithm to run independent component analysis on the data. After checking the ICA components with 2D component activations, maps and spectra, we removed those components that included eye blinks or motion artefacts. Data was once again re-referenced to the common average. After this step, an event list was created to divide the recording in standard, rare deviant and frequent deviant events. Data was epoched from 100 ms pre-stimulus to 500 ms post-stimulus onset and epochs were manually inspected and baseline correction was applied pre-stimulus. We rejected trials when the cross changed in size, when participants hit the joystick button, and bad epochs were rejected when necessary.

We retained a minimum of 100 trials for the deviant and a minimum of 550 for the standard. The epochs were averaged separately for standard and deviant stimuli in the three different conditions to produce the final averaged ERPs. This procedure was done separately for frontal (FPZ, FZ, F1:F8), parieto-occipital electrodes (OZ, O1, O2, PO1:PO8) and parietal electrodes (PZ, P1:P8). We exported MATLAB waveform plots to access peak amplitude data, together with .txt and ERP files for quantitative MMN data analysis. In addition, we divided the recordings in standard and rare deviant to be entered as datasets for an EEGlab study of the two different ERP components in PD vs. PD-VH as an additional way to analyse the differences between standard and deviant stimuli in the two groups.

Supplementary Information 5. *EEG pilots to ensure MMN was being measured in each possible condition and not a stimulus-specific waveform. The deviant is represented in pink and the standard in violet, as in the plots in the main text.*

Participants were: for Condition 1, P1 age = 28 student; Condition 2, P2 male age = 32, MS student; condition 3, P3 age = 24 university student. Participants 1-3 had normal vision; Participant 2 had corrected to normal vision.

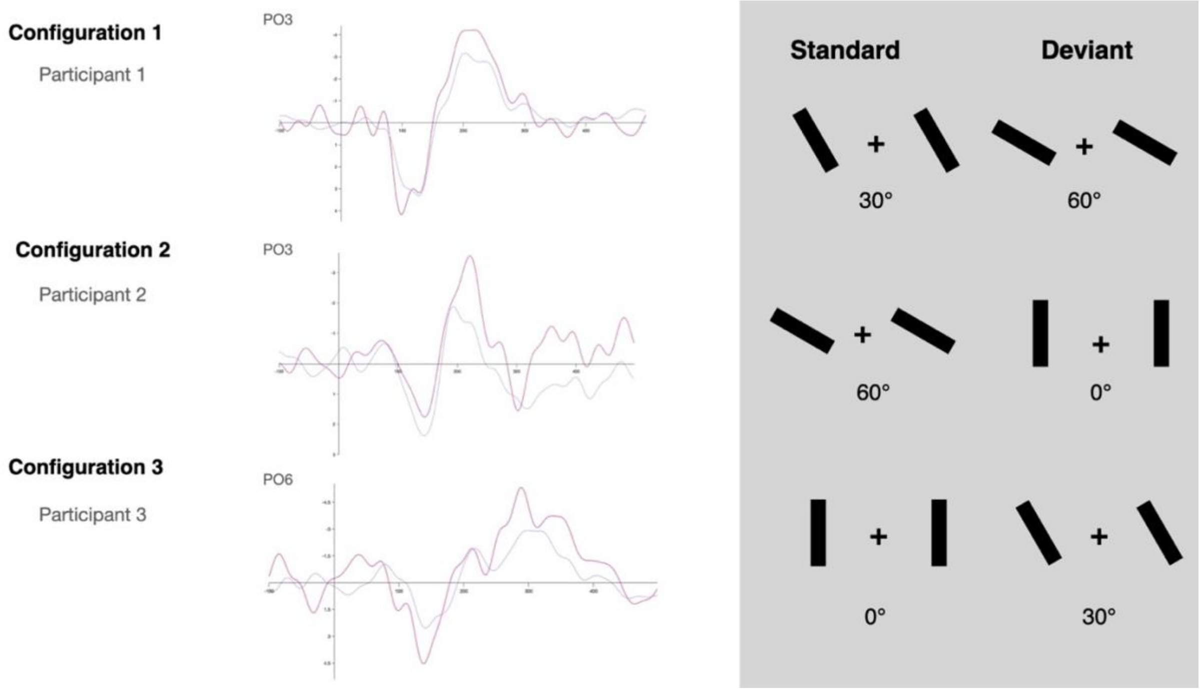

Supplementary Figure 4. *Example VEPs for each task configuration tested with the pilot.*

Supplementary Information 6. *Saracatinib and psylocibin behavioural study in healthy volunteers.*

One group of 23 healthy male volunteers participated in the study. As there was no information available on the effects of psilocybin and saracatinib on the developing foetus we took a conservative approach in excluding females. Participants were excluded if they had a personal history of psychiatric illness (assessed through a structured interview); first-order relative with a history of psychotic illness; evidence of cardiac, hepatic, renal, gastrointestinal or neurological disorders; excessive use of caffeine (> six cups of coffee per day or equivalent) and alcohol (> 28 units per week); current use of medication and failure of drugs of abuse test at screening or on a study day using a 10-panel test. Only participants with previous experience of hallucinogenic drugs were included in this study. No participant was a current regular user.

Three participants did not complete the study: the QTc reading of one participant's ECG exceeded the upper limit specified in the protocol on the day of testing; one participant experienced high anxiety prior to the psilocybin dosing on his first session and withdrew from the study (was in placebo arm) and one participant tested positive for cocaine on the morning of his second session. As such, 20 participants completed the study (mean age 26.6, SD 7.1, range 19 – 47).

This was a double-blind, placebo-controlled, cross-over, counter-balanced design for saracatinib. The drug is a SRC kinase inhibitor. SRC is a tyrosine kinase that plays a role in glucose metabolism regulation in cancer cells and had been used as a candidate therapeutic target in patients with solid tumours. Here we had evidence for brain penetration from rodent CSF after a single dose (AZ in-house data), although subsequently it was shown multiple days of dosing produces clear exposure and measurable effect on peripheral SRC kinase inhibition[15].

Eligible, consented participants attended two study days at least one week apart (mean 13.3 days, SD 3.5, range 7 – 15). Participants were randomised to receive an oral placebo followed by psilocybin or oral saracatinib 125mg followed by psilocybin. At approximately 240 minutes post-oral drug dose participants entered the scanner. The scanning session lasted 90 minutes, with a pump-controlled infusion of 2mg psilocybin in 10mL saline (prepared as a suspension and infused through a sterile filter) over 2 minutes occurring approximately 40 minutes into the scanning session.

The psychedelic experience was recorded using the top ten items from the questionnaire taken from a previous study[16], with the principle question of interest being "How intense were the drug effects when at their peak." One-tailed paired sample t-tests were used to analyse the data.

Supplementary Information 7. *PD-VH only saracatinib pilot study details and procedures.*

Power calculations. The numbers were estimated from a study using one of the fMRI tasks and EEG as co-primary endpoints. Following challenges and delays due to the pandemic, we recomputed power analyses for the EEG task for *N=12 for the vMMN* (difference between placebo and drug sessions). We carried out power calculations in *G*power*[17].

We used a repeated measures ANOVA, following the methodology used in a study with a double-blind, placebo-controlled, crossover design with 2 different drugs (S-ketamine, DMT) on 15 healthy volunteers[18]. Participants had a baseline MMN and the MMN task was repeated either 2 weeks or 4 weeks after each of the drugs. Effect size was 0.790. We input as $\alpha = .1$ and power$(1-\beta) = .9$, as we are aware that a sample size of N=12 will affect the power of the analysis. For the one group with two measurements (placebo/drug) and a hypothesized correlation of .5 between the repeated measures the effect size is estimate to be =.45.

We have explored the literature to compare our calculation inputs and outputs to studies using MMN (visual or auditory) in a double-blind design similar to ours and with relatively small sample sizes. No study of MMN with drug administration in Parkinson's disease has been found. A brief description of the relevant studies follows. Heekeren et al.[18]: auditory MMN paradigm in double-blind, placebo-controlled, crossover design with 2 different drugs (S-ketamine, DMT) on 15 healthy volunteers. Participants had a baseline MMN and the MMN task was repeated either 2 weeks or 4 weeks after each of the drugs. Effect size was 0.790. Juckel et al.,[19]: cannabis effect on auditory MMN on 22 healthy volunteers in a double-blind, placebo-controlled, crossover design, effect size of 0.8459. Umbricht et al.[20] auditory MMN in 20 healthy volunteers with a single-blind placebo-controlled design with ketamine to study cognitive deficits in schizophrenia. Effect size = 0.787. Schmidt et al.[21] ; auditory MMN, 19 participants taking ketamin, 20 participants psylocibin. Effect size =0.836 for ketamin vs. baseline. Fischer et al.[22] visual MMN in 27 healthy volunteers, double-blind. At the end of 2021, the drug stopped being manufactured, thus we stopped recruiting for the drug study, but the patients that were lined up for screening joined the non-drug study (Study 1). (More on this in the *Limitations* section in the main text).

Inclusion Criteria (criteria 1-7 were shared with the no-drug study)

To be eligible for participation in this study, the subject must:

1. Understand the study procedures and agree to participate by providing written informed consent.
2. Have a confirmed diagnosis of Parkinson's disease using internationally accepted UK brain bank criteria.
3. Be male or female
4. Be right handed
5. Aged 40 years or over
6. Have a score of at least 22 on the Montreal Cognitive Assessment (MoCA).
7. Have a diagnosis of idiopathic PD with moderate severity

8. Be judged to be in good health by the investigator, based on clinical evaluations including laboratory safety tests, medical history, physical examination, 12 lead ECG and vital signs measurements performed at screening and prior to administration of the initial dose of study drug.
9. Have a combined score of at least 6 or an individual score of at least 4 on the neuropsychiatric inventory (NPI [20]) 23 items A (delusions) and/or B (hallucinations).

Exclusion Criteria (criteria 1-9 were shared with the no-drug study)

The subject must be excluded from participating in the study if the subject:

1. Has an ongoing disability, medical or neurological history, cognitive impairment, or conditions that in the opinion of the investigator may interfere with study conduct or clinical assessments.
2. Has a hairstyle which would affect EEG recording.
3. Has a history of regular alcohol consumption exceeding 14 units/week (6 glasses of 13.0% wine (175ml), 6 pints of 4.0% lager or ale (568ml), 5 pints of 4.5% cider (568 ml) or 14 glasses of 10.0% spirits (25ml)) within 6 months of screening.
4. Uses tobacco- or nicotine-containing products in excess of the equivalent of 5 cigarettes per day.
5. Uses caffeine containing products of the equivalence of 5 cups of regular filter coffee per day
6. Is unwilling or unable to comply with the Lifestyle guidelines.
7. Has, in the opinion of the investigator, any medical or psychological condition or social circumstances which would impair their ability to participate reliably in the study, or who may increase the risk to themselves or others by participating.
8. Known to have tested positive for human immunodeficiency virus.
9. Participation in another clinical study with an investigational product administered in the last 3 months for the PDP cohort and 1 month for the PD cohort
10. Is a female of child bearing potential
11. Is currently taking anticholinergic medication.
12. Is currently taking any medication known to be a moderate or potent CYP3A4 inducer or inhibitor.
13. Refuses to be withdrawn from quetiapine.
14. Has a family history of psychosis in a first degree relative
15. Has poor peripheral arterial/venous access or recent wrist trauma that will restrict ability to gain venous access.
16. Is currently using prescription or non-prescription drugs and herbal supplements, which are deemed to affect the integrity of the study, within 7 days or 5 half-lives (whichever is longer) prior to the first dose of study medication. As an exception, paracetamol or acetaminophen may be used at doses of £1 g/day.
17. Has a history of sensitivity to any of the study medications or any of the excipient constituents.
18. Has a history of febrile illness within 5 days prior to the first dose
19. Has any condition possibly affecting drug absorption (eg, gastrectomy).

20. Has a positive urine drug screen on or after the screening visit during their active involvement in the study for opiates, methadone, cocaine, amphetamines (including MDMA), barbiturates, benzodiazepines and cannabinoids.
21. Is male and is unwilling to follow the contraception guidance or has a female partner of child bearing potential who is unwilling to follow the contraception guidance throughout the study.
22. Serum alanine aminotransferase (ALT) or aspartate aminotransferase (AST) $\geq$ 2.5 x upper limit of normal (ULN)
23. Total bilirubin $\geq$ 1.25 x ULN
24. Baseline resting QTcF > 470ms on 12 lead ECG
25. Positive hepatitis C antibody, hepatitis B virus surface antigen or hepatitis B virus core antibody at screening
26. Known congenital long QT syndrome
27. Below the lower limit of normal Hb, total WBC and neutrophils on blood counts as per the reference ranges of the laboratory conducting the tests.

*Experimental design and procedure***.** Patients were required to attend the study site for 5 visits: the initial screening visit, where patients were asked their medical history and underwent a physical and neurological exam and a vital observations check to make sure it was safe to administer the drug, and two visits for each of two study periods. On day 1 of each study period patients underwent baseline cognitive assessments to provide a profile for the patient and the physical exam and vital observation checks were repeated. The clinical study drug, *saracatinib,* an inhibitor of the Src/abl family of kinases, was supplied by AstraZeneca. *Saracatinib* was provided as 50mg pink film coated tablets in bottles containing 32 tablets to cover a potential 16 days of dosing together with matching placebo tablets.
The drug was administered by the study doctor, who was also tasked with discharging the patients after a 3 hour stay during which patients were regularly checked upon by the study team.
Participants took a daily oral dose of 100mg of Saracatinib at one study period and matched placebo at the other study period, in a randomised order not known to the study team. Unblinding was done after the EEG data were pre-processed and ERP data extracted. For each study arm, the drug was administered every morning for 14 days (+/- 2 days), as it is known that 10 days of dosing with 100 mg Saracatinib will achieve a steady state level that is known to be well tolerated in patients with Alzheimer's disease (Nygaard et al 2015). A minimum 14-day washout period between the final dose of period one and the first dose of period 2 was kept. Participants returned to the study site on day 14 to undergo the study assessments (EEG session, clinical and psychiatric questionnaires, physical and neurological examination). We also collected blood samples to measure pharmacokinetics for the drug. Samples were collected at day1 pre-dose, day14 pre- and post-dose. In some cases (see table) it was not possible to collect all the samples due to issues with patients blood pressure.

Samples were collected in 4 mL K2EDTA green top Becton-Dickinson Vacutainers and after processing stored in 1.8 mL cryogenic vials. Upon collection the tubes were gently inverted 8 to 10 times to mix the additive with the collected blood prior to centrifugation and placed immediately on ice. Vacutainers were centrifuged for 10 minutes at approximately 1100 to 1300 x g (RCF) at 4°C in a

pre-refrigerated centrifuge. Immediately following centrifugation, the plasma was removed from the packed cells and transferred into one (2 if the quantity allowed) pre-labelled 1.8 mL cryogenic vials. A minimum of 0.6 mL needed to be obtained for each aliquot. Plasma samples were frozen immediately at -20°C until shipment to the processing lab (Charles Rivers). No more than 45 minutes elapsed between blood collection and freezing the plasma sample.

Supplementary Information 8. *Parietal channels vMMN at early latencies (100-125 ms).* As we had hypotheses about parieto-occipital channels and frontal channels, but no strong hypotheses about parietal channels, we analysed vMMN at both 120-180ms and 100-125ms for these electrodes, finding that at later latencies there was a significant difference in the vMMN at P2 and a trend towards significance at PZ, reported in the main text. These results are congruent with the EEGlab study of standard vs. deviant waveforms. When analysed at the earlier latency where we found a vMMN at parieto-occipital channels, we did not find significant results at these channels. b) As some of the frontal electrodes showed a significance at the later latencies (~140-180ms) we also compared these differences in a shorter interval (140-180ms): F2 [$F(1,36) = 4.38$, $p = .04$] with PD-noVH MMN = 6.8 (sd 7.2) and PD-VH MMN= -0.83 (sd = 13.88), F4 [$F(1,36) = 5.02$, $p = .03$] with PD-noVH MMN = 6.9 (sd 7.6) and PD-VH MMN= -2.11 (sd = 15.24), F6 [$F(1,36) = 4.54$, $p = .04$] with PD-noVH MMN = 5.62 (sd 8.7) and PD-VH MMN= -3.61 (sd = 16.93), and AF4, [$F(1,26)=5.02$, $p = .03$] with PD-noVH MMN = 6.8 (sd 7.2) and PD-VH MMN= -0.83 (sd = 13.88).

Supplementary Information 9. *Individual VEPs for the standard stimuli for each participant involved in Study1.*

Supplementary Figure 5. *Individual VEPs for PD-noVH. The timing on the x axis are from 0 to 500 ms and are reported for 100ms, 200ms, 300ms, 400ms. The amplitude is reported with negative up as per standard convention.*

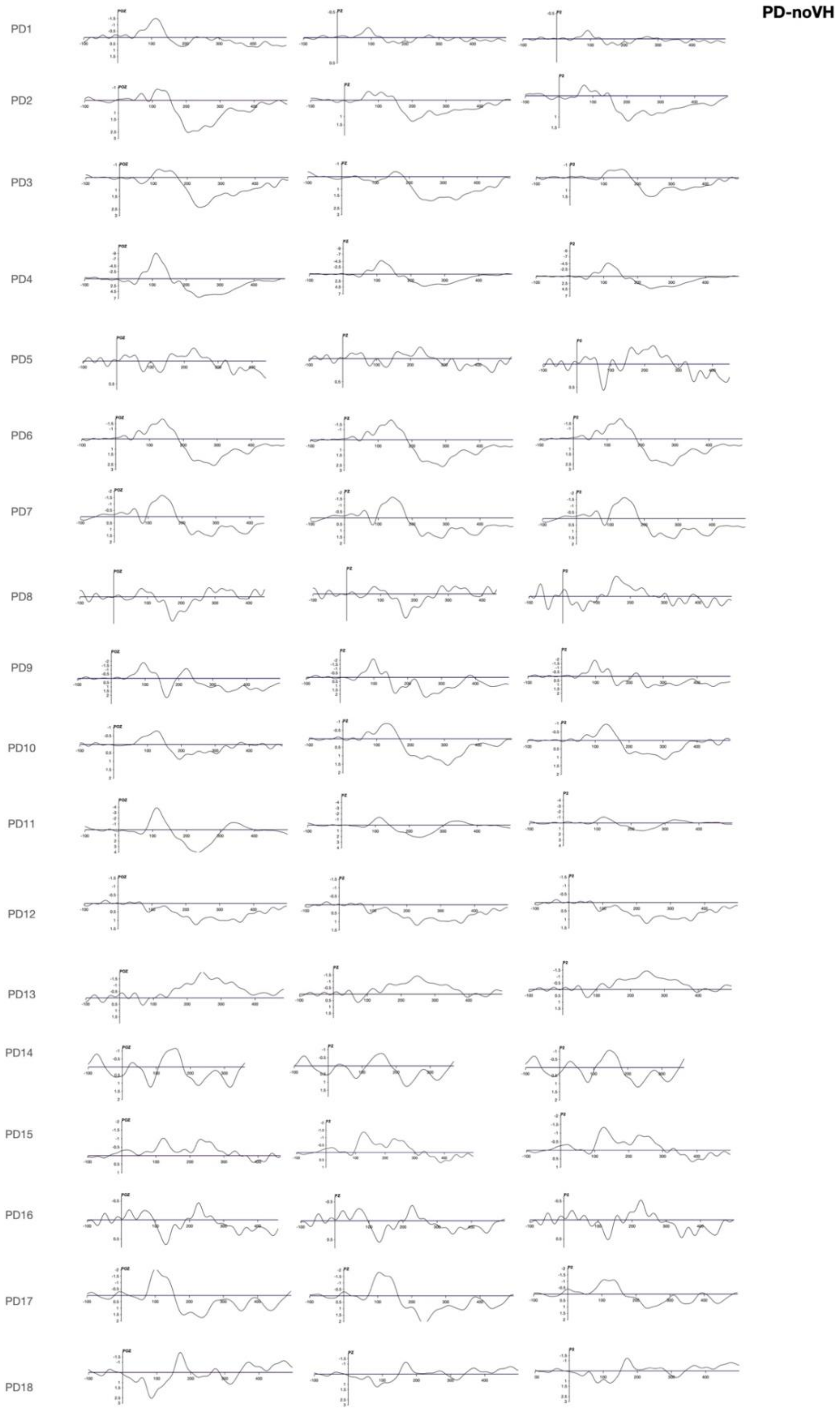

Supplementary Figure 6. *Individual VEPs for the standard stimuli for PD-VH. The timing on the x axis are from 0 to 500 ms and are reported for 100ms, 200ms, 300ms, 400ms. The amplitude is reported with negative up as per standard convention.*

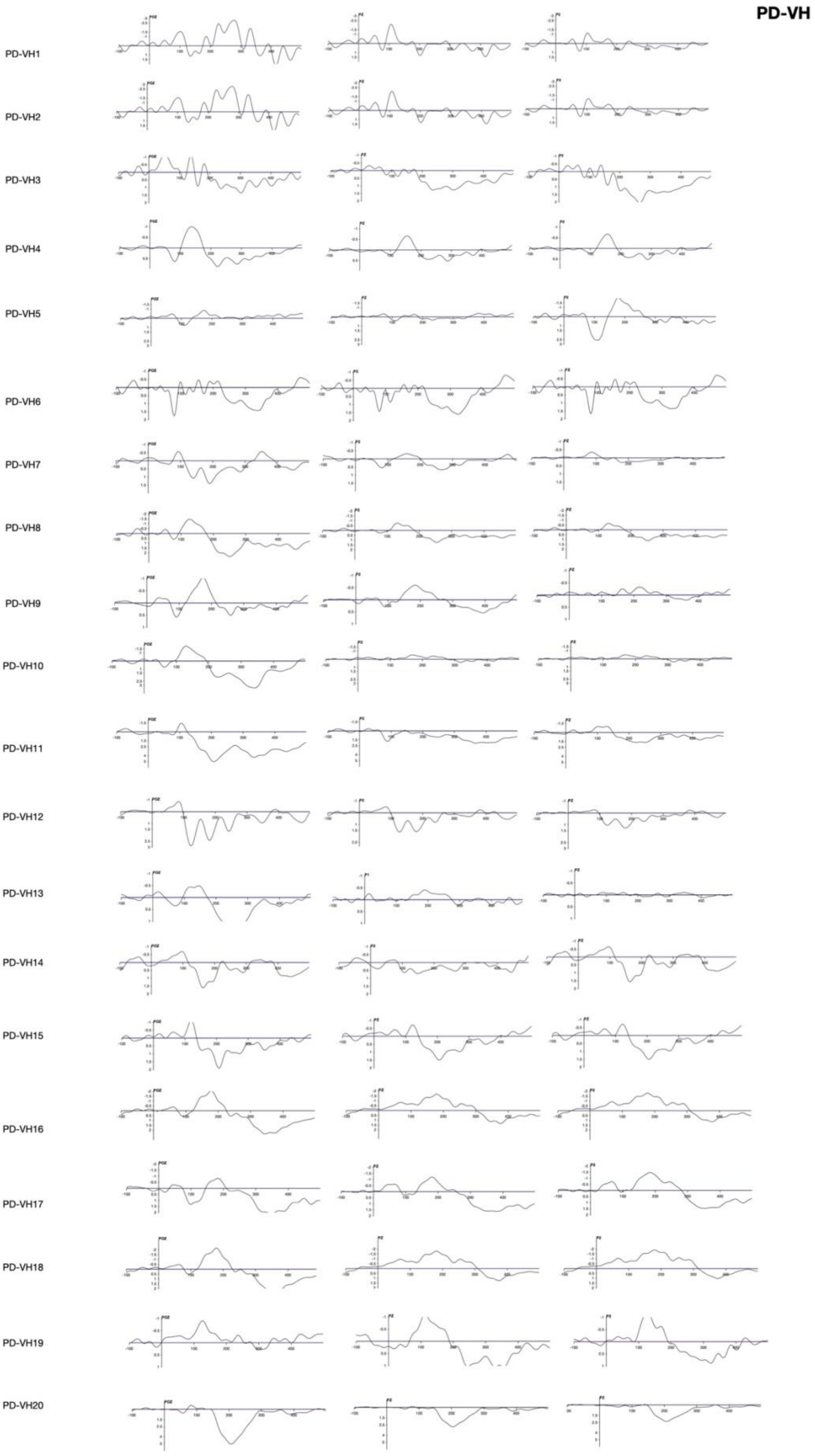

Supplementary Information 10. *Between group analysis of ERP components.* To further look at the differences between the groups and between standard and deviant, we also compared the groups with a 2x2 design in a EEGlab study structure: we used the same design for the within group analyses reported in the main text but standard/deviant was entered as condition and PD-VH/PD-noVH as group in a one-way ANOVA design. In the plots standard is violet and deviant is pink. EEGlab's STUDY structure was used for this exploratory between-subjects analysis. Epoched data was subject to a bin-based sub-setting in 'rare deviant' and 'standard' datasets for each participant. These datasets were entered in the model, with rare deviant/standard being entered as condition. A set of power spectra and event-related measures for each dataset were computed, with rare deviant/standard being entered as condition and PD-VH/PD-noVH as group in a one-way ANOVA design. Each dataset entered in the model is, with this step, associated with an ICA decomposition and thus a series of components. Each component has an equivalent dipole model, based on which pairwise distance measures between components can be then computed. Multiple comparisons across channels were false discovery rate corrected ($p < 0.05$).

Parieto-occipital channels. At POZ a significant difference is found for the condition in the latency of interest, surviving multiple comparison correction (*p*FDR <.05, see **Supplementary figure 7**). Similar, but weaker, results were observed for O1 and PO3, not surviving multiple comparison correction. A similar pattern is observed at parietal channels (PZ, P1 with FDR, P2), with a difference for condition throughout the presentation interval, an early interaction (stimulus onset) and a late (~350ms) group difference; a similar profile is found at P4, with an earlier group difference detected. A difference for condition is also found at P3 (~150-200ms).

Frontal channels. At FZ, comparing VH and noVH participants on the standard/deviant ERPs we find that there is a difference of condition at ~90, and at intervals between 120 and 170ms with an earlier (< 100ms) interaction, nevertheless both do not survive multiple comparison correction. A similar pattern is also observed for F2, F4, F5 and F6 (see **Supplementary figure 7** for an example), with interactions at F2 and F6, consistently with what we found in the analysis performed in R and reported in the previous section. FPZ and FP2 show a significant difference for condition at 140-160ms (*p*FDR <.05).

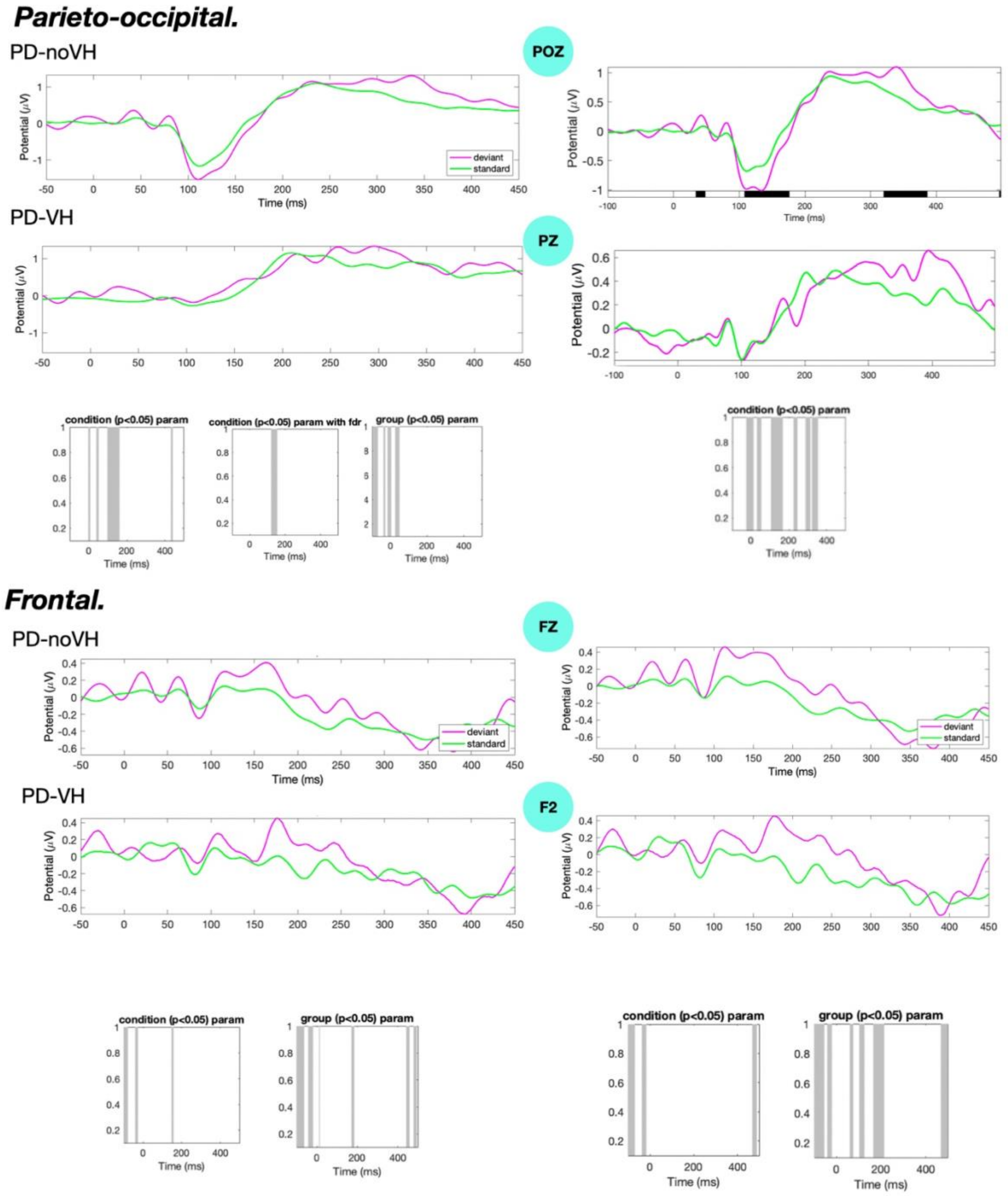

Supplementary ***Figure 7***. *Results of the 2x2 ANOVA with condition (rare, standard) and group (VH, noVH) described in this paragraph. Results are presented for the electrodes POZ and PZ (parieto-occipital) and FZ and F2 (frontal). These electrodes are among those mentioned in the results reported in the previous section.*

Supplementary Information 11. *Correlational analysis with the clinical variables and neuropsychiatric assessments (PD-VH only). NEVHI scores at baseline, placebo and drug,*

As reported in the main text, SAPS-PD and NEVHI were positively correlated (r=.59 p =.006). A significant correlation was found between levodopa equivalent daily dose (LEDD) and disease onset (r=.58 p =.01) and disease onset and SCOPA-motor score (r=.58 p =.007). No other significant correlations were found. The hallucinations score at SAPS-PD and NEVHI temporal severity for complex VH scores were positively correlated both when using the NEVHI continuous severity score (r = .57, p =.009) and the temporal score we created multiplying ordinal duration and ordinal frequency (r = .47, p =.035).

Supplementary Information 12. *PD-VH drug additional information.*

id	age	onset	LED	MoCA	SAPS PD	NPI	CSI Motor	CSI Disability	CSI. Mot compl	CSI. Cognitive	SCOPA-motor
Pt2	76	7	370	25	11	3	4	4	1	1	31
Pt3	70	10	1080	25	7	6	4	3	2	1	28
Pt4	55	4	500	22	23	4	1	3	1	2	19
Pt5	75	10	635	24	18	3	3	4	4	4	36
Pt7	61	5	575	30	10	6	1	1	0	0	11

10a. *Baseline clinical information on the participants of the drug study.*

Period	Pt2	Pt3	Pt4	Pt5	Pt7
Period 1 day1 pre-dose	<LLOQ<0.500	<LLOQ<0.500	<LLOQ<0.500	<LLOQ<0.500	<LLOQ<0.500
Period 1 day14 pre-dose	<LLOQ<0.500	54.8	<LLOQ<0.500		<LLOQ<0.500
Period 1 day14 postdose	<LLOQ<0.500	53.9	<LLOQ<0.500	<LLOQ<0.500	<LLOQ<0.500
Period 2 day1 pre-dose	<LLOQ<0.500	<LLOQ<0.500	<LLOQ<0.500		<LLOQ<0.500
Period 2 day14 pre-dose	114	0.577		61.8	85.8
Period 2 day14 postdose	111	<LLOQ<0.500	70.2	103	73.1

10b. *PK sample analysis.* grey cells = no drug period or day1 pre-dose, white cells = drug period. PT3 had the drug on period 1 and placebo on period 2, whereas the others were randomised to the opposite order.

10c. Wilcoxon paired tests revealed no difference between drug and placebo conditions also for pareidolia [$NEVHI_{PLA}$= 3.8 ± 4.15, $NEVHI_{DRUG}$= 5.4 ± 4.26, p =.2], presence [$NEVHI_{PLA}$= 1.2 ± 3.3, $NEVHI_{DRUG}$= 3 ± 3.3, p =.5] and passage [$NEVHI_{PLA}$= 1 ± 1.1, $NEVHI_{DRUG}$= 1.6 ± 1.6, p =1] VH.

The table shows the individual scores (P is for 'placebo').

	SAPS_P	SAPS_DRUG	NPI_P	NPI_DRUG	NEVHI_P	NEVHI_DRUG
Pt2	7.00	5.00	3.00	4.00	9.00	1.00
Pt3	9.00	3.00	1.00	2.00	7.00	8.00
Pt4	15.00	8.00	4.00	4.00	9.00	1.00
Pt5	10.00	8.00	13.00	11.00	6.00	6.00
Pt7	8.00	5.00	4.00	4.00	5.00	9.00

10d. *vMMN analyses at parieto-occipital channels.* At POZ, we find no significant difference between drug and placebo condition [Z = -.94, p= 0.35, $vMMN_{PLA}$= -0.46 ± 0.53, $vMMN_{DRUG}$= -0.22 ±0.74]. When carrying out the fixed effects analysis with POZ, PZ and OZ, we find similar results [Z = -.126, p= 0.9, $vMMN_{PLA}$= -0.29 ± 0.44, $vMMN_{DRUG}$= -0.33 ±0.63].

Supplementary Figure 8. *Individual waveforms in the placebo and drug conditions: Frontal electrodes. The plots were produced with EEGlavb: standard is depicted in green and the rare deviant pink.*

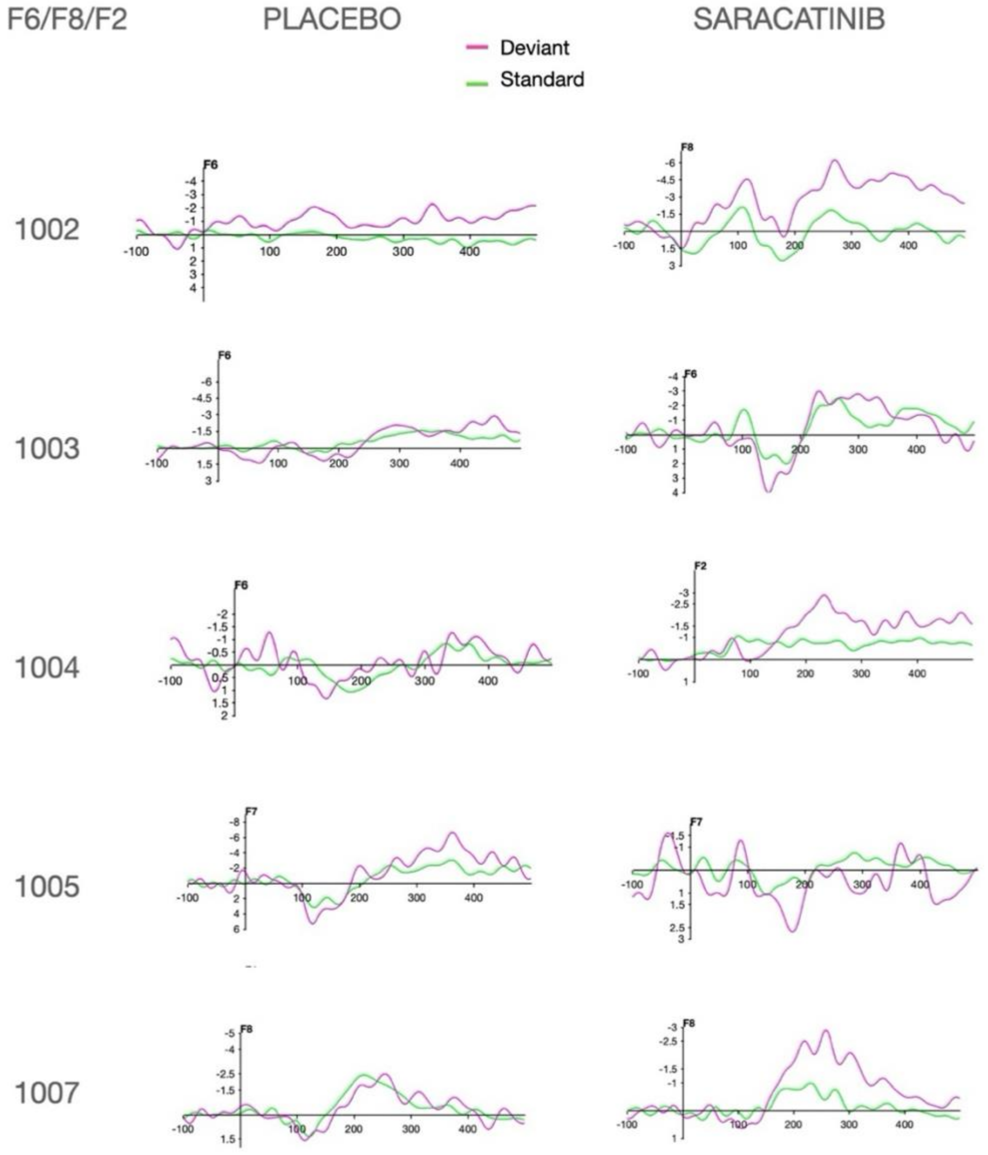

Supplementary Figure 9. *Individual waveforms in the placebo and drug conditions: Parieto-occipital electrodes. The plots were produced with EEGlavb: standard is depicted in green and the rare deviant in pink.*

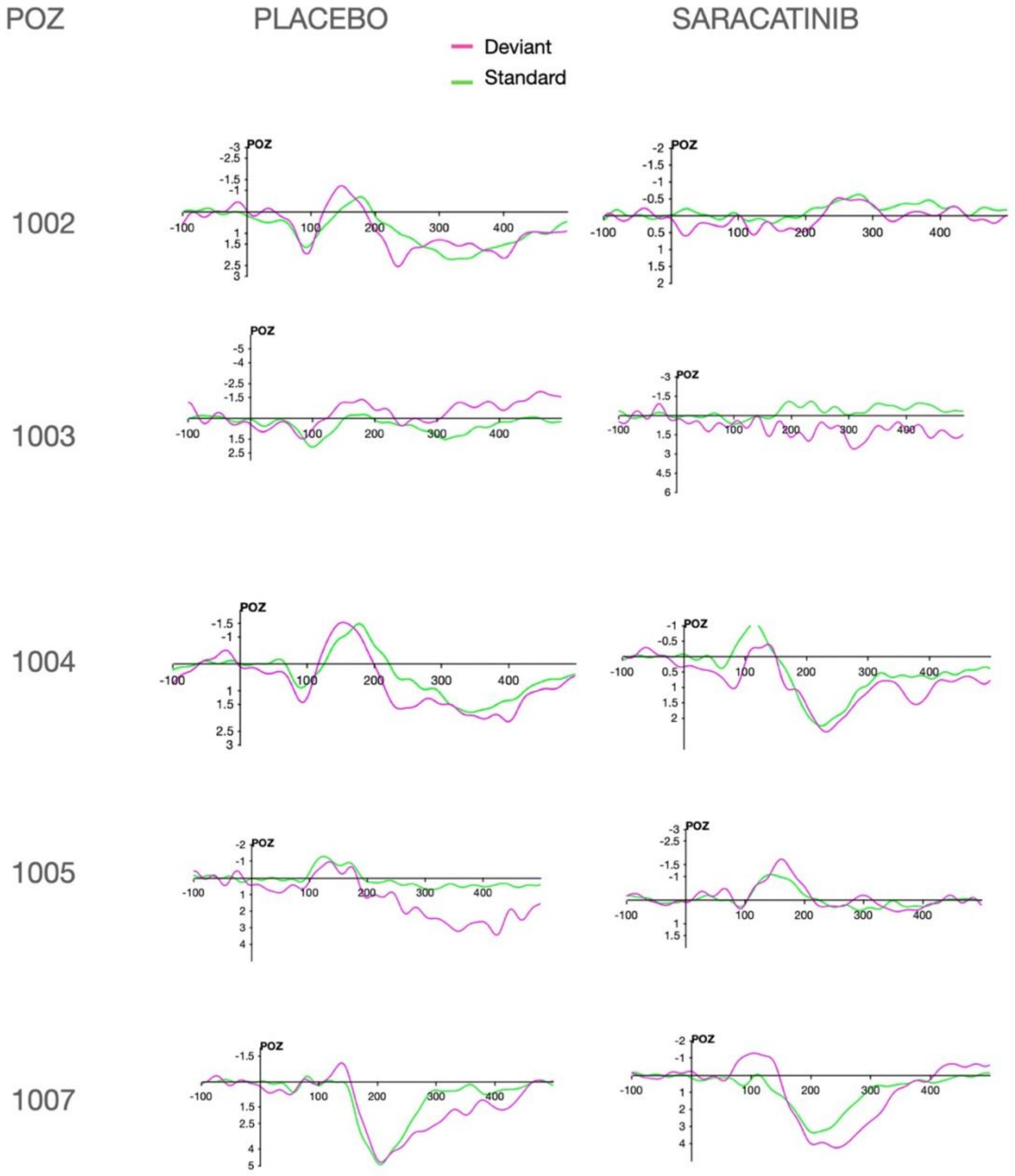

Supplementary Figure 10. *Individual waveforms in the placebo and drug conditions: Parietal electrodes. The plots were produced with EEGlavb: standard is depicted in green and the rare deviant in pink.*

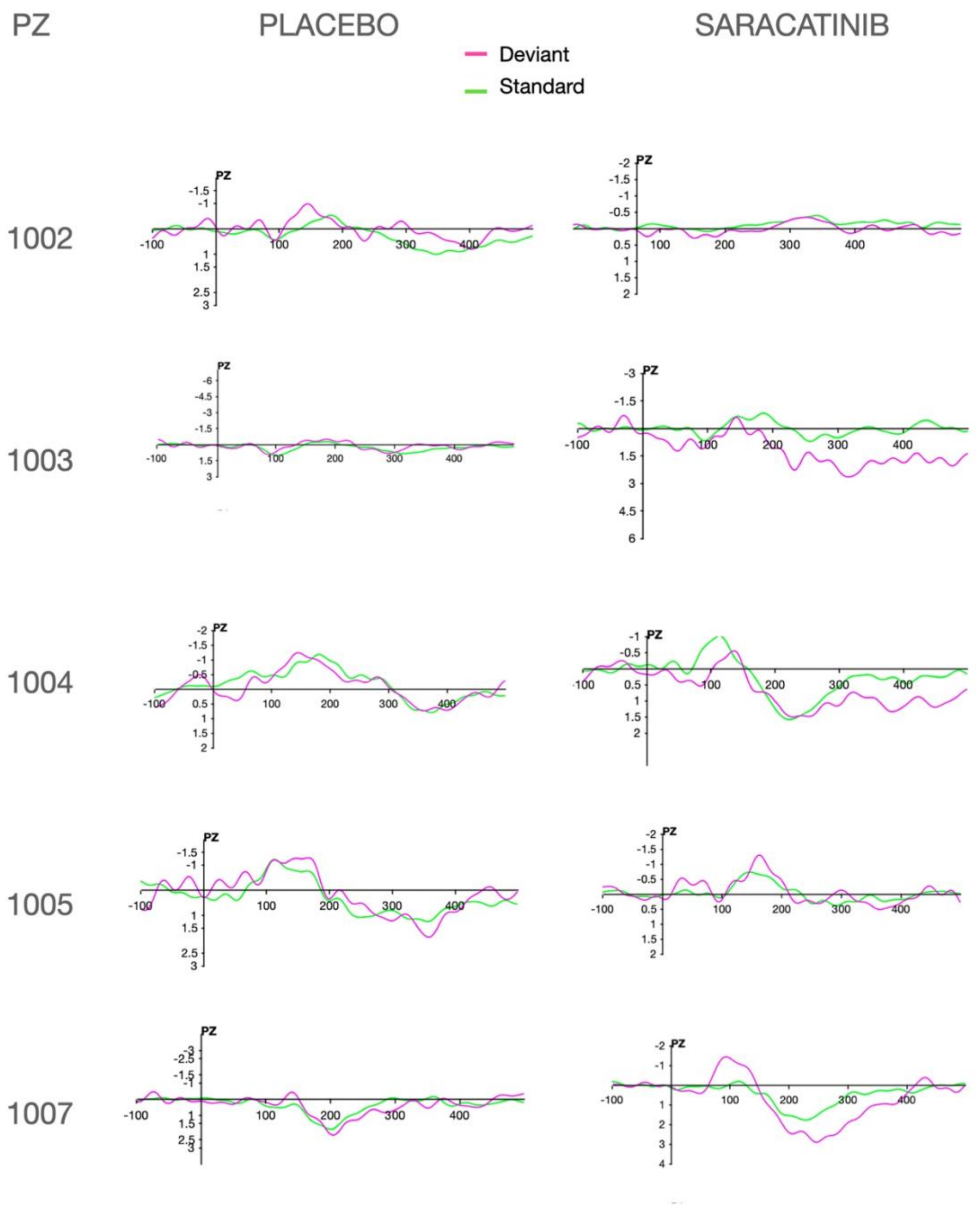

Supplementary Figure 11. *Study 1 and 3 flowchart*: we report numbers for all PD participants screened and allocated to the drug and non-drug arm of the study. The initially identified number included only participants reported as having hallucinations. Some of those patients eventually did not have hallucinations and were not allocated to the PDP group.

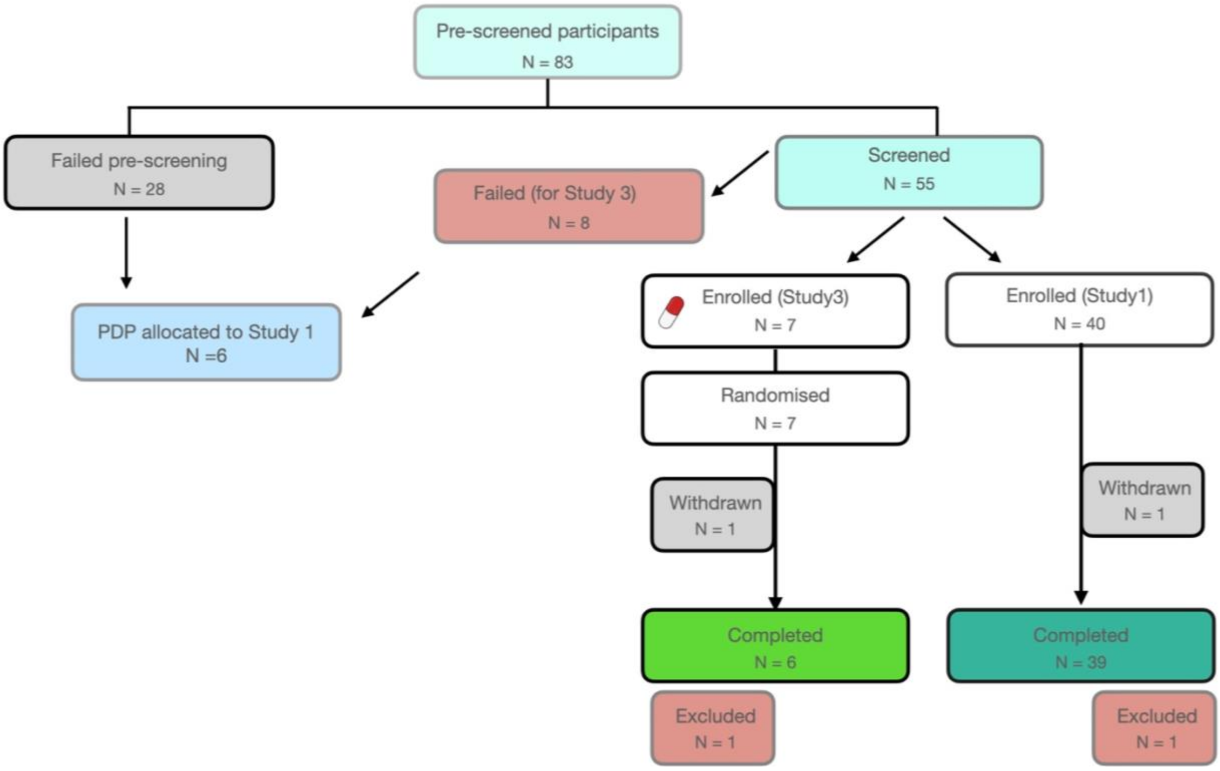

Supplementary References

1. Chaudhuri, K. R., & Martinez-Martin, P. (2008). Quantitation of non-motor symptoms in Parkinson's disease. *European journal of neurology*, *15*, 2-8.
2. Martinez-Martin P, Forjaz MJ, Cubo E, Frades B, de Pedro Cuesta J, et al. (2006) Global versus factor-related impression of severity in Parkinson's disease: A new clinimetric index (CISI-PD). *Mov Disord* 21: 208–214.M
3. Whitney, K. A., Shepard, P. H., Mariner, J., Mossbarger, B., & Herman, S. M. (2010). Validity of the Wechsler Test of Adult Reading (WTAR): Effort considered in a clinical sample of US military veterans. *Applied Neuropsychology*, *17*(3), 196-204.
4. Cummings JL, Mega M, Gray K, Rosenberg-Thompson S, Carusi DA, Gornbein J. The Neuropsychiatric Inventory: comprehensive assessment of psychopathology in dementia. *Neurology*. 1994;44(12):2308–2314. doi: 10.1212/WNL.44.12.2308
5. Voss, T., Bahr, D., Cummings, J., Mills, R., Ravina, B. & Williams, H. 2013. Performance of a shortened Scale for Assessment of Positive Symptoms for Parkinson's disease psychosis. *Parkinsonism Relat Disord*, 19**,** 295-9.
6. Mosimann UP, Collerton D, Dudley R, et al. A semi-structured interview to assess visual hallucinations in older people. *Int J Geriatr Psychiatry*. 2008;23:712–718. doi: 10.1002/gps.1965.
7. D'Antonio, F., Boccia, M., Di Vita, A., Suppa, A., Fabbrini, A., Canevelli, M., ... & Ffytche, D. (2022). Visual hallucinations in Lewy body disease: pathophysiological insights from phenomenology. *Journal of Neurology*, *269*(7), 3636-3652.
8. Fernandez, H. H., Aarsland, D., Fénelon, G., Friedman, J. H., Marsh, L., Tröster, A. I., ... & Goetz, C. G. (2008). Scales to assess psychosis in Parkinson's disease: critique and recommendations. *Movement disorders: official journal of the Movement Disorder Society*, *23*(4), 484-500.
9. Anwyl-Irvine, A. L., Massonnié, J., Flitton, A., Kirkham, N., & Evershed, J. K. (2020). Gorilla in our midst: An online behavioral experiment builder. *Behavior research methods*, *52*, 388-407.
10. Kremláček, J., Kreegipuu, K., Tales, A., Astikainen, P., Poldver, N., Näätänen, R., & Stefanics, G. (2016). Visual mismatch negativity (vMMN): A review and meta-analysis of studies in psychiatric and neurological disorders. *Cortex*, *80*, 76-112.
11. Qian, X., Liu, Y. I., Xiao, B., Gao, L. I., Li, S., Dang, L., ... & Zhao, L. (2014). The visual mismatch negativity (vMMN): Toward the optimal paradigm. *International Journal of Psychophysiology*, *93*(3), 311-315.
12. Stefanics, G., Kremláček, J., & Czigler, I. (2014). Visual mismatch negativity: a predictive coding view. *Frontiers in human neuroscience*, *8*, 666.
13. Husain, A. M., Hayes, S., Young, M., & Shah, D. (2009). Visual evoked potentials with CRT and LCD monitors: when newer is not better. *Neurology*, *72*(2), 162-164.
14. Nunez, M. D., Nunez, P. L., Srinivasan, R., Ombao, H., Linquist, M., Thompson, W., & Aston, J. (2016). Electroencephalography (EEG): neurophysics, experimental methods, and signal processing. *Handbook of neuroimaging data analysis*, *1*, 175-197.)
15. Nygaard, H. B., Wagner, A. F., Bowen, G. S., Good, S. P., MacAvoy, M. G., Strittmatter, K. A., ... & van Dyck, C. H. (2015). A phase Ib multiple ascending dose study of the safety, tolerability, and central nervous system availability of AZD0530 (saracatinib) in Alzheimer's disease. *Alzheimer's research & therapy*, *7*(1), 1-11.

16. Carhart-Harris, R. L., Williams, T. M., Sessa, B., Tyacke, R. J., Rich, A. S., Feilding, A., & Nutt, D. J. (2011). The administration of psilocybin to healthy, hallucinogen-experienced volunteers in a mock-functional magnetic resonance imaging environment: a preliminary investigation of tolerability. *Journal of psychopharmacology*, *25*(11), 1562-1567.
17. Faul, F., Erdfelder, E., Lang, A. G., & Buchner, A. (2007). G* Power 3: A flexible statistical power analysis program for the social, behavioral, and biomedical sciences. *Behavior research methods*, *39*(2), 175-191.
18. Heekeren, K., Daumann, J., Neukirch, A., Stock, C., Kawohl, W., Norra, C., ... & Gouzoulis-Mayfrank, E. (2008). Mismatch negativity generation in the human 5HT 2A agonist and NMDA antagonist model of psychosis. *Psychopharmacology*, *199*(1), 77-88.
19. Juckel, G., Roser, P., Nadulski, T., Stadelmann, A. M., & Gallinat, J. (2007). Acute effects of Δ9-tetrahydrocannabinol and standardized cannabis extract on the auditory evoked mismatch negativity. *Schizophrenia research*, *97*(1-3), 109-117.
20. Umbricht, D., Schmid, L., Koller, R., Vollenweider, F. X., Hell, D., & Javitt, D. C. (2000). Ketamine-induced deficits in auditory and visual context-dependent processing in healthy volunteers: implications for models of cognitive deficits in schizophrenia. *Archives of general psychiatry*, *57*(12), 1139-1147.
21. Schmidt, A., Bachmann, R., Kometer, M., Csomor, P. A., Stephan, K. E., Seifritz, E., & Vollenweider, F. X. (2012). Mismatch negativity encoding of prediction errors predicts S-ketamine-induced cognitive impairments. *Neuropsychopharmacology*, *37*(4), 865.
22. Fisher, D. J., Scott, T. L., Shah, D. K., Prise, S., Thompson, M., & Knott, V. J. (2010). Light up and see: enhancement of the visual
