# Supplementary material for: Visual mismatch negativity in Parkinson's psychosis and potential for testing treatment mechanisms
Source: Brain Commun. 2024 Sep 3;6(5):fcae291. doi: 10.1093/braincomms/fcae291 (PMC11443450; doi:10.1093/braincomms/fcae291)
Supplement: fcae291_Supplementary_Data [file Original_submission_decision_letter.pdf › Original_submission_decision_letter.pdf]

Decision Letter One: Original Version

04-Mar-2024

Dear Dr. Vignando,

Thank you for your recent submission BRAINCOM-2023-567 entitled "Visual mismatch negativity in Parkinson’s psychosis and potential for testing treatment mechanisms" to Brain Communications.

Your manuscript has been reviewed, and while the reviewers and editorial team appreciate many aspects of the paper, they raise some concerns that you will need to address in order for the paper to be considered for publication. As you will see the reviewers raise some criticisms about the study design and methods, among other issues.

If you can address these concerns, I invite you to submit a revised manuscript and a response to each of the reviewers' comments. The reviewers’ comments are included at the bottom of this message.

In addition to the reviewers' comments, please also address our Scientific Editor's comments added below to comply with our requirements for presentation of scientific data at Brain Communications.

Please format your revised manuscript according to our Instructions to Authors
https://academic.oup.com/braincomms/pages/General_Instructions
A summary of key points is attached to help with this. Please note that there can be substantial delays to publication if you don't resubmit in line with our requirements.

To revise your manuscript, click the link below or log into https://mc.manuscriptcentral.com/braincom and enter your Author Centre, where you will find your manuscript title listed under "Manuscripts with Decisions." Under "Actions," click on "Create a Revision." Your manuscript number will be appended to denote a revision.

*** PLEASE NOTE: This is a two-step process. After clicking on the link, you will be directed to a webpage to confirm. ***

https://mc.manuscriptcentral.com/braincom?URL_MASK=099fc62d175b44b7ba659ed ad90395ea

Please upload a revised manuscript with changes tracked with track changes or in coloured text.

When submitting your revised manuscript, you will be asked to respond to the reviewer(s) comments. Please be as specific as possible in your response to all of the comments.

The editorial team at Brain Communications aims to increase transparency in scientific publishing and to demonstrate the value that peer review brings to the scientific process. Please therefore consider whether you are willing to have reviewer(s)' comments on your paper and your response published as supplementary data with the paper if it is accepted. This is entirely optional and will only be done if both the authors and reviewers agree. While submitting your revision there will be an opportunity to review or update the answer you gave previously, if necessary.

IMPORTANT: Your original files are available to you when you upload your revised manuscript. Please delete any redundant files before completing the submission.

Because we are trying to facilitate timely publication of manuscripts submitted to Brain Communications, we would appreciate your revised manuscript as soon as possible. If it is not possible for you to resubmit within 3 months, the option to submit a revision will expire, and we would then consider your revised paper as a new submission. However, if you need a little more time, please get in touch and we can extend this deadline for you.

Once again, thank you for submitting your manuscript to Brain Communications and I look forward to receiving your revision.

Best wishes

Dr. Alberto Lleo
Associate Editor, Brain Communications

Professor Tara Spires-Jones
Founding Editor, Brain Communications

Reviewers' Comments to Author:

Reviewer: 1

Comments to the Author
Miriam Vignando and colleagues present a research manuscript entitled: “Visual mismatch negativity in Parkinson’s psychosis and potential for testing treatment mechanisms”, which is a neurophysiological scalp EEG analysis study reporting reduced mismatch negativity (MMN) in 20 Parkinson´s disease patients suffering from visual hallucinations (PD-VH). A separate part of the study tests also the potential role of serotoninergic cascade in PD visual hallucinations in a small group of 5 subjects. The manuscript is well written, the methodology and statistics used are appropriate, the main finding is important. However, I have some comments.

Major comments:
1/ In the Introduction and Discussion sections the possible serotoninergic mechanism in visual hallucinations production in PD patients is described. This mechanisms might be also involved. However, it is well known, that dopaminergic medication, mainly dopamine agonists, play the major role in PD related psychosis. This should be, at least, discussed.
2/ LEDD equivalent is provided only. According to the nature of this study it is important to show full medication the patients had (dopamine agonist? SSRI?). Subjects with visual hallucination probably used chronic antipsychotic medication (quetiapine?).
3/ It is not clear, if the EEG measurements were performed in “off” or “on” dopaminergic medication state. Again, it is well known, that levodopa influences the EEG in PD substantially.

Minor comments:
4/ It is not clear why the MDS-UPDRS scores are not provided.
5/ A basic description of the EEG hardware and configuration (e.g. number of channels, sampling, reference) should be mentioned in the main text, not only in supplementary materials.
6/ The description of the EEG methodology in the main text sounds more like the manual for configuring the EEGLAB so some steps don't have to be clear. Please rewrite it in a more general form - similar to the methodology description used in the supplementary material.
7/ Sometimes it is confusing how the multiple comparisons corrections are used (across channels?,...). Please explain this in the text more clearly.
8/ What is the reason for using two different multiple comparisons correction methods (FDR, Benjamini-Hochberg)?

9/ Reporting the statistics in study 3 for 5 subjects only is questionable.
10/ In scalp topography comparisons (results in Figure 4) the topography is always a mean of time points from mentioned interval?
11/ Results in Figure 3. - how the statistics along the time axis was calculated? For each datapoint individually?
12/ There is a mistake in Figure 5- both columns are marked as PD-non VH.

Reviewer: 2 - Dr. Jaime Kulisevsky, assisted by Dr. Saül Martínez-Horta

Comments to the Author

Vignando and collaborators present interesting work exploring the morphology of the MMN in the context of PD, with and without visual hallucinations, using this component of the ERPs as an indicator of the integrity of the systems dedicated to detecting sensory-level changes related to error prediction.

Conceptually, I find the idea they present and justify throughout the introduction very interesting and well-crafted.

The selection of the population and the characteristics of the sample seem appropriate. Similarly, the experiment conducted with Saractinib appears equally suitable for testing some of the hypotheses proposed.

In Study 1, I consider it relevant to provide a detailed description of the phenomenology of the patients' hallucinations or at least characterize whether they presented minor or major/structured hallucinations.

I understand that the authors might argue, based on Figure 3, that it is somewhat difficult to affirm whether the signal obtained in the PD-VH group reflects the expected morphology of an MMN or an ERP obtained after performing a grand average. Essentially, this point defines one of the biggest limitations I have detected, since, regardless of the statistics, it is difficult for me to assume that these graphs show an ERP. I believe that to validate these records, it would be necessary to show the morphology of other ERPs where it is not foreseeable that the presence or absence of VH has had a significant effect. For example, it would be appropriate to show in both groups the morphology and topography of the VEP elicited during the task execution.

In the description of the methods used for EEG/ERP analysis, I would like the authors to specify why they chose to re-reference the recording to an average reference. At the

same time, it is not described whether any baseline correction was applied, something that is essential. Likewise, after applying methods related to artifact correction, it would be necessary to indicate the number of epochs used for analysis in each group to confirm a homogeneous distribution between groups in terms of available epochs.

I also consider it relevant for the authors to justify under what assumptions they believe that a lower MMN translates into an alteration.

In the discussion, it seems relevant to consider that some studies have emphasized the role of the attentional commitment component in patients with PD-VH. Specifically, we know that these patients show a predisposition in the ventral attentional system toward the evaluation of peripheral stimuli, thus favoring the interference of these stimuli over the target stimuli in a given task. In this sense, it is worth considering to what extent the reduction of MMN is a direct consequence of dysfunction in the systems dedicated to generating this component versus a consequence of the fact that the MMN-eliciting stimuli were not processed covertly but rather facilitated through attention mediated by the ventral attentional system. It would be interesting to show, in this sense, the possible effects in the time interval corresponding to P3a and P3b in both groups.

Considering that the MoCA is not an instrument capable of detecting relatively subtle cognitive changes, but that the PD-VH group scores higher in inattention, I consider it relevant to consider the possible effect on the morphology of the ERPs caused by the fact that patients with OAB have greater cognitive impairment. Something that, on the other hand, depending on the type of hallucinations, is predictable.

Regarding the studies with Saractinib, given the very limited number of cases included, perhaps the work is somewhat detracted, and it may be necessary to consider not incorporating this analysis into this publication. It may be worthwhile to better address the issues regarding MMN between patient groups and perhaps wait until data from more cases are available to present the results of Saractinib.

Comments from the Scientific Editorial team:

-Please supply a graphical abstract. A graphical abstract is a visual representation of the central finding or methodology of the paper. It will be displayed in the table of contents. Authors should strive to make the graphical abstract informative, interesting, visually appealing, and straightforward (no caption needed, no abbreviations except gene names). The technical requirements for the graphical abstracts are:
- Size: 120 x 120 mm (300-600dpi) or 3000 x 3000 pixels
- Font: Arial 12-16 pt

- File type: tiff or PDF
- Aspect ratio: 1:1 preferred

-Please add an Abbreviated Summary (up to 50 words) of your paper that captures the main purpose and conclusions of the work. The summary should state the main results written in the third person ('[1st author surname] et al. report that ...') and conclusion. This summary will be used in the contents list online and should not contain any abbreviations other than those on the accepted abbreviations list and accepted gene/protein names, making it accessible to the non-specialist. Please upload it as a Word document. Allowed abbreviations: AIDS; ANOVA; AMPA; ATP; A,T,C,G; CNS; CSF; CT; DNA; ECG; EEG; EMG; GABA; HIV; MRI; NMDA; PET; PCR; RNA.

-Thank you for providing a checklist. For all sections, please include the page numbers corresponding to the items listed in the reporting guideline checklist.

-Thank you for providing a paragraph describing statistical analyses. Please label the paragraph with a subheader 'statistical analysis' in the 'Materials and Methods' section.

-Data availability: Thank you for providing a data availability statement. If applicable, please provide the codes generated and used in this work within the supplementary material or through a repository, whose link has to be made available within the data availability statement.

-Figures 1 and 2: Please ensure that all abbreviations are spelt out in the figure legend.

-Figures 2 and 6c, Supplementary figure 2b legends: Please provide details of the statistical analysis performed, the experimental unit (n= mice, cells, wells, plate downs, etc)) and the number of data-points for each group analysed in these plots, and if the number is <10 show the single data-points. Please note our policy on pseudoreplication, we do not accept analyses where individual cells are the experimental unit when there is likely biological variability between animals, culture replicates, etc. Please see instructions for authors for more details. For this type of data, we require either that a mean/median be taken per animal/culture replicate followed by statistical analyses or that more sophisticated analyses are performed such as mixed effects modelling which can account for random variables.

-Figures 3, 4, 5, 6b and 7, Supplementary figures 3, 8a, 8b, 10e legends: Please provide details of the statistical analysis performed, the experimental unit (n= mice, cells, wells, plate downs, etc).

-Figure 4: Please revisit your colour choice in the figure as the green-red combination is not colour-blind friendly.
