# Supplementary material for: Visual mismatch negativity in Parkinson's psychosis and potential for testing treatment mechanisms
Source: Brain Commun. 2024 Sep 3;6(5):fcae291. doi: 10.1093/braincomms/fcae291 (PMC11443450; doi:10.1093/braincomms/fcae291)
Supplement: fcae291_Supplementary_Data [file Original_submission_Response_to_reviewers_comments.pdf › Original_submission_Response_to_reviewers_comments.pdf]

Response to Reviewers: Original Version

We wish to thank both reviewers for the attention and time given to our manuscript and the insightful comments.
Below we provide a point-by-point response to all the comments. All the changes in the main text are highlighted in red and in the Supplementary Information file in violet.

Reviewers' Comments to Author:

Reviewer: 1

Comments to the Author
Miriam Vignando and colleagues present a research manuscript entitled: “Visual mismatch negativity in Parkinson’s psychosis and potential for testing treatment mechanisms”, which is a neurophysiological scalp EEG analysis study reporting reduced mismatch negativity (MMN) in 20 Parkinson´s disease patients suffering from visual hallucinations (PD-VH). A separate part of the study tests also the potential role of serotoninergic cascade in PD visual hallucinations in a small group of 5 subjects. The manuscript is well written, the methodology and statistics used are appropriate, the main finding is important. However, I have some comments.

Thank you for the detailed and interesting comments. Please find a point-by-point reply addressing all the comments.

Major comments:
1/ In the Introduction and Discussion sections the possible serotoninergic mechanism in visual hallucinations production in PD patients is described. This mechanisms might be also involved. However, it is well known, that dopaminergic medication, mainly dopamine agonists, play the major role in PD related psychosis. This should be, at least, discussed.

Thank you for pointing this out. The reviewer is of course correct that dopaminergic medication might be involved to some extent in PD psychosis; and we now mention this in the introduction at page 3, now reading “Dopamine agonists play a role in these symptoms, but are not the only factor, as different studies show that up to 42% of drug naïve patients show forms of visual hallucinations, with percentages ranging from 12% to 26% to 42% (for a review see Chang and Fox, 2016). These results suggest that despite the involvement of such medication, this cannot be the only mechanism underlying this cluster of symptoms”.

2/ LEDD equivalent is provided only. According to the nature of this study it is important to show full medication the patients had (dopamine agonist? SSRI?). Subjects with visual hallucination probably used chronic antipsychotic medication (quetiapine?).

Apologies for not clarifying this earlier. We had added inclusion and exclusion criteria in the SI but we have not specified in the main text that none of our patients were on quetiapine. We have added a supplementary table with the specific medications our patients were being treated with. We use a chi square statistic to compare PD and PDP on dopamine agonist use, finding no difference between the groups, now reported in the main text.

None of the patients in the drug study was on SSRIs, as we were studying the effects of saracatinib on the 5-HT2A cascade this was an exclusion criterion. The criteria for the non-drug study allowed antidepressant medication and one PDP (sertraline) and one PD (citalopram) were on SSRIs. One PDP patient was on clonazepam for sleep, one on clozapine. One PD patient on lamotrigine, and another one on clonazepam for sleep issues. The information has been added in the main text.

3/ It is not clear, if the EEG measurements were performed in “off” or “on” dopaminergic medication state. Again, it is well known, that levodopa influences the EEG in PD substantially.

Patients were “on”. We have now specified this, thanks for pointing this out.

Minor comments:
4/ It is not clear why the MDS-UPDRS scores are not provided.

The protocol was drafted along with the PD neurologist specialist. Our assessments included the SCOPA-motor and CISI-PD. The choice was also determined by the fact that MDS-UPDRS does not provide a good measure of hallucinations and delusions, which were the focus of our study and based on the fact that patients had to undergo a long battery of psychiatric and cognitive assessments, thus the combined use of SCOPA-motor and CISI-PD was considered a good option to estimate the extent of motor symptoms. We now mention in the discussion a limitation is qualitative comparison with other studies.

5/ A basic description of the EEG hardware and configuration (e.g. number of channels, sampling, reference) should be mentioned in the main text, not only in supplementary materials.

We have moved to the main text some of the information keeping it brief as not to exceed the word count.

6/ The description of the EEG methodology in the main text sounds more like the manual for configuring the EEGLAB so some steps don't have to be clear. Please rewrite it in a more general form - similar to the methodology description used in the supplementary material.

The motivation for the EEG methodology was to provide sufficient detail to allow replication. We have moved the more detailed description to the SI and have reworked the section accordingly.

7/ Sometimes it is confusing how the multiple comparisons corrections are used (across channels?,...). Please explain this in the text more clearly.

For vMMN we used multiple comparisons correction across channels, for the EEGlab analysis multiple comparisons were used across channels and for every time point in the interval analysed, and we have now specified it in the text and figure descriptions.

8/ What is the reason for using two different multiple comparisons correction methods (FDR, Benjamini-Hochberg)?

We used FDR correction using the Benjamini-Hochberg method.

9/ Reporting the statistics in study 3 for 5 subjects only is questionable.

We have provided the statistics so the reader can make their own assessment of the data, but we do indicate that the numbers are a limitation when presenting the statistics and in the discussion.

10/ In scalp topography comparisons (results in Figure 4) the topography is always a mean of time points from mentioned interval?

Yes, that's correct. We have now added in the figure legend "scalp topography is averaged across the interval presented".

11/ Results in Figure 3. - how the statistics along the time axis was calculated? For each

datapoint individually?

Yes, that’s correct.

12/ There is a mistake in Figure 5- both columns are marked as PD-non VH.
Thank you for spotting this. We have now amended the figure.

Reviewer: 2

Comments to the Author

Vignando and collaborators present interesting work exploring the morphology of the MMN in the context of PD, with and without visual hallucinations, using this component of the ERPs as an indicator of the integrity of the systems dedicated to detecting sensory-level changes related to error prediction.

Conceptually, I find the idea they present and justify throughout the introduction very interesting and well-crafted.

The selection of the population and the characteristics of the sample seem appropriate. Similarly, the experiment conducted with Saractinib appears equally suitable for testing some of the hypotheses proposed.

In Study 1, I consider it relevant to provide a detailed description of the phenomenology of the patients' hallucinations or at least characterize whether they presented minor or major/structured hallucinations.

The detailed description of the phenomenology is presented in Figure 6 and the SI. Specifically, we have provided in SI1b all NEVHI scores reported per each scale, with duration of hallucinations reported in seconds, frequency in how many experiences per type are experienced in a typical month; average scores are reported for distress and frustration ratings (rated on a scale from 0 to 10). In the brackets we report how many participants had the symptom. We also report the % of participants for insights and delusions are reported: we included in the calculations those who had a certainty the VH was real at the time they occurred; 2 more participants stated it might happen at times.
In SI1c we also report how from raw scores of duration (seconds) and frequency (how many experiences in a month) we have converted each to ordinal scores to create the

multiplicative factor that we used as a measure of temporal severity. We also provide a mosaic plot of the proportion of type of visual hallucination as examined by the NEVHI in Figure 6 (including: complex VH, visual illusions (specifically pareidolia), passage, presence, and simple). Because of the word limit and our desire to provide a detailed description of the phenomenology we have provided this information in the SI.

I understand that the authors might argue, based on Figure 3, that it is somewhat difficult to affirm whether the signal obtained in the PD-VH group reflects the expected morphology of an MMN or an ERP obtained after performing a grand average. Essentially, this point defines one of the biggest limitations I have detected, since, regardless of the statistics, it is difficult for me to assume that these graphs show an ERP. I believe that to validate these records, it would be necessary to show the morphology of other ERPs where it is not foreseeable that the presence or absence of VH has had a significant effect. For example, it would be appropriate to show in both groups the morphology and topography of the VEP elicited during the task execution.

We agree that the PD-VH group have a grand average waveform that does not look like a VEP. We have provided an additional supplementary information with VEP for the standard for each individual participant for both groups, in Supplementary information 8a.

In the description of the methods used for EEG/ERP analysis, I would like the authors to specify why they chose to re-reference the recording to an average reference.

We made this decision because the signals from the mastoids was often not as good as initially hoped. For some of our participants the adherence of the electrode to the skin behind the ear was not stable thorough the recording, thus we decided to re-reference to average to have a better signal. We believe this was the best choice for our dataset to help standardise the data (as recommended for example in Nunez et al., 2016: Nunez, M. D., Nunez, P. L., Srinivasan, R., Ombao, H., Linquist, M., Thompson, W., & Aston, J. (2016). Electroencephalography (EEG): neurophysics, experimental methods, and signal processing. Handbook of neuroimaging data analysis, 1, 175-197.)
This has been added to Supplementary information 4 with the rest of the detailed description of the data analysis procedure.

At the same time, it is not described whether any baseline correction was applied, something that is essential.

Yes, we did apply baseline correction. Data was epoched from 100 ms pre-stimulus to

500 ms post-stimulus onset - baseline correction was applied pre-stimulus at this stage, apologies for not mentioning this in the main text, it has now been included together with the rest of the pre-processing details in SI4.

Likewise, after applying methods related to artifact correction, it would be necessary to indicate the number of epochs used for analysis in each group to confirm a homogeneous distribution between groups in terms of available epochs.

For some participants we had to remove more epochs than for others, as in some cases removing the noisy components was not enough to have the data cleaned as we hoped. We retained a minimum of 100 trials for the deviant and a minimum of 500 for the standard for every participant. This has also been added in SI4.

I also consider it relevant for the authors to justify under what assumptions they believe that a lower MMN translates into an alteration.

If we understand the reviewer's comment correctly, the assumptions are articulated in the opening paragraph as expressed in the wider MMN and psychosis literature. If the comment is about the word "alteration" we have changed it to "difference".

In the discussion, it seems relevant to consider that some studies have emphasized the role of the attentional commitment component in patients with PD-VH. Specifically, we know that these patients show a predisposition in the ventral attentional system toward the evaluation of peripheral stimuli, thus favoring the interference of these stimuli over the target stimuli in a given task. In this sense, it is worth considering to what extent the reduction of MMN is a direct consequence of dysfunction in the systems dedicated to generating this component versus a consequence of the fact that the MMN-eliciting stimuli were not processed covertly but rather facilitated through attention mediated by the ventral attentional system. It would be interesting to show, in this sense, the possible effects in the time interval corresponding to P3a and P3b in both groups.

Thanks for the interesting suggestion. We now mention the P300 in the main text and the discussion (first paragraph) and have updated Figure 3 with these results.

Considering that the MoCA is not an instrument capable of detecting relatively subtle cognitive changes, but that the PD-VH group scores higher in inattention, I consider it relevant to consider the possible effect on the morphology of the ERPs caused by the fact that patients with OAB have greater cognitive impairment. Something that, on the other hand, depending on the type of hallucinations, is predictable.

We agree on the MoCA and we have discussed this in detail in our previous paper on cognitive deficits in PD psychosis (Montagnese et al,, 2022 https://doi.org/10.1016/j.cortex.2021.11.001). However, please note the inattention scores from the NMSQ are derived from self-reports: patients are asked if they feel that they lose focus while reading or watching a film, so it is difficult to relate this to their performance. Nevertheless, to explore the issue of attentional function further we have extracted the score for the attentional trials (maximum score = 6) of the MoCA and found no difference between the groups [F(1,27)=1.69, p=.204]. We repeated the analysis for the memory trails of the MoCA as after performing the P3a and P3b analysis we thought this might be of interest. We did not find differences for these scores either [F(1,27)=1, p=.33]. This demonstrates that there is no evidence for attentional deficits in our participants using the MoCA total score or item scores, which may speak to its "incapability of detecting relatively subtle cognitive changes".
We also find it interesting that the type of hallucinations might have an impact on the morphology of the ERP, however we think this requires a detailed analysis not only on the morphology of the ERP but the connectivity structure as well, which is the focus of a manuscript in preparation.
We have looked at VH scores in relation to some of the EEG measures but because of the relatively low numbers we have chosen not to overinterpret these findings.

Regarding the studies with Saractinib, given the very limited number of cases included, perhaps the work is somewhat detracted, and it may be necessary to consider not incorporating this analysis into this publication. It may be worthwhile to better address the issues regarding MMN between patient groups and perhaps wait until data from more cases are available to present the results of Saractinib.

Given the relative lack of progress for treatment development in PDP we wanted to express how EEG outcome measures could be useful in providing mechanistic support for potential novel mechanisms for treatment. The emphasis of the paper is very clearly on the main vMMN analysis, with the additional data showing how this can be used in mechanistic studies given much less space. We also highlight that Saracatinib is unfortunately no longer available so there we will not be able to present further cases.

****

Reply to comments from the Scientific Editorial team:

Many thanks for the comments, please find the relevant documents attached and the changes requested in the relevant documents.

-Please supply a graphical abstract. A graphical abstract is a visual representation of the central finding or methodology of the paper. It will be displayed in the table of contents. Authors should strive to make the graphical abstract informative, interesting, visually appealing, and straightforward (no caption needed, no abbreviations except gene names). The technical requirements for the graphical abstracts are:
- Size: 120 x 120 mm (300-600dpi) or 3000 x 3000 pixels
- Font: Arial 12-16 pt
- File type: tiff or PDF
- Aspect ratio: 1:1 preferred

This has been done and attached with the current submission.

-Please add an Abbreviated Summary (up to 50 words) of your paper that captures the main purpose and conclusions of the work. The summary should state the main results written in the third person ('[1st author surname] et al. report that ...') and conclusion. This summary will be used in the contents list online and should not contain any abbreviations other than those on the accepted abbreviations list and accepted gene/protein names, making it accessible to the non-specialist. Please upload it as a Word document. Allowed abbreviations: AIDS; ANOVA; AMPA; ATP; A,T,C,G; CNS; CSF; CT; DNA; ECG; EEG; EMG; GABA; HIV; MRI; NMDA; PET; PCR; RNA.

This has been done and attached with the current submission.

-Thank you for providing a checklist. For all sections, please include the page numbers corresponding to the items listed in the reporting guideline checklist.
This has been done and we have also added a study flowchart at the end of the supplementary materials for study1 and 3.

-Thank you for providing a paragraph describing statistical analyses. Please label the paragraph with a subheader 'statistical analysis' in the 'Materials and Methods' section.
This has been done.

-Data availability: Thank you for providing a data availability statement. If applicable, please provide the codes generated and used in this work within the supplementary material or through a repository, whose link has to be made available within the data availability statement.

The only code uploaded is the code generated for this study: for eeglab we describe the

methods in detail and follow the standard pre-processing pipeline thus we do not provide the eeglab code. We provide the ggplot2 code used to produce the waveform plots provided in Figure5 and 7.

-Figures 1 and 2: Please ensure that all abbreviations are spelt out in the figure legend. This has been done.

-Figures 2 and 6c, Supplementary figure 2b legends: Please provide details of the statistical analysis performed, the experimental unit (n= mice, cells, wells, plate downs, etc)) and the number of data-points for each group analysed in these plots, and if the number is <10 show the single data-points. Please note our policy on pseudoreplication, we do not accept analyses where individual cells are the experimental unit when there is likely biological variability between animals, culture replicates, etc. Please see instructions for authors for more details. For this type of data, we require either that a mean/median be taken per animal/culture replicate followed by statistical analyses or that more sophisticated analyses are performed such as mixed effects modelling which can account for random variables.
This has been done.

-Figures 3, 4, 5, 6b and 7, Supplementary figures 3, 8a, 8b, 10e legends: Please provide details of the statistical analysis performed, the experimental unit (n= mice, cells, wells, plate downs, etc). This has been done.
In addition SI8 is now updated: a) additional parietal analyses; 8bindividual VEPs; c) between-group EEGlab study analysis – as for the P300 analysis we added some waveform plots to Figure3 the previous SI8b with the within group plot for additional electrodes was redundant.

-Figure 4: Please revisit your colour choice in the figure as the green-red combination is not colour-blind friendly. We have checked all our figures for this possible issue and: 1) we have also removed the green-red shaded area from figure 6c and tweaked the color palette to avoid the presence of orange/red-green; for figure 4, we changed the colours of the dotted lines and the topography maps: we used the turbo palette which is very similar to the traditional one (jet) as it is colour blind friendly (turbo has been tested and found well perceived except for Achromatopsia - total colour blindness - we used the Coblis website for this purpose). If another option e.g. parula is preferred we are happy to change this or to devise our own colour palette.
