# Supplementary material for: Visual mismatch negativity in Parkinson's psychosis and potential for testing treatment mechanisms
Source: Brain Commun. 2024 Sep 3;6(5):fcae291. doi: 10.1093/braincomms/fcae291 (PMC11443450; doi:10.1093/braincomms/fcae291)
Supplement: fcae291_Supplementary_Data [file Revision_1_ Response_to_reviewers_comments.pdf › Revision_1_ Response_to_reviewers_comments.pdf]

Response to Reviewers: Revision One

We wish to thank you for the time spent on our manuscript. We also wish to thank the reviewers for the time spent on the manuscript and the comments which have been very helpful and constructive and have helped improve the manuscript.
Many thanks for the comments, we reply to each of the requests below. We also confirm that we are happy to have the reviewers' comments and our responses published.

Responses to comments from the Scientific Editor:

1) A new version of the graphical abstract has been uploaded. Let us know whether it complies with your requests.

2) please, clarify the section of the manuscript where the R codes used have been reported, as I cannot find them – The files are on github in a dedicated folder https://github.com/VMiri/PDP_MMN ; once the manuscript is published more information about the study will be added (e.g. doi etc)

- please, make sure to increase the font of numbers and letters, and the details of the plots to make them readable. This has been done, specifically the graphical abstracts and Figures 3, 4 and 5, where the text on the axes was not large enough have been updated.

- please, make sure to include the statistical analysis run on the data in figures, in the corresponding legend. This has been done .

- please, make sure that each figure and table fits one A4 page. – This has been done.

- regarding the supplementary material, please, make sure to not consider single information as one figure. We checked all the figures in the supplement for this. All are necessary and have a purpose and are referred to in the main text.
