# Supplementary material for: Visual mismatch negativity in Parkinson's psychosis and potential for testing treatment mechanisms
Source: Brain Commun. 2024 Sep 3;6(5):fcae291. doi: 10.1093/braincomms/fcae291 (PMC11443450; doi:10.1093/braincomms/fcae291)
Supplement: fcae291_Supplementary_Data [file Revision_2_ Response_to_reviewers_comments.pdf › Revision_2_ Response_to_reviewers_comments.pdf]

Response to Reviewers: Revision Two

12-Jun-2024

Many thanks for the time spent on our manuscript. I have addressed all the comments of the Scientific Editor regarding the figures and supplementary information.
I have renamed the supplementary figures from 1 to 11 rather than numbering them with the number of the supplementary information they were associated to (this was how I had numbered them initially) - however in responding to the comments I am referring to the original figure numbers.
Please find a point by point response to the comments:

- Fig4, Supplementary Fig2b and Supplementary Fig3: please report in the figure legend the test used and relevant test statistic values alongside p values (for example F or t values for ANOVAs and t-tests).
This has been done.

- Fig6b and Supp Fig7 - : please, make sure that all the words in the image are readable.

This has been done. Figure 6b: the fonts of the correlation coefficients in the corrplot have been increased; Supplementary Figure 7 has been reworked in order to fit to A4 format and to increase legibility.

- SuppFig8-10: please, make sure that each figure fits an A4 page.
This has been verified.

- The supplementary material misses Supp Fig1, Supp Fig4,6-7.
I have now renamed all supplementary figures from 1 to 10 rather than numbering them with the number of the supplementary information they were associated to (this was how I had numbered them initially).

- Please, label the last image of the supplementary material file which includes a flow chart with a figure name.
This has been done. It is now Supplementary figure 11.
