# Supplementary material for: Visual mismatch negativity in Parkinson's psychosis and potential for testing treatment mechanisms
Source: Brain Commun. 2024 Sep 3;6(5):fcae291. doi: 10.1093/braincomms/fcae291 (PMC11443450; doi:10.1093/braincomms/fcae291)
Supplement: fcae291_Supplementary_Data [file Revision_3_ Response_to_reviewers_comments.pdf › Revision_3_ Response_to_reviewers_comments.pdf]

Response to Reviewers: Revision Three

Dear Prof. Spires-Jones and Dr. Marcotti,

many thanks for your response and for the time spent on our manuscript. Apologies for missing these points, this has now been amended.

The description of supplementary Figure 3 now reads "Figure 3. On the left panel PD-noVH (18), on the right PD-VH (20). Violin plots represent distribution of responses: in yellow the % of correct responses to the cross change (bigger or smaller); in pink the % of errors and in purple the misses. We carried out a one-way ANOVA to analyse the difference in performance in the two groups, finding that PD-VH performed worse than PD-noVH [$F(1,34) = 10.64$, $p = .003$] but the two groups did not differ in the misses [$F(1,34) = 2.86$, $p = .100$]."

Figure 7 in the main text has beed edited, with a bigger font used for y-axes of the MMN plots.

I hope these edits comply with your requests.

Thanking you once again on behalf of all authors of this manuscript,
Sincerely

Miriam Vignando
