# Supplementary material for: Visual mismatch negativity in Parkinson's psychosis and potential for testing treatment mechanisms
Source: Brain Commun. 2024 Sep 3;6(5):fcae291. doi: 10.1093/braincomms/fcae291 (PMC11443450; doi:10.1093/braincomms/fcae291)
Supplement: fcae291_Supplementary_Data [file Revision_3_decision_letter.pdf › Revision_3_decision_letter.pdf]

Decision Letter Four: Revision Three

24-Jun-2024

Dear Dr. Vignando,

Thank you for your recent submission BRAINCOM-2023-567.R3
entitled "Visual mismatch negativity in Parkinson's psychosis and potential for testing treatment mechanisms" to Brain Communications.

I am pleased to let you know that we would like to provisionally accept your manuscript entitled "Visual mismatch negativity in Parkinson's psychosis and potential for testing treatment mechanisms" for publication in Brain Communications. There are still some minor points for you to address before final acceptance, but further peer-review will not be necessary.

Please would you respond to the comments shown below from reviewers and/or our Scientific Editor (regarding our requirements for presentation of scientific data at Brain Communications) and submit a revised version.

Please format your revised manuscript according to our Instructions to Authors
https://academic.oup.com/braincomms/pages/General_Instructions
A summary of key points is attached to help with this. Please note that there can be substantial delays to publication if you don't resubmit in line with our requirements.

To revise your manuscript, click the link below or log into https://mc.manuscriptcentral.com/braincom and enter your Author Centre, where you will find your manuscript title listed under "Manuscripts with Decisions." Under "Actions," click on "Create a Revision." Your manuscript number will be appended to denote a revision.

*** PLEASE NOTE: This is a two-step process. After clicking on the link, you will be directed to a webpage to confirm. ***

https://mc.manuscriptcentral.com/braincom?URL_MASK=cc5104b10d764adabdaa642eecd83013

Please upload a revised manuscript with the changes you have made shown either with "track changes" or in coloured text.

When submitting your revised manuscript, you will be asked to respond to the

reviewer(s) and editor's comments. Please be as specific as possible in your response to all of the comments.

The editorial team at Brain Communications also aims to increase transparency in scientific publishing and to demonstrate the value that peer review brings to the scientific process. Please therefore consider whether you are willing to have reviewer comments on your paper and your response published as supplementary data with the paper if it is accepted. This is entirely optional and will only be done if both the authors and reviewers agree. When submitting your revision you will have the opportunity to update the answer you entered previously.

IMPORTANT: Your original files are available to you when you upload your revised manuscript. Please delete any redundant files before completing the submission.

Because we are trying to facilitate timely publication of manuscripts submitted to Brain Communications, we would appreciate your revised manuscript as soon as possible. If it is not possible for you to submit your revision within 3 months, the option to submit a revision will expire, and we will consider your revised paper as a new submission. However, if you need a little more time, please get in touch and we can extend this deadline for you.

Please note that Brain Communications is a fully open access journal, and if your article is accepted for publication, an open access charge will be payable (this does not apply to letters and scientific commentaries). Details can be found in our Instructions for Authors: https://academic.oup.com/braincomms/pages/General_Instructions

Once again, thank you for submitting your manuscript to Brain Communications and I look forward to receiving your revision.

Best wishes

Dr. Manuela Marescotti
Scientific Editor, Brain Communications

Professor Tara Spires-Jones
Founding Editor, Brain Communications

Comments from the Scientific Editor:

thank you for addressing the almost all my queries, however there are still a couple of points to clarify, as follows:

- Supp Fig3: please, report in the figure legend the statistical analysis performed.

- Fig7: please, increase the font of the y-axes labels.
